# The Protein Cost of Metabolic Fluxes: Prediction from Enzymatic Rate Laws and Cost Minimization

**DOI:** 10.1371/journal.pcbi.1005167

**Published:** 2016-11-03

**Authors:** Elad Noor, Avi Flamholz, Arren Bar-Even, Dan Davidi, Ron Milo, Wolfram Liebermeister

**Affiliations:** 1 Institute of Molecular Systems Biology, Eidgenössische Technische Hochschule, Zürich, Switzerland; 2 Department of Molecular and Cellular Biology, University of California, Berkeley, Berkeley, California, United States of America; 3 Max Planck Institute for Molecular Plant Physiology, Golm, Germany; 4 Department of Plant Sciences, The Weizmann Institute of Science, Rehovot, Israel; 5 Institute of Biochemistry, Charité Universitätsmedizin Berlin, Berlin, Germany; University of Michigan, UNITED STATES

## Abstract

Bacterial growth depends crucially on metabolic fluxes, which are limited by the cell’s capacity to maintain metabolic enzymes. The necessary enzyme amount per unit flux is a major determinant of metabolic strategies both in evolution and bioengineering. It depends on enzyme parameters (such as *k*_*cat*_ and *K*_*M*_ constants), but also on metabolite concentrations. Moreover, similar amounts of different enzymes might incur different costs for the cell, depending on enzyme-specific properties such as protein size and half-life. Here, we developed enzyme cost minimization (ECM), a scalable method for computing enzyme amounts that support a given metabolic flux at a minimal protein cost. The complex interplay of enzyme and metabolite concentrations, e.g. through thermodynamic driving forces and enzyme saturation, would make it hard to solve this optimization problem directly. By treating enzyme cost as a function of metabolite levels, we formulated ECM as a numerically tractable, convex optimization problem. Its tiered approach allows for building models at different levels of detail, depending on the amount of available data. Validating our method with measured metabolite and protein levels in *E. coli* central metabolism, we found typical prediction fold errors of 4.1 and 2.6, respectively, for the two kinds of data. This result from the cost-optimized metabolic state is significantly better than randomly sampled metabolite profiles, supporting the hypothesis that enzyme cost is important for the fitness of *E. coli*. ECM can be used to predict enzyme levels and protein cost in natural and engineered pathways, and could be a valuable computational tool to assist metabolic engineering projects. Furthermore, it establishes a direct connection between protein cost and thermodynamics, and provides a physically plausible and computationally tractable way to include enzyme kinetics into constraint-based metabolic models, where kinetics have usually been ignored or oversimplified.

## Author Summary

“Enzyme cost”, the amount of protein needed for a given metabolic flux, is crucial for the metabolic choices cells have to make. However, due to the technical limitations of linear optimization methods, this cost has traditionally been ignored by constraint-based metabolic models such as Flux Balance Analysis. On the other hand, more detailed kinetic models which use ordinary differential equations to simulate fluxes for different choices of enzyme allocation, are computationally demanding and not scalable enough. In this work, we developed a method which utilizes the full kinetic model to predict steady-state enzyme costs, using a scalable and robust algorithm based on convex optimization. We show that the minimization of enzyme cost is a meaningful optimality principle by comparing our predictions to measured enzyme and metabolite levels in exponentially growing *E. coli*. This method could be used to quantify the enzyme cost of many other pathways and explain why evolution has selected some low-yield metabolic strategies, including aerobic fermentation in yeast and cancer cells. Furthermore, future metabolic engineering projects could benefit from our method by choosing pathways that reduce the total amount of enzyme required for the synthesis of a value-added product.

This is a *PLOS Computational Biology* Methods paper.

## Introduction

The biochemical world is remarkably diverse, and new pathways and chemicals are still discovered routinely. Even for extensively studied model organisms like *E. coli*, efforts to exhaustively map metabolic networks are only nearing completion on the stoichiometric level. Our understanding of metabolic fluxes, their dynamic regulation and their connection to cell fitness is far from perfect [1]. Furthermore, the rational design of novel and efficient metabolic pathways remains a substantial challenge and metabolic engineering projects require considerable efforts even for relatively simple metabolic tasks. Among the different possible criteria [2], one key to understanding the choices of metabolic routes, both in naturally evolved and engineered organisms, may be enzyme cost. Quite often, cells use metabolic pathways in ways that seem irrational, as in the case of aerobic fermentation (known as the Crabtree effect in yeast or the Warburg effect in cancer cells [3]). However, apparently yield-inefficient fluxes can sometimes be explained by an economic use of enzyme resources [4, 5]. It is posited that pathway structures that require too much enzyme per unit flux will be out-competed during evolution and will not be efficient for biotechnological applications. Thus, a quantitative analysis of resource investment in enzyme production, predicting the amount of enzyme needed to support a given flux, would be valuable in aiding the rational design of metabolic pathways.

To understand why specific enzymes or pathways occupy larger or smaller areas of the proteome [6], we could proceed in two steps, determining first the metabolic fluxes and then enzyme levels needed to realize these fluxes. Metabolic fluxes can be measured through isotope-labeled tracer experiments in combination with computational modeling. Methods for flux prediction *ab initio* rely on mechanistic aspects (chemical mass balances and kinetics) and economic aspects (cost and benefit of pathway fluxes) and combine them in different ways. Constraint-based methods like Flux Balance Analysis (FBA) determine fluxes by requiring steady states—i.e., fluxes must be such that internal metabolite levels remain constant in time—and assuming that natural selection maximizes some benefit function (e.g., maximal yield of biomass). Several optimality criteria for fluxes can be combined by multi-objective optimization [1, 7]. In some cases, the second law of thermodynamics is used to put further constraints on fluxes or metabolite levels [8–11]. Some extensions of FBA [12–14] use metabolite log-concentrations as extra variables and constrain fluxes to flow only in the direction of thermodynamic driving forces, i.e., towards lower chemical potentials. Thermodynamics links between flux directions and reactant concentrations, and thus physiological bounds on metabolite levels are translated into restrictions on flux directions. These links between fluxes and metabolite concentrations hold independently of specific reaction kinetics. The relationship between fluxes and metabolite concentrations can be used also in the opposite direction—i.e. given all flux directions, certain metabolite profiles can be excluded [14]. The set of feasible metabolite profiles can be depicted as a polytope in the space of metabolites’ log-concentrations. To further narrow down the metabolite concentration profiles, the Max-min Driving Force (MDF) method [15] chooses profiles that ensure sufficient driving forces, thus keeping reactions distant from chemical equilibrium.

Typically, constraint-based models bypass the non-linearity of enzyme kinetics by focusing on the feasible flux space and assess the relative benefits of different flux distributions. Thus, such models do well in simulating binary perturbations such as reaction knockouts or nutrient deprivation. On the other hand, they were not designed to predict the necessary enzyme levels and the cost of making and maintaining the enzymes, and therefore perform poorly at these tasks. Here we ask: how can we estimate the amount of protein required to sustain a given flux through a reaction or pathway? It is often assumed that the flux through a reaction is proportional to the enzyme level. FBA methods use this assumption to translate enzyme expression, as a proxy for protein burden, into flux bounds or linear flux cost functions [16]. For practical reasons (computational tractability and lack of detailed knowledge), flux costs are often represented by the sum of absolute fluxes [17, 18]. To obtain better proxies of protein demand and related cellular burdens, fluxes have been weighted by “flux burdens” that account for different catalytic constants *k*_cat_ [2, 19], protein size and lifetime [20], or equilibrium constants [17]. In reality, however, enzyme demand does not only depend on fluxes, but also on metabolite levels, which in turn are determined by the non-linear kinetics of all active enzymes and transporters. Therefore, it is not only the choice of numerical cost weights, but the very relation between enzyme amounts and fluxes that needs to be clarified.

**Fig 1:**
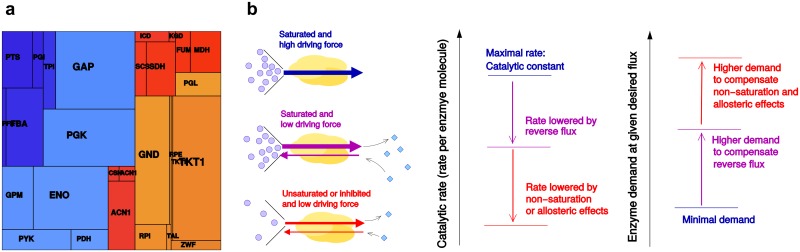
Enzyme cost in metabolism. (a) Measured enzyme levels in *E. coli* central metabolism (molecule counts displayed as rectangle areas). Colors correspond to the network graphics in Fig 3. To predict such protein levels, and to explain the differences between enzymes, we start from known metabolic fluxes and assume that these fluxes are realized by a cost-optimal distribution of enzyme levels. (b) Enzyme-specific flux depends on a number of physical factors. Under ideal conditions, an enzyme molecule catalyzes its reaction at a maximal rate given by the enzyme’s forward catalytic constant (top left). The rate is reduced by microscopic reverse fluxes (center left) and by incomplete saturation with substrate (causing waiting times between reaction events) or by allosteric inhibition or incomplete activation (bottom left). With lower catalytic rates (center), realizing the same metabolic flux requires larger amounts of enzyme (right).

For a simple estimate, we can assume that each enzyme molecule works at its maximal rate, the catalytic constant *k*_cat_. In this case, enzyme demand is given by the flux divided by the catalytic constant [2, 19]. To translate enzyme demand into cost, the different sizes or effective lifetimes of enzymes can be considered [20]. The notion of Pathway Specific Activity [2] applies this principle to the efficiency of entire pathways (assuming that enzyme levels are optimally distributed), and provides a direct way to compare between alternative pathways. However, by assuming that enzymes operate at their maximal capacity, we underestimate the true enzyme demand (see Fig 1). Enzymes typically do not operate at full capacity. This is due to backward fluxes, incomplete substrate saturation, allosteric regulation, and regulatory post-translational modifications. Below, we will refer to allosteric regulation only, but other types of post-translational regulation, e.g., by phosphorylation, could be treated similarly. The relative backward fluxes depend on the ratio between product and substrate concentrations, called the mass-action ratio. Whenever the mass-action ratio deviates from its equilibrium value, the equilibrium constant, this deviation can be conceptualized as a thermodynamic driving force. The driving force determines the relative backward flux and thus affects reaction kinetics and enzymatic efficiency [21, 22]. With smaller forces, the relative backward flux increases, enzyme usage becomes less efficient, and enzyme demand increases [4, 23]—a situation that, in models, can be avoided by applying the MDF method. In fact, a cost increase due to backward fluxes can be included in the principle of minimal fluxes in FBA [17]. However, metabolites do not only affect thermodynamic forces, as acknowledged in thermodynamic FBA, but also affect kinetics as reactants and allosteric effectors. While the relative backward fluxes depend on thermodynamic forces, the forward flux depends on the availability of substrate molecules. At sub-saturating substrate levels, enzyme molecules spend some time waiting for substrate molecules, thus reducing their average catalyzed flux. Likewise, the presence of reaction product can reduce the fraction of enzyme molecules available for catalysis in the direction of pathway flux.

Thus, converting metabolic fluxes into enzyme demand can be difficult because enzymes may not realize their maximal capacity. Since reduced enzyme efficiency is mostly due to metabolite concentrations, enzyme and metabolite profiles must be considered together. This quickly becomes a cyclic inference problem because steady-state metabolite levels depend again on enzyme profiles. Since many metabolites (e.g., co-factors like ATP) participate in multiple pathways, enzyme demands may be coupled across the entire metabolic network. Moreover, there may be many possible enzyme and metabolite profiles that realize the same flux distribution. To determine a single solution, one can make the assumption that the most reasonable enzyme profile for realizing a given flux is the one with the minimum associated cost. This assumption may be justified if we focus on biological systems shaped by evolution, or on engineered pathways that should be efficient. A direct optimization of enzyme levels can be difficult, but there is a tractable approach in which metabolite levels are treated as free variables, which determine the enzyme levels, and therefore enzyme cost. This approach, together with a minimization of metabolite concentrations [24], has been previously applied to predict enzyme and metabolite levels in metabolic systems [23] and to compare structural variants of glycolysis by the cost of ATP production [4].

However, to make such optimization schemes generally applicable, some open problems need to be addressed. First, our knowledge of the kinetic rate laws and parameters contains large gaps for the vast majority of enzymes [25], and combining rate constants from different sources may lead to inconsistent models [26, 27]. Second, the optimization problem may be computationally challenging for large networks and realistic rate laws. To turn enzyme cost minimization into a generally applicable method, we address a number of questions: (i) When setting up models for enzyme cost prediction, how can we deal with missing, uncertain, or conflicting data on rate constants? Are there approximations, for example based on thermodynamics, that yield good predictions with fewer input parameters? (ii) How do factors such as the *k*_cat_, driving force, or rate law affect enzyme demand, and how do they shape the optimal metabolic state? (iii) How can enzyme optimization be formulated as a numerically tractable optimality problem? Existing approaches for flux and enzyme prediction have focused on different aspects (stationary state, energetics [8, 28], kinetics [23, 29], molecular crowding [19, 30], as well as enzyme cost [31, 32], metabolite cost [24], or flux cost [17]). The new approach, which uses a modular kinetic rate law to translate fluxes into enzyme demand, shows how these approaches are logically related, and how heuristic assumptions by other methods, e.g. an avoidance of small driving forces, follow from enzyme economy as a general principle (S1 Text section 4). We show that enzyme cost minimization is closely related to cost-benefit approaches, which treat cell fitness as a function of enzyme levels [31, 33–37]. Some general results of these approaches, e.g., relationships between enzyme costs and metabolic control coefficients, can be reproduced.

## Results

### Enzyme cost landscape of a metabolic pathway

**Fig 2:**
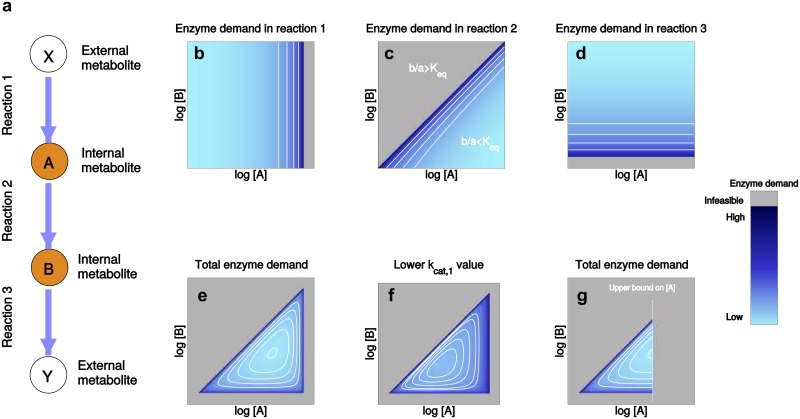
Enzyme demand in a metabolic pathway. (a) Pathway with reversible Michaelis-Menten kinetics (equilibrium constants, catalytic constants, and *K*_M_ values are set to values of 1, [A] and [B] denote the variable concentrations of intermediates A and B in mM). The external metabolite levels [X] and [Y] are fixed. Plots (b)-(d) show the enzyme demand of reactions 1, 2, and 3 at given flux *v* = 1 according to Eq (2). Grey regions represent infeasible metabolite profiles. At the edges of the feasible region (where A and B are close to chemical equilibrium), the thermodynamic driving force goes to zero. Since small forces must be compensated by high enzyme levels, edges of the feasible region are always dark blue. For example, in reaction 1 (panel (b)), enzyme demand increases with the level of A (x-axis) and goes to infinity as the mass-action ratio [*A*]/[*X*] approaches the equilibrium constant (where the driving force vanishes). (e) Total enzyme demand, obtained by summing all enzyme levels. The metabolite polytope—the intersection of feasible regions for all reactions—is a triangle, and enzyme demand is a convex function on this triangle. The point of minimum total enzyme demand defines the optimal metabolite levels and optimal enzyme levels. (f) As the *k*_cat_ value of the first reaction is lowered by a factor of 5, states close to the triangle edge of reaction 1 become more expensive and the optimum point is shifted away from the edge. (g) The same model with a physiological upper bound on the concentration [A]. The bound defines a new triangle edge. Since this edge is not caused by thermodynamics, it can contain an optimum point, in which driving forces are far from zero and enzyme costs are kept low.

Given a pathway flux profile and a kinetic model of the pathway, one can predict the enzyme demand by assuming that cells minimize the enzyme cost in that pathway. A reaction rate *v* = *E* ⋅ *r*(**c**) depends on enzyme level *E* and metabolite concentrations *c*_*i*_ through the enzymatic rate law, *r*(**c**). If the metabolite levels were known, we could directly compute enzyme demands *E* = *v*/*r*(**c**) from fluxes, and similarly calculate the flux-specific enzyme demand *E*/*v* = 1/*r*(**c**). However, metabolite levels are often unknown and vary between experimental conditions. Therefore, there can be many solutions for *E* and **c** realizing one flux distribution. To select one of them, we employ an optimality principle: we define an enzyme cost function (for instance, total enzyme mass) and choose the enzyme profile with the lowest cost while restricting the metabolite levels to physiological ranges and imposing thermodynamic constraints. As we shall see below, the optimal solution is in many cases unique. Let us demonstrate this with a simple example (Fig 2a). In the pathway *X* ⇌ *A* ⇌ *B* ⇌ *Y*, the external metabolite levels [X] and [Y] are fixed and given, while the intermediate levels [A] and [B] need to be found. As rate laws for all three reactions, we use reversible Michaelis-Menten (MM) kinetics

$$v\left(s,p,E\right)=E\;\frac{k_{\text{cat}}^{+}\;s/K_{S}-k_{\text{cat}}^{-}\;p/K_{P}}{1+s/K_{S}+p/K_{P}}$$ (1)

with enzyme level *E*, substrate and product levels *s* and *p*, turnover rates $k_{\text{cat}}^{+}$ and $k_{\text{cat}}^{-}$, and Michaelis constants *K*_S_ and *K*_P_. In kinetic modeling, steady-state concentrations would usually be obtained from given enzyme levels and initial conditions through numerical integration. Here, instead, we fix a desired pathway flux *v* and compute the enzyme demand as a function of metabolite levels:

$$E\left(s,p,v\right)=v\;\frac{1+s/K_{S}+p/K_{P}}{k_{\text{cat}}^{+}\;s/K_{S}-k_{\text{cat}}^{-}\;p/K_{P}}.$$ (2)

Fig 2 shows how the enzyme demand in each reaction depends on the logarithmic reactant concentrations. To obtain a positive flux, substrate levels *s* and product levels *p* must be restricted: for example, to allow for a positive flux in reaction 2, the rate law numerator $k_{\text{cat}}^{+}\;\left[A\right]/K_{S}-k_{\text{cat}}^{-}\;\left[B\right]/K_{P}$ must be positive. This implies that [*B*]/[*A*] < *K*_eq_ where the reaction’s equilibrium constant *K*_eq_ is determined by the Haldane relationship, $K_{\text{eq}}=\left(k_{\text{cat}}^{+}/k_{\text{cat}}^{-}\right)\cdot \left(K_{P}/K_{S}\right)$. With all model parameters set to 1, we obtain the constraint [*B*]/[*A*] < 1, i.e., ln[*B*] − ln[*A*] < 0, putting a linear boundary on the feasible region (Fig 2(c)). Close to chemical equilibrium ([*B*]/[*A*] ≈ *K*_eq_), the enzyme demand *E*_2_ approaches infinity. Beyond the boundary ([*B*]/[*A*] > *K*_eq_) no positive flux can be achieved (grey region). Such a threshold exists for each reaction (see Fig 2b–2d). The remaining feasible metabolite profiles form a triangle in log-concentration space, which we call *metabolite polytope* P (Fig 2e), and Eq (2) yields the total enzyme demand *E*_tot_ = *E*_1_ + *E*_2_ + *E*_3_, as a function on the metabolite polytope. The demand increases steeply towards the edges and becomes minimal in the center. The minimum point marks the optimal metabolite profile, and via Eq (2) we obtain the resulting optimal enzyme profile.

The metabolite polytope and the large enzyme demand at its boundaries follow directly from thermodynamics. To see this, we consider the unitless *thermodynamic driving force* Θ = −Δ_r_*G*′/*RT* [38] derived from the reaction Gibbs free energy Δ_r_*G*′. For a given mass-action ratio *Q* = [*B*]/[*A*], the thermodynamic force can also be written as Θ = ln(*K*_eq_/*Q*), i.e., the driving force is positive whenever *Q* < *K*_eq_, and it vanishes if *Q* = *K*_eq_. How is this force related to enzyme cost? A reaction’s net flux is given by the difference *v* = *v*^+^ − *v*^−^ of forward and backward fluxes, and the ratio *v*^+^/*v*^−^ depends on the driving force as *v*^+^/*v*^−^ = e^Θ^. Thus, only a fraction *v*/*v*^+^ = 1 − e^−Θ^ of the forward flux acts as a net flux, while the remaining forward flux is canceled by the backward flux (Figure A in S1 Text). Close to chemical equilibrium, where the mass-action ratio approaches the equilibrium constant, i.e. *Q* → *K*_eq_, the driving force goes to zero, the reaction’s backward flux increases, and the flux per unit enzyme level drops. This is what happens at the triangle edges in Fig 2. Exactly on the edge, the driving force vanishes and no enzyme level, no matter how large, can support a positive flux. The quantitative cost depends on model parameters: for example, lowering a *k*_cat_ value increases the cost of each enzyme unit, making the polytope boundary steeper and thus the optimum is shifted away from the boundary (see Fig 2f and Figure B in S1 Text).

### Enzyme cost as a function of metabolite profiles

The prediction of optimal metabolite and enzyme levels can be extended to models with general rate laws and complex network structures. In general, enzyme demand depends not only on driving forces and *k*_cat_ values, but also on the kinetic rate law, which includes Michaelis-Menten constants (*K*_M_) and allosteric regulation. Thus, one must model these factors using the available kinetic information [39, 40], or approximate them when the information is not available. For some of these parameters, genome-scale prediction methods exist [41, 42]. The rate of a reaction depends on enzyme level *E*, forward catalytic constant $k_{\text{cat}}^{+}$ (i.e. the maximal possible forward rate per unit of enzyme, in s^−1^), driving force (i.e., the ratio of forward and backward fluxes), and on kinetic effects such as substrate saturation or allosteric regulation. If all active fluxes are positive, reversible rate laws like the Michaelis-Menten kinetics in Eq (1) can be factorized as [22]

$$v=E\cdot k_{\text{cat}}^{+}\cdot \eta^{\text{rev}}\cdot \eta^{\text{kin}}.$$ (3)

Negative fluxes, which would complicate this formula, can be avoided by orienting all reactions in the direction of fluxes. The reversible Michaelis-Menten rate law Eq (1), for example, can be written in this separable form [22]:

$$v=E\;k_{\text{cat}}^{+}\;\frac{s/K_{S}\;\left(1-\frac{k_{\text{cat}}^{-}}{k_{\text{cat}}^{+}}\frac{p/K_{P}}{s/K_{S}}\right)}{1+s/K_{S}+p/K_{P}}=E\;k_{\text{cat}}^{+}\;\underset{\eta^{\text{rev}}}{\underset{︸}{\left(1-\frac{k_{\text{cat}}^{-}}{k_{\text{cat}}^{+}}\frac{p/K_{P}}{s/K_{S}}\right)}}\;\underset{\eta^{\text{kin}}}{\underset{︸}{\frac{s/K_{S}}{1+s/K_{S}+p/K_{P}}}},$$ (4)

and similar factorizations exist for reactions of any stoichiometry (see S1 Text section 2.2). The term $E\cdot k_{\text{cat}}^{+}$ describes the maximal reaction velocity, which is reduced, depending on metabolite levels, by condition-specific factors *η*^rev^ and *η*^kin^ (see Fig 1b), accounting for backward fluxes, incomplete substrate saturation, or saturation with product (see Table 1 for a summary of all mathematical symbols used throughout this paper). The reversibility factor *η*^rev^ can be expressed in terms of the driving force Θ ≡ −Δ_r_*G*′/*RT* by the general formula *η*^rev^ = 1 − e^−Θ^, which also applies to reactions with multiple substrates and products [22]. The factor *η*^kin^ depends on the rate law and thus on the enzyme mechanism considered (see S1 Text section 2.2). In some cases, it could be convenient to subdivide Eq (3) even further: the $k_{\text{cat}}^{+}$ value can be decomposed into a product $k_{\text{cat}}^{+}=k_{\text{cat}}^{\infty }\cdot \eta^{\text{cat}}$, where $k_{\text{cat}}^{\infty }$ denotes the catalytic constant of a hypothetical, infinitely fast enzyme whose rate is only limited by substrate diffusion. The enzyme-specific, constant factor $\eta^{\text{cat}}=k_{\text{cat}}^{+}/k_{\text{cat}}^{\infty }$ is a unitless number between 0 and 1. A realistic value of $k_{\text{cat}}^{\infty }=10^{8}$ *s*^−1^ can be obtained by considering a very fast enzymatic reaction, the breakdown of water structure around a polymer [43]. Furthermore, with some rate laws, *η*^kin^ can be further decomposed into *η*^kin^ = *η*^sat^ ⋅ *η*^reg^, where *η*^reg^ refers to certain types of allosteric regulation (see example in Methods).

**Table 1:** Mathematical symbols used. The fitness unit Darwin (D) is a proxy for the different fitness units used in cell models. Reaction must be orientated in such a way that all fluxes are positive. To define metabolite log-concentrations, we use the standard concentration *c*_*σ*_ = 1 mM. For a more comprehensive list of mathematical symbols used in ECM, see Table C in S1 Text.

Name	Symbol	Unit
Flux	*v*_*l*_	mM/s
Metabolite level	*c*_*i*_	mM
Logarithmic metabolite level	*x*_*i*_ = ln(*c*_*i*_/*c*_*σ*_)	unitless
Enzyme level	*E*_*l*_	mM
Reaction rate	*v*_*l*_(*E*_*l*_,**c**) = *E*_*l*_ ⋅ *r*_*l*_(**c**)	mM/s
Catalytic rate	*r*_*l*_ = *v*_*l*_/*E*_*l*_	1/s
Scaled reactant elasticity	$E_{li}$	unitless
Gibbs energy of formation (std. chemical potential)	${G^{\prime }}_{i}^{\circ }$	kJ/mol
Reaction Gibbs energy	$\Delta_{r}G_{l}^{\prime }=\Delta_{r}{G_{l}^{\prime }}^{\circ }+RT\sum_{i}\;n_{il}\;\;\ln\;c_{i}$	kJ/mol
Driving force	$\Theta_{l}=-\Delta_{r}G_{l}^{\prime }/RT$	unitless
Forward/backward catalytic constant	$k_{\text{cat}}^{+},k_{\text{cat}}^{-}$	1/s
Diffusion-limited catalytic constant	$k_{\text{cat}}^{\infty }$	1/s
Michaelis-Menten constant	*K*_*li*_	mM
Protein mass	*m*_*l*_	Da
Efficiency factors	*η*^cat^, *η*^rev^, *η*^kin^, *η*^sat^, *η*^reg^	unitless
Enzyme cost	$h(E)=\sum_{l}\;h_{E_{l}}\;E_{l}$	D
Enzyme burden	$h_{E_{l}}$	D/mM
Enzyme-induced metabolite cost	*q*(**x**, **v**) = *h*(*E*(**x**, **v**))	D
Flux-specific cost	$a_{v_{l}}$	D/(mM/s)
Baseline flux-specific cost	$a_{v_{l}}^{\text{cat}}$	D/(mM/s)

The factorization in Eq 3, and any finer subdivision into factors, will lead to a subdivision of enzyme demands. Enzyme demand can be quantified as a concentration (e.g., enzyme molecules per volume) or mass concentration (where enzyme molecules are weighted by their molecular weights). If rate laws, fluxes, and metabolite levels are known, the enzyme demand of a single reaction *l* follows from Eq 3 as

$$E_{l}\left(c,v_{l}\right)=v_{l}\cdot \frac{1}{k_{\text{cat},l}^{+}}\cdot \frac{1}{\eta_{l}^{\text{rev}}\left(\Theta \left(c\right)\right)}\cdot \frac{1}{\eta_{l}^{\text{sat}}\left(c\right)}\cdot \frac{1}{\eta_{l}^{\text{reg}}\left(c\right)}.$$ (5)

To determine the enzyme demand of an entire pathway, we sum over all reactions: *E*_tot_ = ∑_*l*_ *E*_*l*_. Based on its enzyme demands *E*_*l*_, we can associate each metabolic flux with an enzyme cost $q=\sum_{l}\;h_{E_{l}}\;E_{l}$, describing the effort of maintaining the enzymes. The burdens $h_{E_{l}}$ of different enzymes represent, e.g., differences in molecular mass, post-translation modifications, enzyme maintenance, overhead costs for ribosomes, as well as effects of misfolding and non-specific catalysis. The enzyme burdens $h_{E_{l}}$ can be chosen heuristically, for example, depending on enzyme sizes, amino acid composition, and lifetimes (see S1 Text section 2.1). Setting $h_{E_{l}}\;=\;m_{l}$ (protein mass in Daltons), *q* will be in mg protein per liter. Considering the specific amino acid composition of enzymes, we can also assign specific costs to the different amino acids. Alternatively, an empirical cost per protein molecule can be established by the level of growth impairment that an artificial induction of protein would cause [44, 45]. Thus, each reaction flux *v*_*l*_ is associated with an enzyme cost *q*_*l*_, which can be written as a function $q_{l}(c,\;v_{l})\equiv h_{E_{l}}\;E_{l}(c,\;v_{l})\;$ of flux and metabolite concentrations. From now on, we refer to log-scale metabolite concentrations *x*_*i*_ = ln *c*_*i*_ in order to obtain simple optimality problems below. From the separable rate law Eq 5, we obtain the enzyme cost function

$$q\left(x,v\right)\equiv \sum_{l}h_{E_{l}}\;E_{l}\left(x,v_{l}\right)=\sum_{l}h_{E_{l}}\cdot v_{l}\cdot \frac{1}{k_{\text{cat},l}^{+}}\cdot \frac{1}{\eta_{l}^{\text{rev}}\left(x\right)}\cdot \frac{1}{\eta_{l}^{\text{sat}}\left(x\right)}\cdot \frac{1}{\eta^{\text{reg}}\left(x\right)}$$ (6)

for a given pathway flux **v**. If the fluxes are fixed and given, our enzyme cost becomes, at least formally, a function of the metabolite levels. We call it *enzyme-based metabolic cost* (EMC) to emphasize this fact. The cost function is defined on the metabolite polytope P, a convex polytope in log-concentration space containing the feasible metabolite profiles. Like the triangle in Fig 2, the polytope is defined by physiological and thermodynamic constraints. It can be bounded by two types of faces: On “E-faces”, one reaction is in equilibrium, and enzyme cost goes to infinity; “P-faces” stem from physiological metabolite bounds. The shape of the cost function depends on rate laws, rate constants, and enzyme burdens, and its minimum points can be inside the polytope or on a P-face (see Fig 2f).

### Enzyme cost minimization

The cost function *q*(**x**, **v**) reflects a trade-off between fluxes to be realized and enzyme expression to be minimized, where the relation between fluxes and enzyme levels is not fixed, but depends on metabolite log-concentrations **x**. Wherever trade-offs exists in biology, it is common to assume that evolution converges to Pareto-optimal solutions [1], i.e. cases where there are no other solution with both a higher flux and a lower cost. Therefore, we can now use this principle to predict metabolite and enzyme concentrations in cells. As with our simple model in Fig 1, the metabolite profile that minimizes the enzyme cost for a given flux, and the corresponding enzyme profile (computed using Eq 5) could be good predictions for the abundance of metabolites and enzymes in naturally evolved organisms.

The resulting method, which we call *enzyme cost minimization* (ECM), is a convex optimization problem and can be solved with local optimizers. Enzyme demand and enzyme cost functions, for single reactions or pathways, are differentiable, convex functions on the metabolite polytope. This convexity holds for a variety of rate laws, including rate laws describing polymerization reactions [46], and even for the more complicated problem of preemptive enzyme expression, i.e., a cost-optimal choice of enzyme levels that allows the cell to deal with a number of future conditions (see S1 Text section 3.7). If a model contains non-enzymatic reactions, this changes the shape of the metabolite polytope, but not the enzyme cost function, and the polytope remains convex, e.g., if the non-enzymatic reactions are irreversible with mass-action rate laws (see Methods). Obviously, metabolite and enzyme levels may be subject to various other constraints that are not reflected by our pathway model. To assess how easily the metabolic state can be adapted to external requirements, we can study the cost of deviations from the optimal metabolite levels. If the cost function *q*(**x**) has a broad optimum as in Fig 2, cells may flexibly realize metabolite profiles around the optimal point, and the choice of metabolite levels may vary from cell to cell. We can quantify the tolerable variations by relaxing the optimality assumptions and computing a tolerance range for each metabolite level (see Methods). To apply ECM in practice, we developed a workflow in which a kinetic model is constructed, all necessary enzyme parameters are determined by a method called parameter balancing, and optimal metabolite and enzyme levels are predicted along with their tolerance ranges. In parameter balancing [47, 48], a complete, consistent set of enzyme parameters is determined from measured values by employing prior distributions, parameter dependencies arising from thermodynamic laws, and Bayesian statistics (for details, see Methods). Different kinds of EMC functions and constraints (e.g., defining concentration ranges for specific metabolites) can be chosen. Missing data (e.g., *K*_M_ values), can thus be handled in two ways: either, by using a simplified EMC function that does not require this parameter, or by relying on parameter values chosen by the workflow.

### Which factors shape the optimal enzyme profile and how?

Beyond minimizing the total enzyme cost, one can also use ECM to analyze the individual enzyme demands. When the metabolite levels are known, the demand can be directly calculated and each efficiency factor in Eq (6) reflects a different part of the cost (see Methods). Alternatively, by omitting some factors or replacing them with constant numbers 0 < *η* ≤ 1, simplified enzyme cost functions with fewer parameters can be obtained. For example, *η*^rev^ = 1 would imply an infinite driving force Θ → ∞ and a vanishing backward flux, *η*^kin^ = 1 implies full substrate saturation, as well as full allosteric activation and no allosteric inhibition (or no allosteric regulation at all). In these limiting cases, enzyme activity will not be reduced, and enzyme demand will be given by the capacity-based estimate $v/k_{\text{cat}}^{+}$, a lower bound on the actual demand. Such simplifications are practical if rate constants are unknown.

Depending on the data available (e.g., *k*_cat_ values, equilibrium constants, or even *K*_M_ values), one may choose between different cost functions with different data requirements: EMC0 (“sum-of-fluxes-based” same prefactors for all enzymes), EMC1 (“capacity-based”, setting all *η* = 1 and thus replacing reaction rates by the maximal velocities), EMC2 (“reversibility-based”; considering driving forces, and setting *η*^kin^ = 1), EMC3 (“saturation-based”, assuming simple rate laws depending on products of substrate or product concentrations, and including the driving forces), and EMC4 functions (“kinetics-based”; with dependence on individual metabolite levels). Details of the simplified EMC functions are given in Table 2 and Table A in S1 Text. Each EMC function is a lower bound on the subsequent functions; i.e., even if only a simplified cost function can be used, it will always yield a lower bound on the cost computed using the full EMC4 model.

**Table 2:** Simplified enzyme cost functions. By omitting some terms in Eq (5), we obtain a number of cost functions with simple dependencies on enzyme parameters and metabolite levels. Terms marked by ✓ appear explicitly in the rate and cost formulae, while other terms are omitted or set to constant values. The EMC0 function yields the sum of fluxes, EMC1 functions contain enzyme-specific flux burdens based on *k*_cat_ and *h* values (i.e., replacing reaction rates by their maximal velocities). EMC2 depends on metabolite levels only via the driving forces. EMC3 functions are based on simplified rate laws, and EMC4 functions capture all rate laws, possibly including allosteric regulation. The rate law denominators *D*^S^, *D*^SP^, *D*^1S^, and *D*^1SP^, and the EMC functions themselves are described in Table A in S1 Text.

EMC function	*η*^rev^(Θ(c))	*η*^kin^(c)	Parameters	Denominators	Depends on
EMC0 (“Sum of fluxes”)	-	-	-		
EMC1 (“Capacity-based”)	-	-	*h*_E_, $k_{\text{cat}}^{+}$		
EMC2 (“Reversibility-based”)	✓	-	*h*_E_, $k_{\text{cat}}^{+}$, *K*_eq_	*D*^S^, *D*^SP^	Driving force
EMC3 (“Saturation-based”)	✓	✓	*h*_E_, $k_{\text{cat}}^{+}$, *K*_eq_, *K*_M_	*D*^1S^, *D*^1SP^	Metabolite levels
EMC4 (“Kinetics-based”)	✓	✓	*h*_E_, $k_{\text{cat}}^{+}$, *K*_eq_, *K*_M_	general	Metabolite levels

Let us consider the various simplifications in more detail. If fluxes are the only data available, we may assign identical catalytic constants and burdens to all enzymes and assume that all reactions run at their maximal velocities. Then, enzyme levels and fluxes will be proportional for all reactions, the cost function in Eq (6) will be EMC0, and the cost will be proportional to the sum of fluxes. However, catalytic constants span many orders of magnitude [25], as do molecular masses of enzymes, suggesting that EMC0 is an oversimplification. If individual $k_{\text{cat}}^{+}$ and $h_{E_{l}}$ values are known, we can define an individual flux burden $a_{v_{l}}^{cat}=h_{E_{l}}/k_{\text{cat}l}^{+}$ for each enzyme, independent of metabolite levels. Then we obtain an EMC1 cost function $\sum_{l}\;a_{v_{l}}^{\text{cat}}\;v_{l}$, which is the same as the cost weights used in FBA with flux minimization [17] or molecular crowding [19]. When *k*_cat_ values are unknown, they can be estimated [42], replaced by “typical” values [25], or bounded by the value $k_{\text{cat}}^{\infty }=10^{8}$ 1/s for a very fast, but diffusion-limited enzyme. The enzyme burdens *h*_E_ can include factors like protein size, protein lifetime, covalent modifications, or space restrictions (see [20] and S1 Text section 2.1).

However, by assuming that enzymes work at their maximal rate and setting *η*^rev^ = *η*^kin^ = 1, we may obtain unrealistic results. First, the simplifying assumption *η*^rev^ = *η*^kin^ = 1 implies uncontrollable metabolic states. In a kinetic model with completely irreversible and substrate-saturated enzymes, the reaction rates would be *independent* of metabolite levels and the steady-state fluxes and metabolite levels would depend on finely tuned enzyme levels [15]. Random variation in enzyme levels would lead to non-steady states, with fast accumulation or depletion of intermediate metabolites. Such states are extremely fragile and thus uncontrollable. When assuming efficiencies *η*^rev^ or *η*^kin^ smaller than 1, we accept an increased cost and thereby acknowledge that control must be paid for by enzyme investments. Second, EMC1 functions underestimate all enzyme costs, and for reactions close to chemical equilibrium the errors may be quite large. For a reaction Gibbs energy of Δ_r_*G*′ = −0.1*RT*, the efficiency of the catalyzing enzyme is reduced by a factor of *η*^rev^ = 1 − e^0.1^ ≈ 0.1, and the demand for enzyme increases by a factor of 1/*η*^rev^ ≈ 10. To account for this decreased efficiency, we can use EMC2 functions, which include the reversibility factor $\eta_{l}^{\text{rev}}=1-{\text{e}}^{-\Theta_{l}\left(x\right)}$. The driving forces are expressed in terms of metabolite log-concentrations Θ_*l*_(**x**) and equilibrium constants, which need to be known. This factor approaches infinity as reactions reach equilibrium (i.e. where Θ_*l*_ → 0), which is what forces reactions away from equilibrium during cost minimization (see, for example, Fig 2).

The advantage of reversibility-based cost functions (EMC2) is that they are based on *k*_cat_ and equilibrium constants only. Several in-silico methods exist to estimate *K*_eq_ for virtually any biochemical reaction [41, 49] and the values can be easily obtained at http://equilibrator.weizmann.ac.il/ [50]. As in the case of EMC1, *k*_cat_ values can be estimated or set to a default constant value. Methods like MDF [15] and mTOW [23] have been developed to address exactly this situation, where detailed kinetic information is hard to obtain. We discuss the relation between EMC2 and MDF in section 4 of the S1 Text. Aside from the EMC2 function, there are other reversibility-based estimates of the enzyme cost. For instance, the enzyme demand in Fig 2 (an EMC3-function with kinetic constants, fluxes, and enzyme burdens set to 1) has the reversibility-based cost $a_{v}^{\text{pw}}=\sum_{l}{\left[1-{\text{e}}^{-\Theta (c)}\right]}^{-1}$ as a lower bound. Since 1 − *e*^−*x*^ ≤ *x* for all positive *x*, an even lower estimate is ∑_*l*_ Θ(**c**)^−1^ (Figure B and Figure C in S1 Text). Some variants of FBA relate fluxes to metabolite profiles, which are then required to be thermodynamically feasible, i.e., within the metabolite polytope. ECM constrains the metabolite profiles even further: as shown in Fig 2, profiles close to an E-face are very costly and can never be optimal. This holds for EMC2 functions and for the more realistic enzyme costs, which will even be higher. Thus, regions close to E-faces can be excluded from the polytope. At P-faces, defined by physiological bounds, there will be no such increase, so the optimum may lie on a P-face (see Fig 2f). To exclude regions near E-faces, we simply define lower bounds for all driving forces (see S1 Text section 7.1). These bounds can be used both in ECM or in thermodynamic FBA to reduce the search space.

The next logical step is to relax the assumption that *η*^kin^ = 1. Just like the reversibility factor *η*^rev^, the kinetic factors *η*^sat^ and *η*^reg^ can be used to define tighter constraints on metabolite levels. However, unlike *η*^rev^, the kinetic terms may take various forms and contain many kinetic parameters. To obtain simple, but reasonable formulae in EMC3, we first consider rate laws in which enzyme molecules exist only in three possible states: unbound, bound to all substrate molecules, or bound to all product molecules. Metabolites affect the rate only through the mass-action terms *S* = ∏_*i*_(*s*_*i*_/*K*_M__*i*_) (for substrates) and *P* = ∏_*j*_ *p*_*i*_/*K*_M__*j*_ (for products), and the degree of saturation is determined by *η*^sat^ = *S*/(1 + *S* + *P*), where the formula effectively has two Michaelis-Menten constants: one for substrates and one for products (which are equivalent to the product of all *K*_M__*i*_ and all *K*_M__*j*_ values). EMC3 represents a balance between complexity and requirement for kinetic parameters, and is a practical cost function if simple, realistic rate laws are desired. The EMC4 functions, finally, represent general rate laws and *η*^kin^ can take many different forms depending on mechanism and order of enzyme-substrate binding. Again, for simplicity, we resort to analyzing only a small set of relatively general templates for EMC4, known as convenience kinetics [51] or modular rate laws [21]. Nevertheless, our formalism allows a much wider range of rate laws, and we consider EMC4 a wild-card cost function that covers almost any reasonable rate law (see S1 Text section 2.2 for more details).

### Enzyme and metabolite levels in *E. coli* central metabolism

**Fig 3:**
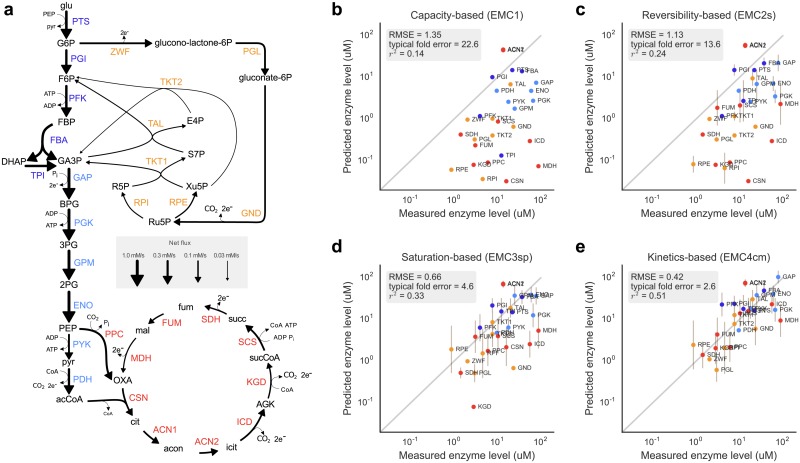
Predicted enzyme levels in *E. coli* central metabolism. (a) Network model with pathways marked by colors. Flux magnitudes are represented by the arrows’ thickness. (b) The ratio flux/$k_{\text{cat}}^{+}$ (EMC1) as a predictor for enzyme levels. Points on the dashed line would represent precise predictions. (c) Enzyme levels predicted by the reversibility-based EMC2(S) function. Vertical bars indicate tolerance ranges obtained from a relaxed optimality condition (allowing for a one percent increase in total enzyme cost). (d) Enzyme levels predicted with EMC3 function representing fast substrate or product binding. (e) Enzyme levels predicted with EMC4 function based on the common modular rate law [21]. In all sub-figures (b-e), RMSE is the root mean squared error (in log_10_-scale) of our predictions compared to the measured enzyme levels, and *r* stands for the Pearson correlation coefficient. Predictions are based on fluxes from [52], $k_{\text{cat}}^{+}$ and *K*_M_ values from BRENDA [40], and compared to protein data from [53]. For metabolite predictions, see Figure E in S1 Text.

To benchmark our optimality-based prediction of metabolite, we applied ECM to a model of *E. coli* central metabolism, containing three major pathways: glycolysis, the pentose phosphate pathway, and the TCA cycle (see Fig 3a, and Methods for modeling details). Fig 3b–3d compares predicted enzyme profiles to measured protein levels [53]. The absolute values of predicted enzyme levels arise directly from the model, using the fluxes reported in [52], while cellular protein concentrations were obtained from proteomics data (measured in similar conditions [53]) and assuming an average cell volume of ~ 1 fL (10^−15^ liters) [54]. EMC4 predicts values that are of the right order of magnitude and reflect differences in enzyme levels along the pathways. The prediction error of 0.42 for enzyme levels (RMSE: root mean square error on a log_10_-scale) corresponds to a typical fold error of 10^RMSE^ = 2.6. In line with the measured protein levels, the predicted enzyme levels tend to be larger in glycolysis than in TCA and pentose phosphate pathway, reflecting the larger fluxes and less-favorable thermodynamics. All predictions including metabolite concentrations, thermodynamic forces and *c*/*K*_M_ ratios can be found online at the accompanying website www.metabolic-economics.de/enzyme-cost-minimization/.

**Fig 4:**
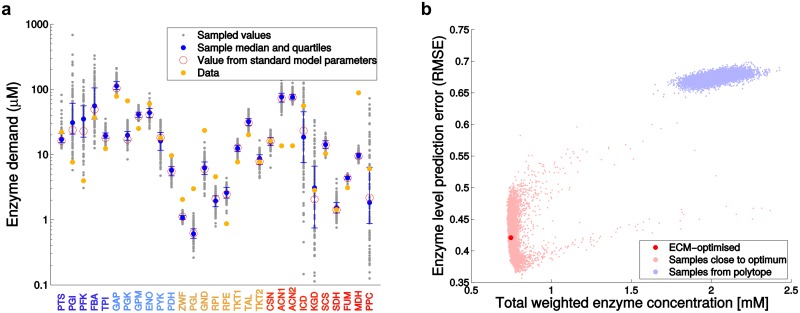
Prediction uncertainties and evidence for cost optimality. (a) Uncertainty of predicted enzyme levels due to uncertain model parameters. A hundred sets of kinetic model parameters were generated by Monte Carlo sampling. Due to the multivariate distribution used for sampling, each parameter set satisfies the Haldane relationships. At the same time, fluxes were sampled according to their experimental error bars (typically around 15% of the measured flux), and the fixed metabolite concentrations were randomly varied in a ± 5% range. The resulting predicted enzyme levels, computed using the EMC4cm score, are shown by small gray dots. Solid blue circles show medians, and error bars show 25% and 75% quantiles; empty red circles show the original ECM4cm prediction, i.e. without sampling. (b) The enzyme levels in *E. coli* appear to be cost-optimized. We compared the ECM solution (with ECM4cm score) to enzyme profiles obtained from metabolite profiles randomly sampled in the metabolite polytope. The ECM solution (red) or metabolite profiles sampled in a close neighborhood (pink) yield significantly better enzyme predictions (quantified by RMSE, compare Fig 3) than metabolite profiles sampled in the entire polytope (light blue). The total enzyme cost (on x-axis) represents the sum of weighted enzyme concentrations (in mM); the weight of an enzyme is given by its amino chain length, divided by the median chain length of all enzymes considered.

We note that predicted enzyme levels become more accurate as more complex cost functions are used, with a prediction error decreasing monotonically from 1.35 with EMC0 to 0.42 with EMC4. The capacity-based enzyme cost (EMC1) assumes that enzymes operate at full capacity ($v=E\;k_{\text{cat}}^{+}$) and therefore underestimates all enzyme levels (Fig 3b). In reality, many reactions in central metabolism are reversible and many substrates do not reach saturating concentrations. When taking these effects into account, predictions come closer to measured enzyme levels (Fig 3c–3e). For instance, FUM (fumarase, fumA) and MDH (malate dehydrogenase) have a much higher predicted level in EMC2-4 than in EMC1 as the reversibility-based costs account for their low driving forces. Similarly, the predicted levels of two pentose-phosphate enzymes (ribulose-5-phosphate epimerase RPE and ribose phosphate isomerase RPI) are much higher in EMC3 and EMC4 because of their low affinity for the substrate ribulose-5-phosphate (Ru5P). In some cases, however, the more complex EMC4 fails to improve the prediction over the simpler methods. For instance, the 6-phosphogluconolactonase (PGL) and phosphoglycerate kinase (PGK) reactions are underestimated by all EMC functions, perhaps due to regulation mechanisms that reduce activity such as allosteric inhibition. In very few cases, EMC4 overestimates the level of an enzyme that has a more precise prediction in EMC1-3, e.g. phosphofructokinase (PFK). Overall, the EMC4 function performs substantially better on average than the simpler cost functions even though it relies on a larger set of parameters, many of which are known with low certainty. Moreover, EMC4 predicts well the total of all enzyme levels (0.64 mM, compared to the measured value—0.62 mM), while the other EMC function underestimate this value (0.17, 0.24 and 0.43 mM for EMC1, EMC2 and EMC3 respectively). To test the sensitivity of our results to the choice of parameters, we performed random sampling of kinetic constants, fluxes and fixed metabolite levels, and analyzed the effect on the enzyme level predictions (see Methods and Fig 4a). We further tested the sensitivity to our choice of proteomic data, by repeating the entire analysis using measured enzyme concentrations from [55] and reached essentially the same findings (see Figure F in S1 Text).

Finally, we tested whether our kinetic model can also predict enzyme levels without the assumption of cost optimality: to do so, we randomly sampled feasible metabolite profiles from the metabolite polytope, computed the resulting enzyme profiles, and compared them to proteomic data. It turned out that the cost-optimal metabolite profile, or similar profiles, yielded significantly better predictions than metabolite profiles sampled from a broader range (see Methods and Fig 4b). This supports the hypothesis that cost-optimality shapes the metabolic state in *E. coli*.

Although ECM puts enzymes on a pedestal due to their relatively high cost, the metabolite concentrations are key to minimizing that cost. One would thus expect to find good correspondence between the predicted metabolite profile and concentrations measured *in vivo*, especially when predictions of enzyme levels are good. Since some EMC functions leave metabolite levels underdetermined, we penalized very high or low metabolite concentrations by adding a second, concentration-dependent objective to the optimization problem. In particular for EMC0 and EMC1, this regularization term is the only term—aside from global constraints—that determines the metabolite concentrations as they do not affect enzyme cost whatsoever. In all other cases, the term mostly influences metabolites that have a minimal effect on the cost. Comparing the EMC metabolite prediction with in-vivo experimental data, as shown in Figure E in S1 Text, the predicted metabolite levels are in the correct scale. Similar to enzyme level predictions, EMC4cm has the smallest prediction error—about 0.62 (corresponding to a typical fold error of 4.1).

**Fig 5:**
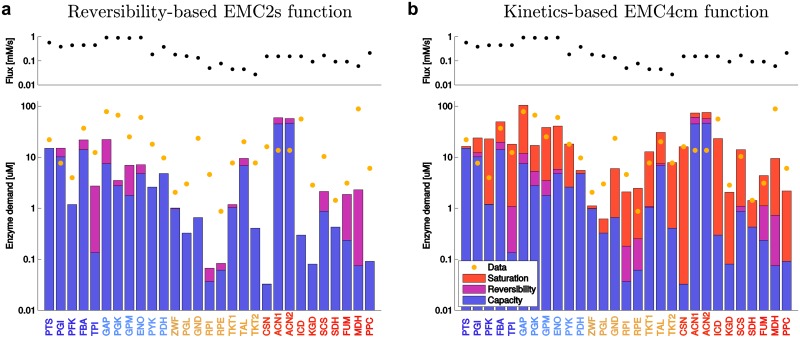
Enzyme demand in central metabolism. (a) Measured fluxes for all reactions (black dots on top) lead to an enzyme demand (bottom). The enzyme demand, predicted by using the reversibility-based EMC2s cost function, can be split into factors representing enzyme capacity and thermodynamics (see Methods). Bars show predicted enzyme levels in mM for individual enzymes on logarithmic scale. Yellow dots denote measured enzyme levels (in μM). Note that the bars do not represent additive costs, but multiplicative cost terms on logarithmic scale; therefore, the relevant feature of the blue bars is not their absolute lengths, but their differences between enzymes. (b) The kinetics-based EMC4cm cost function includes saturation terms and yields more accurate predictions. Starting from the capacity cost (in blue), the reversibility (purple) and saturation (red) terms increase the enzyme demands and decrease the variability between enzymes (on log-scale). Note that flux data (circles) and protein data (yellow dots) are identical in both plots.

We can now use EMC analysis to rationalize cellular enzyme levels. Fig 5 (like the scheme in Fig 1b) shows the specific contributions to enzyme demand for each reaction. The reversibility cost terms provided by EMC2s (purple bars in Fig 5a) improve the enzyme demand predictions in most cases, compared to the basic capacity-based costs. However, the EMC4cm predictions show that saturation-based costs (orange bars in Fig 5b) are often larger than the reversibility costs, and they improve the predictions even more. For practical cost estimates, for example when computing flux burdens for FBA, we can conclude that multiplying the experimentally determined *k*_cat_ values by reversibility factors will likely improve the fidelity of FBA predictions. For more details, see S1 Text section 4.

## Discussion

When applying mathematical models to learn about biology, one typically faces a conflict between model accuracy and the amount of available data. Metabolic systems are known to abide to several physical and physiological considerations, all of which are mathematically well-described (e.g. flux balance, thermodynamics, kinetics, and cost-benefit optimality). Taking all of these aspects into account would create very detailed models but at the price of considerably increasing the demand for data. Here, we obtained a flexible modeling method by combining the two main modeling approaches, constraint-based and kinetic modeling, in a new way: with fixed metabolic fluxes, kinetic models are used to determine a cost-optimal state. The tiered approach in ECM allows for different levels of detail, which can easily be matched to the amount of existing data. The minimal requirement for running ECM is to have a metabolic network with given steady-state fluxes, while the maximal requirement would be a fully parameterized kinetic model. The method applies to individual metabolic pathways and, theoretically, entire metabolic networks. No matter if we model exponentially growing cells, microbial cells in stationary phase, or non-growing eukaryotic cells, the sum of enzyme costs per unit flux is a meaningful objective for pathways used by the cell. Although similar approaches exist in dynamic modeling [48, 56] and enzyme optimization [4, 15, 23], ECM extends these ideas to the most general kinetic rate laws and cost functions, while proving that the emerging optimization problem is convex and thus easily (albeit numerically) solvable. ECM advances metabolic modeling in six different ways:

**1. Solving the enzyme optimality problem in metabolite space** One way of modeling the cost and benefit of enzymes is to study kinetic models and to treat enzyme levels as free variables to be optimized. However, this calculation can be hard because enzyme profiles may lead to one, several, or no steady states, and the resulting optimality problem can be non-convex. By fixing fluxes and using metabolite concentrations as our primary variables, we drastically simplify this optimization problem. Flux directions and the second law of thermodynamics impose constraints that define a set of feasible metabolite profiles, the metabolite polytope. This polytope is used here as a space for screening, sampling, and optimizing metabolic states; accordingly bounds on metabolite concentrations or driving forces can be easily formulated as linear constraints. Using log-concentrations as free variables, and given a (steady and non-steady) flux distribution, we can parametrize the set of metabolic states very easily: we simply consider all feasible metabolite profiles and compute, for each of them, the corresponding enzyme profile by taking the inverse rate laws. With enzyme levels as free variables, parameterizing the set of metabolic states would be much more complicated.

**2. Convexity** The metabolite polytope not only provides a good search space, but it also facilitates optimization because enzyme cost is a convex function of the metabolite log-concentrations (see S1 Text section 3.2). Convexity makes the optimization tractable and scalable—unlike a direct optimization in enzyme space. Simple convexity holds for a wide range of rate laws and for extended versions of the problem, e.g., including bounds on the sum of (non-logarithmic) metabolite levels or bounds on weighted sums of enzyme fractions. By using specific rate laws (e.g., the ECM4cm rate law, as shown by our colleague Joost Hulshof—personal communication) or by adding a regularization term, representing additional biological objectives, we can even ensure strict convexity, and thus the existence of a unique optimum that can be efficiently found. It is important to distinguish this computational scalability, which is facilitated by convexity, from other pragmatic issues that arise when increasing the scale of a model, in particular the scarcity of kinetic data. Standard kinetic modeling is difficult to apply to whole-cell metabolic networks due to both scalability problems. Therefore, even if network-wide *k*_cat_ and *K*_M_ values were to become available (e.g. by estimation methods that rely on high-throughput data [42]), it would still be impractical to exhaustively search the parameter space. ECM—due to its convexity—is solvable even on a genomic scale.

**3. Separable rate laws disentangle individual enzyme cost effects** To assess how different physical factors shape metabolic states, we focused on separable rate laws, which lead to a series of easily interpretable, convex cost functions. The terms in these functions represent specific physical factors and require different kinetic and thermodynamic data for their calculation. By neglecting some of the terms, one obtains different approximations of the true enzyme cost. The more terms are considered, the more precise our predictions about metabolic states becomes (see Methods and S1 Text section 2). By comparing the different scores, we can estimate the enzyme cost that cells “pay” for running reactions at small driving forces (to save Gibbs free energy) or for keeping enzymes beneath substrate-saturation (e.g., to dampen fluctuations in metabolite levels). Of course, it is often important to keep models simple and the number of parameters small, and therefore the stripped-down versions of ECM can be useful in practice. For example, in some conditions such as batch-fed *E. coli*, a simple enzyme economy might still be a realistic approximation. Our results in Fig 3 indicate that indeed one can predict enzyme levels quite well even with relatively simple enzyme cost objectives. Finally, in conditions where ECM’s predictions are far from the measured enzyme levels, we can focus on specific enzymes or pathways that deviate the most, which may therefore display optimization or adaptations beyond simple resource allocation.

**4. Relationship to other optimality approaches** Beyond the practical advantages of using factorized enzyme cost functions, they also allow us to easily compare our methods to earlier approaches. These approaches typically focused on only one or two of the factors that are taken into account in ECM, and many of them can be reformulated as approximations of ECM (as we have shown for MDF [15] and, by proxy, earlier thermodynamic profiling methods [57, 58]). For example, the optimization performed by FBA with flux minimization is equivalent to using EMC0, while EMC1 is based on the same principles as FBA with molecular crowding [19], pathway specific activities [2], and Constrained Allocation Flux Balance Analysis (CAFBA) [59]. Thermodynamic profiling methods [15, 57, 58] which use driving forces as a proxy for the cost, can be compared to EMC2 (where all *k*_cat_ are assumed to be equal, see S1 Text section 4). To our knowledge, ECM is the first method that accounts for substrate and product saturation (as well as allosteric) effects in the optimization process and guarantees a convex (i.e., relatively tractable) optimality problem. Moreover, ECM highlights how different aspects of metabolism are linked: most importantly, thermodynamic feasibility [15] is generalized by the quantitative notion of thermodynamic efficiency, which then turns out to be a natural precondition for enzyme economy.

**5. Kinetics-based flux cost functions for flux balance analysis** Accordingly, results from ECM can be used to improve flux analysis [13, 23] by defining more realistic flux cost functions for FBA and by providing formulae for the pathway specific activity [2] (see S1 Text section 2.3). In practice, the cost weights used in FBA so far (typically, defined by *k*_cat_ values and enzyme sizes) could be adjusted by dividing them by efficiency factors obtained from our workflow. In FBA (specifically in variants with flux minimization or molecular crowding), flux cost or enzyme demand are linear functions of the fluxes. Enzyme Cost Minimization allows us to compute plausible prefactors for this formula from detailed knowledge of enzyme kinetics: by rearranging Eq (6), we can write the enzyme cost as a linear function $q\;=\;\sum_{l}\;a_{v_{l}}\;\cdot \;v_{l}$ with flux burdens $a_{v_{l}}\left(c\right)=h_{E_{l}}\cdot \frac{1}{k_{\text{cat},l}^{+}}\cdot \frac{1}{\eta_{l}^{\text{rev}}\left(c\right)}\cdot \frac{1}{\eta_{l}^{\text{sat}}\left(c\right)}\cdot \frac{1}{\eta^{\text{reg}}\left(c\right)}$. The flux burden has a lower bound $a_{v_{l}}^{\text{cat}}=h_{E_{l}}/k_{\text{cat},l}^{+}$, denoting the cost per flux under ideal conditions. Ignoring all dependencies on metabolite levels, $a_{v_{l}}^{\text{cat}}$ could be used as a cost weight to define flux cost functions for FBA. However, these values are further increased by the reciprocal values of the enzyme efficiency factors. A similar, flux-specific enzyme cost (or, inversely, a flux per enzyme invested) can also be defined for entire pathways. The Pathway Specific Activity (PSA) [2] is defined as the flux per enzyme mass (in units of mmol/s per mg of enzyme) and can be computed by treating enzyme mass as a cost function. Assuming that *η*^rev^ = *η*^kin^ = 1 and that cost is expressed in terms of protein mass in Daltons $\left(h_{E_{l}}\;=\;m_{l}\right)$, we obtain the pathway specific activity using the formula *A*_pw_ = *v*_pw_/*q*.

**6. Embedding ECM into flux analysis** Furthermore, ECM could be “embedded” into FBA by screening a finite set of possible flux distributions, characterizing each of them by quantitative cost (using ECM) and choosing the most cost-favorable mode. Since we now know that any metabolic state that has maximal specific rate is an elementary flux mode [60], it would be sufficient to scan only the elementary flux modes. This could be seen as a version of minimal-flux FBA, but one that uses kinetic knowledge instead of the various heuristic assumptions that go into FBA. Second, we can derive realistic bounds on thermodynamic forces based on kinetics and enzyme cost, or lower/upper bounds on substrates/products concentrations to avoid extreme saturation effects. All these constraints follow systematically from setting upper limits on the individual efficiency factors. By applying them in thermodynamics-based flux analysis, we shrink the metabolite polytope by excluding strips at its boundary where costs would be too high to allow for an optimal state. Similarly, by giving individual weights to thermodynamic driving forces, MDF could be used as a method to optimize some lower bound on the system’s enzyme cost (see S1 Text section 4).

ECM is based on the central assumptions that the metabolic states of cells are cost-optimized and that cost arises from cellular protein levels. Both assumptions are of course debatable. There is ample evidence that cells assume apparently sub-optimal states in order to maintain robust homeostasis or to gain metabolic flexibility for addressing future challenges [1]. For example, an allosterically regulated enzyme will often not reach its maximal possible activity, so investment in enzyme production appears to be wasted. Nevertheless, cells pay this price in order to gain the ability to adjust quickly to changes (i.e. within seconds rather than the minutes required for altering gene expression). One intriguing example is the bacterium *Lactococcus lactis*, which uses the exact same enzyme expression profile for completely different anaerobic growth modes [61]: slow growth / high yield acetate fermentation, and fast growth / low yield lactate fermentation. The reason that low-yield strategies achieve higher growth rates is typically attributed to much lower protein investments, but obviously, this is not the case in the *Lactococcus lactis* experiments. This stands in contrast to aerobic fermentation in *E. coli*, which seems to be explained well by predictable shifts in protein allocation [5].

As these examples show us, the importance that certain cells attribute to saving on protein costs is highly variable and, in some cases, can be negligible: for instance, when protein levels are already low or when protein demands change quickly and unpredictably. Moreover, random fluctuations in protein levels will be tolerable as long as the impact on fitness is not very high. Nevertheless, we think that a simple principle of cost optimality as in ECM can be a useful heuristics. On the one hand, it can reveal the *minimal protein investment* that would be required to support a certain metabolic state. In metabolic engineering, such predicted investments may be used to rule out potential, but uneconomical metabolic pathways. On the other hand, ECM can be used as a background model to be compared to more complicated optimality-based cell models. Such comparisons can allow us to quantify the impact of other fitness objectives in units of “protein cost”, to learn which objectives can best explain cellular behavior, and to describe non-optimality as a deviation from a presumable cost-optimal state.

Furthermore, ECM can be extended to cover more realistic optimality scenarios. Some alternative objectives can be integrated into ECM by adding them to the objective function. We have tried to keep our method as general as possible to facilitate such objectives, e.g. by allowing for non-linear, convex enzyme costs (*h*(*E*)). In particular, metabolite levels may be under additional constraints or optimality pressures because they appear in pathways outside our model, which may favor high or low levels of the metabolites. Also chemical molecule properties, such as hydrophobicity or charge, may affect the preferable metabolite levels in cells [62]. For example, if our model captures an ATP-producing pathway, low ATP levels will be energetically favorable, whereas other ATP-consuming pathways would favor higher ATP levels. To account for this trade-off, a requirement for sufficiently high ATP levels can be included in our ECM model by constraints or additional objectives *b*^(c)^(**x**) that penalize low ATP levels (see Methods). If metabolite levels are kept far from their upper or lower physiological bounds, this will allow for more flexible adjustments in case of perturbation.

If enzyme profiles were shaped by optimal resource allocation, as assumed in ECM, this would have consequences for the shapes of enzyme and metabolite profiles. Enzyme cost, thermodynamic forces, and an avoidance of low substrate levels would be tightly entangled, and the shapes of enzyme profiles would reflect the role of enzymes in metabolism, i.e., the way in which they control metabolic concentrations and fluxes. Among other things, this would imply three general properties of enzyme profiles:

**1. Enzyme cost is related to thermodynamics** In FBA, thermodynamic constraints and flux costs appear as completely unrelated aspects of metabolism. Thermodynamics is used to restrict flux directions, and to relate them to metabolite bounds, while flux costs are used to suppress unnecessary fluxes. In ECM, thermodynamics and flux cost appear as two sides of a coin. Like in FBA, flux profiles are thermodynamically *feasible* if they lead to a finite-sized metabolite polytope, allowing for positive forces in all reactions. However, the values of these forces also play a role in shaping the enzyme cost function on that polytope. Together, metabolite polytope and enzyme cost function (as in Fig 2) summarize all relevant information about flux cost.

**2. Enzyme profiles reflect local metabolic necessities** What are the factors that determine the levels of specific enzymes? High levels are required whenever catalytic constants, driving forces, or substrate concentrations are low. Accordingly, an efficient use of enzymes requires metabolite profiles with sufficient driving forces (for energetic efficiency) and sufficient substrate levels (for saturation efficiency). Trade-offs between these requirements, together with predefined bounds, will shape the optimal metabolite profiles [23]: in a linear pathway, a need for energetic efficiency will push substrate concentrations up and product concentrations down; the need for saturation efficiency has the same effect. However, since the product of one reaction is the substrate of another reaction, there will be trade-offs between efficiencies in different reactions. Therefore, where enzymes are costly or show low *k*_cat_ values, we may expect a strong pressure on sufficient driving forces and substrate levels.

**3. Enzyme profiles reflect global effects of enzyme usage** If enzyme profiles follow a cost-benefit principle, costly enzymes should provide large benefits. Such a correspondence has been predicted, for example, from kinetic models in which flux is maximized at a fixed total enzyme investment [63]: in optimal states, high-abundance enzymes exert a strong control on the flux, and enzymes with strong flux control are highly abundant. If this applies in reality, then high investment (e.g., large enzyme levels shown in Fig 1A) could be seen as a sign of large benefit, in terms of flux control. Here, we studied a different optimality problem (fixing the fluxes and optimizing enzyme levels under constraints on metabolite levels), and obtain a more general result. The optimal enzyme cost profile obtained by ECM is a linear combination of flux control coefficients and, possibly, control coefficients on metabolites that hit upper or lower bounds (see S1 Text section 7.4). In simple cases (e.g., the example in Fig 2), where there is only one flux mode and none of the metabolites hits a bound, enzyme demands and flux control coefficients will be directly proportional.

Beyond the analysis of central metabolism, ECM can be applied to select candidate pathways in metabolic engineering projects. A prediction of enzyme demands or specific activities (S1 Text section 2.3) can be helpful at different stages of pathway design. The optimal expression profile for a pathway can be determined, critical steps in a pathway can be detected (i.e., steps where lowering the enzyme’s flux-specific cost $a_{v_{l}}$ would be most important), and enzyme demand and cost can be compared between pathway structures. This type of application is not unique to ECM, and although several of the methods that we mention throughout this manuscript [2, 4, 23, 32, 64, 65] have been used for this purpose in the past, we believe that ECM manages to bring them all under one umbrella.

## Materials and Methods

### Metabolite polytope and enzyme cost functions

A metabolic network with given flux directions, equilibrium constants, and metabolite bounds defines the *metabolite polytope*. This convex polytope P in the space of log-concentrations *x*_*i*_ = ln *c*_*i*_ represents the set of feasible metabolite profiles. The flux profile used can be stationary (e.g. determined by FBA or ^13^C MFA) or non-stationary (e.g. from dynamic ^13^C labeling experiments [66]). If the provided flux directions are thermodynamically infeasible, the metabolite polytope will be an empty set, P=∅. The faces of the metabolite polytope arise from two types of inequality constraints. First, the physical ranges $x_{i}^{\text{min}}\leq x_{i}\leq x_{i}^{\text{max}}$ of metabolite levels define a box-shaped polytope (bounded by P-faces). Some metabolite levels may even be constrained to fixed values. Second, each reaction must dissipate Gibbs free energy, and to make this possible, driving forces and fluxes must have the same signs (Θ_*l*_ ⋅ *v*_*l*_ > 0), and thus $\text{sign}\left(v_{l}\right)=\text{sign}\left(\Delta_{r}{G^{\prime }}_{l}^{\circ }/RT+\sum_{i}n_{il}x_{i}\right)$. The resulting constraints define E-faces of the metabolite polytope (representing equilibrium states, Θ_*l*_ = 0). Close to these faces, enzyme cost goes to infinity.

### Separable rate laws and enzyme cost functions

**Fig 6:**
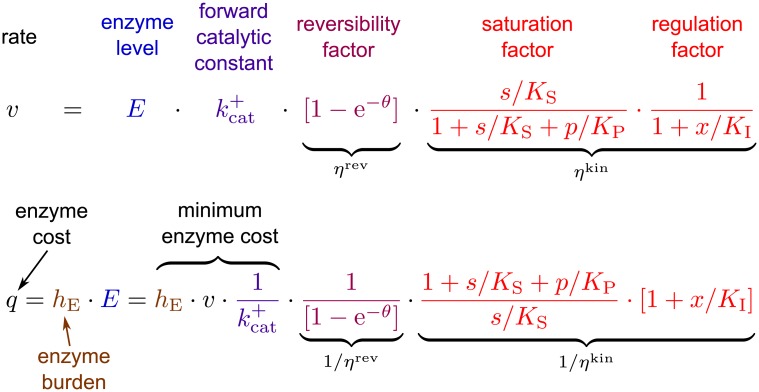
Rate law and enzyme demand of reversible Meichalis-Menten reactions. For a reaction S ⇌ P with reversible Michaelis-Menten kinetics, a driving force *θ* = −Δ_r_*G*′/*RT*, and a prefactor for non-competitive allosteric inhibition, the rate law can be written as with inhibitor concentration *x*. In the example, with non-competitive allosteric inhibition, the kinetic factor *η*^kin^ could even be split into a product *η*^sat^ ⋅ *η*^reg^. The first two terms in our example, $E\cdot k_{\text{cat}}^{+}$, represent the maximal velocity (the rate at full substrate-saturation, no backward flux, full allosteric activation), while the following factors decrease this velocity for different reasons: the factor *η*^rev^ describes a decrease due to backward fluxes (see Figure A in S1 Text) and the factor *η*^kin^ describes a further decrease due to incomplete substrate saturation and allosteric regulation (see Fig 1b). The inverse of all these terms appear in the equation for enzyme demand, *q*, which is given by the enzyme level multiplied by the burden of that enzyme, *h*_E_.

According to Eq (3), reversible rate laws can be factorized into four terms: the enzyme level *E*, its forward catalytic constants $k_{\text{cat}}^{+}$, and two efficiency factors [22]. In Fig 6 we add a non-competitive allosteric inhibitor *x*. While the enzyme level and $k_{\text{cat}}^{+}$ are not directly affected by the concentration of metabolites (although $k_{\text{cat}}^{+}$ can vary with conditions such as pH, ionic strength, or molecular crowding in cells), the efficiency factors *are* concentration-dependent, unitless, and can vary between 0 and 1. The reversibility factor *η*^rev^ depends on the driving force (and thus, indirectly, on metabolite levels), and the equilibrium constant is required for its calculation. The saturation factor *η*^sat^ depends directly on metabolite levels and contains the *K*_M_ values as parameters. Allosteric regulation yields additive or multiplicative terms in the rate law denominator, which in our example can be captured by a separate factor *η*^reg^. In general, *η*^sat^ and *η*^reg^ can be combined into one kinetic factor *η*^kin^, as depicted in Eq 6.

**Fig 7:**
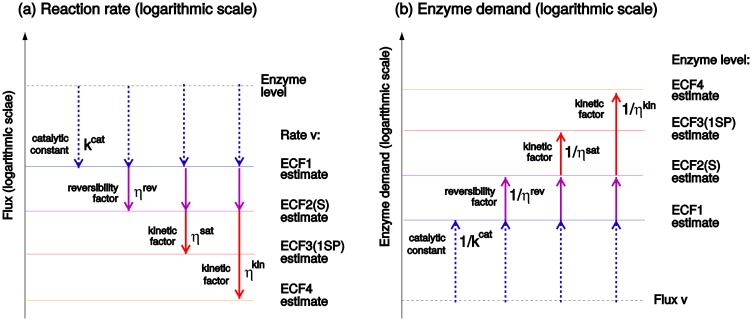
The conversion between fluxes and enzyme levels, in both directions. (a) Starting from the logarithmic enzyme level (dashed line on top), we add the terms $\log\;k_{\text{cat}}^{+}$, log *η*^rev^, and log *η*^kin^, and obtain better and better approximation of the rate. In the example shown, $k_{\text{cat}}^{+}$ has a numerical value smaller than 1. The more precise approximations (with more terms) yield smaller rates. The EMC4 arrows refer to other possible rate laws with additional terms in the denominator. (b) Enzyme demand is shaped by the same factors (see Eq (5)). Starting from a desired flux (bottom line), the predicted demand increases as more terms are considered.

The second equation in Fig 6 describes the enzyme cost for a flux *v*, and contains the terms from the rate law in inverse form multiplied by the enzyme burden *h*_E_. The left-hand part of the equation, $h_{E}\;v/k_{\text{cat}}^{+}$, defines a minimum enzyme cost, which is then increased by the following efficiency factors. Again, 1/*η*^kin^ can be split into 1/*η*^sat^ ⋅ 1/*η*^reg^. By omitting some of these factors, one can construct simplified enzyme cost functions with higher specific rates, or lower enzyme demands (compare Fig 1b). Since both rate and enzyme demand are a product of several terms, it is convenient to depict them as a sum on a logarithmic scale (Fig 7), where the simplified functions are seen as upper/lower bounds on the more complex rate/demand functions.

### Enzyme cost minimization can be formulated as a convex optimality problem for metabolite levels

Enzyme cost minimization (ECM) uses a metabolic network, a flux profile **v**, kinetic rate laws, enzyme burdens, and bounds on metabolite levels to predict optimal metabolite and enzyme concentrations. The enzyme cost of reactions or pathways is a convex function on the metabolite polytope (proof in S1 Text section 3.2), that is, a log-scale metabolite vector **x**, linearly interpolated between vectors **x**_a_ and **x**_b_, cannot have a higher cost than the interpolated cost of **x**_a_ and **x**_b_. Convexity also holds for cost functions *h*(***E***) that are non-linear, but convex over ***E***. Some EMC functions are *strictly* convex (i.e., Eq. (S18) holds with a < sign instead of ≤), while others are not (e.g. EMC2). The most simplified EMC functions are actually constant (as in EMC0 and EMC1). To find an optimal state, we choose an EMC function and minimize the total enzyme cost within the metabolite polytope. Optimal metabolite profiles, enzyme profiles, and enzyme costs are obtained by solving the enzyme cost minimization (ECM) problem

$$\begin{array}{lll} x^{\text{opt}}(v) & = & {\text{argmin}}_{x\in P}\;q(x,v)\\ E^{\text{opt}}(v) & = & E(x^{\text{opt}}(v),v)\\ q^{\text{opt}}(v) & = & q(x^{\text{opt}},v). \end{array}$$ (7)

The total cost *q*(**x**, **v**) (defined in Eq (6)) is the sum of enzyme costs given by EMC functions. Since *q*(**x**) and the metabolite polytope itself are convex, ECM is a convex optimization problem. The optimal enzyme levels depend on external conditions and have to be recalculated after any change in external metabolite levels. There are cases where *q*(**x**) is convex, but not strictly convex, and therefore Eq (7) will have a continuum of optimal metabolite solutions. This holds, in particular, for EMC1 scores, which are independent of metabolite levels, and for EMC2 scores, which only depend on reaction Gibbs free energies, i.e., on some linear combinations of the logarithmic metabolite levels. In such cases, to enforce a unique solution one may add a strictly convex side objective that scores the log-metabolite levels, e.g., a quadratic function favoring metabolite levels close to some typical concentration vector $\hat{x}:{\text{min}}_{x\in P}\;(q\left(x,v\right)+\lambda ||x-\hat{x}\left|\right|)$, where *λ* is a small, heuristically chosen weighing factor (see S1 Text section 3.3). Such extra objectives can be justified biologically, e.g. by assuming that intermediate metabolite levels give cells more flexibility to adapt to perturbations. Strict convexity not only simplifies numerical calculations, but it also guarantees that the optimization problem has a unique solution. In fact, metabolite polytope and cost functions remain convex even under various modifications of the problem. When adding constraints on the total metabolite level, on weighted sums of metabolite levels, or on weighted sums of enzyme levels, the metabolite polytope is intersected with curved manifolds (since we are dealing with concentrations in logarithmic scale) but remains convex (S1 Text section 3.4). Finally, we can consider the more complicated problem of preemptive enzyme expression, where a fixed enzyme profile and allosteric inhibition must allow a cell to realize different flux distributions under different conditions. Also this problem is convex (S1 Text section 3.7). If a model contains non-enzymatic reactions (or non-enzymatic processes such as metabolite diffusion out of the cell or dilution in growing cells), each such reaction leads to an extra constraint on the metabolite polytope (S1 Text section 3.8). A known flux in an irreversible diffusion or dilution reaction fixes the concentration of one metabolite. In the presence of irreversible non-enzymatic reactions with mass-action rate laws, the polytope is intersected by a subspace. In both cases, the resulting sub-polytope may be empty, i.e., the given flux distribution will not be realizable.

### Non-stationary states and the importance of boundary metabolites

Flux balance analysis and kinetic models rely on the assumption that certain metabolites are mass-balanced: in FBA, this assumption, together with stationarity, defines the set of steady-state fluxes; in kinetic models, the mass-balanced metabolites are the ones whose dynamics is described by the system equations. ECM, in contrast, assumes fluxes to be given and makes no assumption about mass balances. If the fluxes in our pathway model lead to a mass imbalance in a metabolite, we may still assume that the entire cell is in stationary state, but that mass balances are reached with the help of other pathways that are not part of our model. Alternatively, we may assume that the metabolite is actually not mass-balanced and that we are describing a non-stationary, transient state. In both cases, ECM is fully applicable as long as metabolic fluxes are predefined and loop-less (in order to be realizable by a thermodynamically consistent state [67]).

A key point in ECM is the choice of metabolite levels on the model boundary. If we predefine all these metabolite levels, our pathway will be “isolated” from the rest of the network, and any information about the surrounding network can be safely ignored. In our *E. coli* model, ATP is one such important boundary metabolite: if we allowed for a lower ATP level, ATP could be produced at a lower enzyme cost because of the more favorable driving forces. If we do not fix the ATP concentration, but define an allowed range, ECM would choose the lowest possible ATP level; thus, if the allowed ATP range is too broad, no meaningful predictions can be expected. In a model of ATP-consuming biosynthesis pathways, the situation would be exactly the opposite: here, it is a high ATP level that would lead to higher driving forces and to lower enzyme requirements. Whole-cell models contain both types of pathways—ATP-producing and ATP-consuming ones. In such a model, ECM could predict some meaningful compromise, i.e. an intermediate ATP level that minimizes the enzyme cost of ATP production *plus* the enzyme cost of biosynthesis. Since in our central metabolism model there are only 3 reactions that produce or consume ATP, it is unlikely that so few reactions would be representative of the cost tradeoff between the dozens of enzymes that use ATP in the full metabolic network. Therefore, we chose to fix the ATP level to its measured value (∼3 mM).

Our use of small-scale pathway models, which ignore most of the metabolic network, is therefore justified: as long as we predefine all metabolic fluxes and all metabolite levels at the pathway boundary, pathways can be modeled separately and the models can later be combined without any adjustment. This makes the ECM approach fully modular. All the input parameters (kinetic parameters, fluxes and boundary concentrations) are directly obtained from measurements without any further tuning. By setting all relevant fluxes and boundary metabolite concentrations to their measured values, we isolate our submodel from any effects that the surrounding network might have on predicted enzyme cost. Finally, there may be metabolites that are “free” in a model, but that affect enzymes that are not in the model (e.g. pyruvate, which affects 30 other enzymes). By neglecting these enzymes, we ignore some of the complex compromises between them, and the predicted metabolite concentration may be wrong. In our specific case of central metabolism, whose enzymes comprise a very large fraction of *E. coli*’s proteome, this effect is probably not so severe. In any case, this problem can be easily fixed by imposing constraints or fixed concentrations for central metabolites such as pyruvate (similar to how we deal with ATP and other co-factors).

### Tolerance ranges for nearly optimal solutions

Evolution could tolerate non-optimal enzyme costs; this tolerance depends on population dynamics and can sometimes be quite significant, e.g. in small isolated communities. To compute realistic tolerance ranges for the ECM problem, we start from the optimum (total cost *q*) and choose a tolerable cost *q*^tol^ (e.g., one percent higher than the optimal cost). This defines a tolerable region in $P:P^{\text{tol}}\equiv \left\{x\in P\;|\;q\left(x\right)\leq q^{\text{tol}}\right\}$. A tolerance range for each metabolite is defined by the minimal and maximal values the metabolite can show within $P^{\text{tol}}$. Tolerance ranges for enzyme levels are defined in a similar way. Alternatively, tolerance ranges and nearly optimal solutions can be estimated from the Hessian matrix (see S1 Text section 7.3).

### Sensitivity analysis with respect to kinetic constants

The predicted enzyme and metabolite levels depend on the kinetic model chosen, and in particular on the kinetic constants (*k*_cat_ and *K*_M_ values). Errors or uncertainties in these constants will cause errors or uncertainties in the predicted enzyme profiles. To estimate these uncertainties, we considered a joint distribution of all model parameters, describing both the uncertainties of individual parameters and the correlations between dependent parameters. This probability distribution was directly obtained from parameter balancing (S1 Text section 5.2). We sampled the kinetic parameters from this distribution, sampled metabolic fluxes according to their experimental mean values and standard deviations, and varied the fixed metabolite levels in a ± 5% range around their standard values. Then we applied ECM on each of the sampled parameter sets, and gathered statistics for the optimal enzyme and metabolite levels. Fig 4(a) shows the distributions of the predicted enzyme levels. For narrow parameter distributions, the mean values, variances, and covariances of the predicted enzyme levels can even be computed, approximately, from the ECM solution with standard parameters (see S1 Text section 3.5). Enzyme uncertainties caused by parameter uncertainties should not be confused with the tolerance ranges described before. The tolerance ranges are always associated with sub-optimal solutions, i.e., enzyme profiles with a higher total cost; enzyme variations caused by parameter variation, in contrast, can go both ways and may sometimes decrease the cost.

### Testing the hypothesis of cost-optimal metabolite profiles

Our enzyme level predictions rely on two main assumptions: a mechanistic model that defines a quantitative relation between metabolite levels, enzyme levels, and fluxes, and an optimality assumption stating that metabolite levels are optimized for a minimal total enzyme cost. To test whether such cost optimality holds in reality, we used the same mechanistic model and predicted enzyme levels based on feasible, randomly sampled metabolite profiles. We first sampled metabolite profiles around the ECM optimum by adding normally distributed random numbers (standard deviation 0.05, for metabolite levels on natural log scale); then we sampled metabolite profiles in a much wider range, by sampling convex combinations of extreme points in the metabolite polytope (i.e., points realizing minimal or maximal values of individual metabolite concentrations). As shown in Fig 4(b), the metabolite profiles close to the ECM optimum yield significantly better enzyme level predictions than broadly sampled metabolite profiles. The fact that predictions from the same kinetic model, without the optimality assumption, become much worse provides strong support for cost-optimality as a principle in living cells.

### Workflow for model building and enzyme prediction

**Fig 8:**
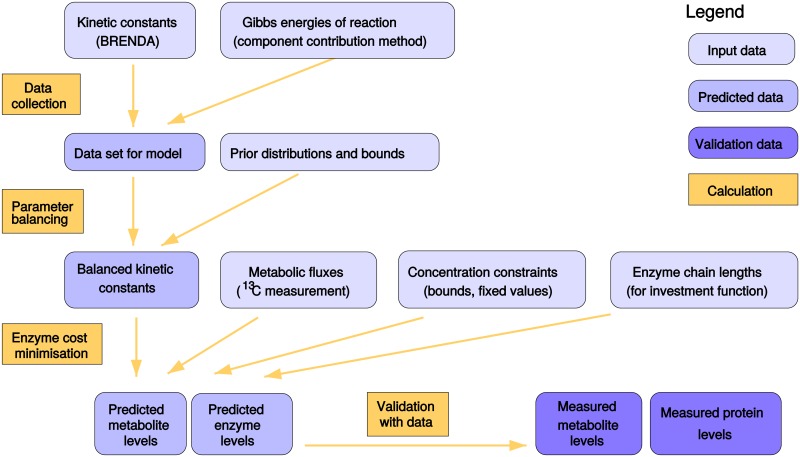
Data integration in the ECM-based modeling workflow. After collecting all available kinetic and thermodynamic data and mapping them onto the network model, we use parameter balancing to obtain a consistent, complete set of kinetic constants. For a fully parameterized kinetic model, the metabolite and enzyme levels must be determined. We compute them by enzyme cost minimization with predefined metabolic fluxes (obtained from experiments or computationally). Finally, the predicted values are validated with measured metabolite and protein concentrations.

To predict enzyme and metabolite levels in metabolic pathways we developed an automated workflow (Fig 8). In a consistent model, all parameters must satisfy Wegscheider conditions for equilibrium constants [68] and Haldane relationships between equilibrium constants and rate constants [69]. The kinetic constants used in rate laws should represent *effective* parameters, which may differ from “ideal” parameters, e.g., by crowding effects. However, since measured parameter values are usually incomplete and inconsistent, parameter balancing [47] is used to translate measured kinetic constants into consistent model parameters. Based on a network and given fluxes, the software extracts relevant data from a database (thermodynamic constants, rate constants, fluxes, and protein sizes; metabolite and protein levels for validation), determines a consistent set of model parameters, builds a kinetic model, and optimizes enzyme and metabolite profiles for the EMC function chosen. To assess the effects of parameter variation, parameter sets can be sampled from the posterior distribution as described above. The workflow has been implemented in MATLAB and uses Systems Biology Markup Language (SBML) for model structures and the SBtab table format for numerical data [70].

### *E. coli* model

The model shown in Fig 3 was built automatically from a list of chemical reactions in *E. coli* central metabolism (for details, see S1 Text section 6). Equilibrium constants were estimated using the component contribution method [41], kinetic constants ($k_{\text{cat}}^{+}$ and *K*_M_ values) were obtained from the BRENDA database (after which each value was curated manually), and a complete, globally consistent parameter set was determined by parameter balancing. During ECM, all metabolite levels were limited to predefined ranges, and the levels of cofactors and some other metabolites were fixed at experimentally known values. To compute tolerances for predicted metabolite and enzyme levels, we defined an acceptable enzyme cost, one percent higher than the minimal value, and determined ranges for metabolite levels that agree with this cost limit. The enzyme cost function accounts for protein composition, giving different costs to different amino acids. However, models with equal cost weights for all proteins, or with size-dependent protein costs yielded similar results (results are provided on the website). Data, model, and MATLAB code for ECM can be obtained from www.metabolic-economics.de/enzyme-cost-minimization/.

## Supporting Information

S1 Text Supplementary material text containing Figure A—Figure F and Table A—Table C. (PDF)
