# Supplementary material for: The Protein Cost of Metabolic Fluxes: Prediction from Enzymatic Rate Laws and Cost Minimization
Source: PLoS Comput Biol. 2016 Nov 3;12(11):e1005167. doi: 10.1371/journal.pcbi.1005167 (PMC5094713; doi:10.1371/journal.pcbi.1005167)
Supplement: S1 Text — (PDF) [file pcbi.1005167.s001.pdf]

The protein cost of metabolic fluxes: prediction from enzymatic rate laws and cost minimization

Supplementary information

Elad Noor[1], Avi Flamholz[2], Arren Bar-Even[3], Dan Davidi[4], Ron Milo[4], Wolfram Liebermeister[5]

[1]Institute of Molecular Systems Biology, Eidgenössische Technische Hochschule Zürich, Switzerland, [2]Department of Molecular and Cellular Biology, University of California, Berkely, California, United States of America, [3]Max Planck Institute for Molecular Plant Physiology, Golm, Germany, [4]Department of Plant Sciences, The Weizmann Institute of Science, Rehovot, Israel, [5]Institute of Biochemistry, Charité – Universitätsmedizin Berlin, Germany

1 Kinetic rate laws

1.1 Rate laws for general enzymatic reactions

Reversible rate laws for reactions with multiple substrates (concentrations s_i) and products (concentrations p_j) have the form

$$v \;=\; E\,\frac{k_{\rm cat}^{+}\prod_i (\frac{s_i}{K_i})^{m_i^{\rm S}} - k_{\rm cat}^{-}\prod_i (\frac{p_i}{K_i})^{m_i^{\rm P}}}{D(s_1,s_2,..,p_1,p_2,..)}. \tag{S1.1}$$

By default, we assume that an enzyme molecule contains a single catalytic site. If an enzyme is a protein complex with $N_{\rm sub}$ subunits and $N_{\rm cat}$ catalytic sites, we can use effective values ${k_{\rm cat}^{+}}' = \frac{N_{\rm cat}}{N_{\rm sub}}\,k_{\rm cat}^{+}$ referring to single enzyme subunits, whose concentrations E are recorded in proteomics data. The molecularities $m_{li}^{\rm S}$ or $m_{li}^{\rm P}$ describe in what numbers reactants participate in the enzyme mechanism. Molecularities can differ from the (nominal) stoichiometric coefficients by a reaction-specific scaling factor γ because the stoichiometric coefficients in the sum formula may be arbitrarily scaled. For example, in a reaction 2 A + 4 B → 2 C (stoichiometric coefficients -2, -4, 2) with th rate law $k_{\rm cat}^{+}\,[A]\,[B]^2 - k_{\rm cat}^{-}\,[C]$ (with molecularities 1, 2, 1), this factor would be $\gamma = 1/2$. If we assume, as it is usually done, that the exponents in the rate law represent stoichiometric coefficients, the factor γ_l will appear like an effective Hill coefficient. In turn, a reactant with stoichiometric coefficient n_{il} and an effective Hill coefficient $\gamma = 2$ will have a molecularity of $m_{li}^{\rm S} = 2|n_{il}|$. For reasons of thermodynamic consistency (existence of a consistent equilibrium state), all substrates and products in a reaction must show the same γ factors [1].

The signs and magnitudes of metabolic fluxes depend on thermodynamic driving forces (see Figure A). We define the thermodynamic driving force as the negative reaction Gibbs energy $-\Delta_{\rm r}G'$, measured in units of RT. The symbol G' denotes transformed Gibbs free energies, suitable variables for systems at given or buffered pH value. To obtain the correct relationship between fluxes and driving forces, the driving forces Θ_l must be defined based on molecularities, not on stoichiometric coefficients[1]. According to thermodynamics, all reaction rates must vanish in chemical equilibrium; to ensure this in kinetic models, equilibrium constants and rate constants must satisfy

[1]If stoichiometric coefficients and molecularities differ, the Hill-like coefficient γ_l must appear in the definition of driving forces $\Theta_l = -\frac{1}{RT}\sum_i \gamma_l\, n_{il}\, G'_l = -\gamma_l\,\Delta_{\rm r}G'_l$. The difference along a reaction is not defined based on nominal stoichiometric coefficients, but on actual molecularities.

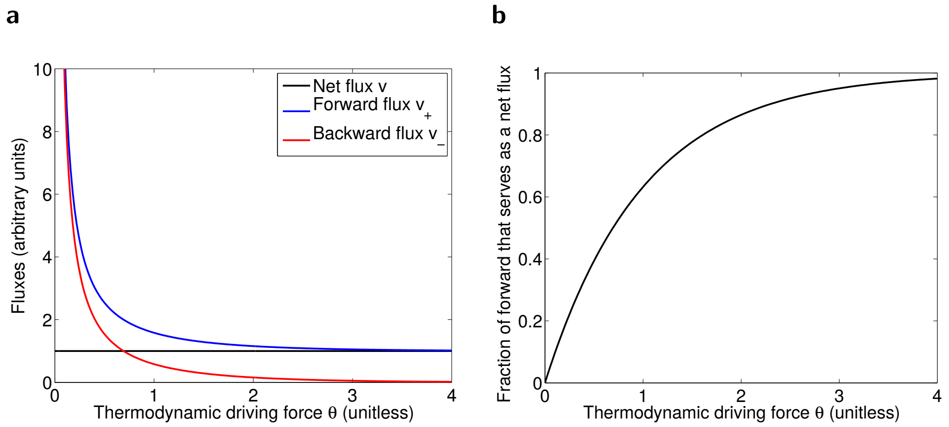

Figure A: **Enzyme efficiency depends on thermodynamics.** The thermodynamic driving force $\Theta = -\Delta G'/RT$ in a reaction determines the ratio between forward and backward fluxes: $v^{+}/v^{-} = \mathrm{e}^{\Theta}$. If the net flux $v = 1$ is kept fixed, forward and backward fluxes strongly increase as Θ approaches 0 (chemical equilibrium). (a) Forward (blue) and backward (red) flux as functions of the thermodynamic force. In each point, their difference yields the predefined net flux $v = 1$. (b) Only a fraction of the forward flux v^{+} acts as a net flux, while the rest is canceled by the backward flux. This fraction varies between 0 (no thermodynamic force, chemical equilibrium) and 1 (high thermodynamic force, strongly driven reaction).

the Haldane relationship [2]

$$K_{\mathrm{eq}} = \frac{\prod_i (s_i^{\mathrm{eq}})^{m_i^{\mathrm{S}}}}{\prod_i (p_i^{\mathrm{eq}})^{m_i^{\mathrm{P}}}} = \frac{k_{\mathrm{cat}}^{+} \prod_i (K_i)^{m_i^{\mathrm{P}}}}{k_{\mathrm{cat}}^{-} \prod_i (K_i)^{m_i^{\mathrm{S}}}}, \tag{S1.2}$$

where s_i and p_j denote substrate and product levels, respectively. Moreover, the equilibrium constants follow from Gibbs energies of formation as $K_{\mathrm{eq}} = \mathrm{e}^{-\Delta_{\mathrm{r}} G^{\circ\prime}/RT}$. This implies Wegscheider conditions [3]: the vector of equilibrium constants satisfies $\ln K_{\mathrm{eq}} = \mathbf{N}_{\mathrm{tot}}{}^{\top} \boldsymbol{\mu}^{\circ\prime}$, with the stoichiometric matrix $\mathbf{N}_{\mathrm{tot}}$ for all metabolites and the vector $\boldsymbol{\mu}^{\circ\prime}$ of transformed Gibbs free energies of formation. Accordingly, the equilibrium constants must satisfy a Wegscheider condition $\ln K_{\mathrm{eq}} \cdot \mathbf{k} = 0$ for any thermodynamic cycle $\mathbf{k}$, i.e., any nullspace vector of $\mathbf{N}_{\mathrm{tot}}{}^{\top}$.

The formula for the denominator D in Eq. (S1.1) depends on the enzyme mechanism assumed. A general, biochemically plausible choice is polynomials of the form

$$D(\mathbf{c}) = 1 + \sum_k M_{lk} \prod_i c_i^{m_{lik}} \tag{S1.3}$$

with positive coefficients M_{lk} and exponents m_{lik}. Each sum term (index k) represents a binding state of the enzyme. The exponents m_{lik} encode the numbers of bound reactant molecules and the prefactors encode the binding energies. The sum term 1 represents the unbound enzyme. The highest-order substrate term reads $\prod_i (s_i/K_i)^{m_i^{\mathrm{S}}}$ and the highest-order product term reads $\prod_i (p_i/K_i)^{m_i^{\mathrm{P}}}$. The denominator may also contain additive or multiplicative terms for allosteric activation and inhibition. While the exponents m_{lik} are usually positive integers, allosteric regulation can imply denominator terms K_{S}/s. A special case of Eq. (S1.3) are rate laws for polymerization reactions [4], which can also be used as simplified rate laws for biomass-producing reactions. In this case, it will be the "template" molecules rather than the enzyme that is scored by a cost. By focusing on simple enzyme mechanisms with few binding states, we obtain general rate laws that are valid for all reaction stoichiometries. Their denominators have simple structures (containing only few sum terms and a few Michaelis-Menten constants as parameters) [1]. Since these rate laws containing fewer denominator terms than more complex rate laws, the rates become higher and enzyme demand and costs tend to be underestimated. The

reversibility-based EMC2 functions are based on rate laws with the denominators

$$\begin{aligned} D^{\mathrm{S}} &= \prod_i (s_i/K_i)^{m_i^{\mathrm{S}}} \\ D^{\mathrm{SP}} &= \prod_i (s_i/K_i)^{m_i^{\mathrm{S}}} + \prod_j (p_j/K_j)^{m_i^{\mathrm{P}}}. \end{aligned} \tag{S1.4}$$

The mathematical products are called mass-action terms. In the first formula, we assume that substrate levels are high and product levels are low; and in the second one, that both substrate and product levels are high. The saturation-based EMC3 functions are based on rate laws with the denominators

$$\begin{aligned} D^{1\mathrm{S}} &= 1 + \prod_i (s_i/K_i)^{m_i^{\mathrm{S}}} \\ D^{1\mathrm{SP}} &= 1 + \prod_i (s_i/K_i)^{m_i^{\mathrm{S}}} + \prod_j (p_j/K_j)^{m_i^{\mathrm{P}}}. \end{aligned} \tag{S1.5}$$

These denominators contain only the term 1 and the substrate and product mass-action terms. To justify these rate laws, we assume a strongly cooperative binding between substrates and between products and consider an enzyme mechanism with only three states: enzyme bound with all substrates, enzyme bound with all products, and unbound enzyme. The first formula assumes low product concentrations, and The second formula describes the direct-binding modular (DM) rate law [1]. The direct-binding modular rate law generalizes the reversible MM kinetics. Furthermore, we consider the common modular rate (CM) law [5, 1], a generalized form of reversible MM kinetics with the denominator

$$D^{\mathrm{CM}} = \prod_i (1 + s_i/K_i)^{m_i^{\mathrm{S}}} + \prod_j (1 + p_j/K_j)^{m_i^{\mathrm{P}}} - 1. \tag{S1.6}$$

In the enzyme mechanism, substrate molecules bind independently, product molecules bind independently, and substrate and product binding exclude each other. Multiplying out the denominator (S1.6), we obtain many more terms than in the direct-binding modular rate law. Realistic rate laws will contain more denominator terms than the DM rate law, but possibly fewer than the CM rate law. To interpolate between the two extremes, we may take their arithmic or geometric mean

$$D^{\mathrm{geom}} = \sqrt{D^{\mathrm{DM}}\, D^{\mathrm{CM}}}, \qquad D^{\mathrm{arith}} = \frac{1}{2}\, D^{\mathrm{DM}} + \frac{1}{2}\, D^{\mathrm{CM}}. \tag{S1.7}$$

If the denominator values D^{DM} and D^{CM} are not too different, the two mean values will be similar[2]. In the second formula (arithmetic mean), the mass-action terms appear as in DM and CM rate laws, and all other terms from the CM law appear with prefactors of $\frac{1}{2}$. If we define rate laws by taking a geometric (or arithmetic) mean of rate laws denominators, the corresponding enzyme costs will be given by geometric (or harmonic) mean values of enzyme costs. If an enzyme is allosterically regulated, this can be described by additive or multiplicative regulation terms in the rate law denominator [1]. Additive terms arise from competitive regulation. Multiplicative terms (for non-competitive regulation) can be split from the denominator and become prefactors of the rate law. Typical choices are $\frac{x}{x+k_X^{\mathrm{A}}}$ for non-competitive activation and $\frac{k_X^{\mathrm{I}}}{x+k_X^{\mathrm{I}}}$ for non-competitive inhibition, with rate constants k^{A} and k^{I} and a regulator concentration x [1]. Thus, in the factorized EMC formulae, allosteric effects can either be listed by a separate efficiency factor or be included in the saturation factor. For instance, the saturation factor for Michaelis-Menten kinetics with non-competitive inhibition can be split into

$$\eta^{\mathrm{sat}} = \frac{s/K_{\mathrm{S}}}{(1 + \frac{x}{K_{\mathrm{I}}})(1 + \frac{s}{K_{\mathrm{S}}} + \frac{p}{K_{\mathrm{P}}})} = \frac{1}{1 + \frac{s}{K_{\mathrm{S}}} + \frac{p}{K_{\mathrm{P}}}} \frac{1}{(1 + x/K_{\mathrm{I}})} = \eta^{\mathrm{sat}*}\, \eta^{\mathrm{reg}}. \tag{S1.8}$$

[2] If $a \approx b$, we can approximate $\sqrt{a\, b} = \sqrt{a\,(a + b - a)} = a\sqrt{1 + \frac{b-a}{a}} \approx a(1 + \frac{1}{2}\frac{b-a}{a}) = \frac{1}{2}[a + b]$.

1.2 How the efficiency factors are derived

The general formula S1.1 covers a wide range of possible rate laws. To demonstrate how it can be factorized into the capacity and efficiency factors, we consider a bimolecular reaction $A + B \rightleftharpoons P + Q$ and an enzyme with a common modular (CM) rate law (Equation S1.6), i.e.

$$\begin{aligned} v &= E \frac{k_{\rm cat}^{+} \frac{[A][B]}{K_A\, K_B} - k_{\rm cat}^{-} \frac{[P][Q]}{K_P\, K_Q}}{(1+\frac{[A]}{K_A})(1+\frac{[B]}{K_B}) + (1+\frac{[P]}{K_P}((1+\frac{[Q]}{K_Q}) - 1} \\ &= E\, k_{\rm cat}^{+} \frac{\frac{[A][B]}{K_A\, K_B} - \frac{k_{\rm cat}^{-}}{k_{\rm cat}^{+}} \frac{[P][Q]}{K_P\, K_Q}}{1 + \frac{[A]}{K_A} + \frac{[B]}{K_B} + \frac{[A][B]}{K_{AB}} + \frac{[P]}{K_P} + \frac{[Q]}{K_Q} + \frac{[P][Q]}{K_{PQ}}} \\ &= E\, k_{\rm cat}^{+} \underbrace{\left(1 - {\rm e}^{-\Theta}\right)}_{\eta^{\rm enr}} \underbrace{\frac{\frac{[A][B]}{K_A\, K_B}}{1 + \frac{[A]}{K_A} + \frac{[B]}{K_B} + \frac{[A][B]}{K_{AB}} + \frac{[P]}{K_P} + \frac{[Q]}{K_Q} + \frac{[P][Q]}{K_{PQ}}}}_{\eta^{\rm sat}} \end{aligned} \tag{S1.9}$$

where for the last step, we used the Haldane relationship $K_{\rm eq} = \frac{k_{\rm cat}^{+}}{k_{\rm cat}^{-}} \frac{K_P\, K_Q}{K_A\, K_B}$ and the identity ${\rm e}^{-\Theta} = \frac{[P][Q]}{[A][B]}/K_{\rm eq}$. This Haldane relationship and the connection between the thermodynamic driving force and the ratio between the numerator terms hold in general:

$$\frac{k_{\rm cat}^{+} \prod_i (\frac{s_i}{K_i})^{m_i^{\rm S}}}{k_{\rm cat}^{-} \prod_i (\frac{p_i}{K_i})^{m_i^{\rm P}}} = \frac{\prod_i s_i^{m_i^{\rm S}}}{\prod_i p_i^{m_i^{\rm P}}} \cdot \frac{k_{\rm cat}^{+} \prod_i K_i^{m_i^{\rm P}}}{k_{\rm cat}^{-} \prod_i K_i^{m_i^{\rm S}}} = \frac{\prod_i s_i^{m_i^{\rm S}}}{\prod_i p_i^{m_i^{\rm P}}} / K_{\rm eq} = {\rm e}^{-\Theta}. \tag{S1.10}$$

Thus we can obtain the general factorized rate law:

$$v = E \frac{k_{\rm cat}^{+} \prod_i (\frac{s_i}{K_i})^{m_i^{\rm S}} - k_{\rm cat}^{-} \prod_i (\frac{p_i}{K_i})^{m_i^{\rm P}}}{D(s_1, s_2, .., p_1, p_2, ..)} = E\, k_{\rm cat}^{+} \underbrace{\left(1 - {\rm e}^{-\Theta}\right)}_{\eta^{\rm enr}} \underbrace{\frac{\prod_i (s_i/K_i)^{m_i^{\rm S}}}{D(s_1, s_2, .., p_1, p_2, ..)}}_{\eta^{\rm sat} \cdot \eta^{\rm reg}}. \tag{S1.11}$$

2 Enzyme cost functions

To quantify enzyme cost, we assume it is proportional to the concentration of that enzyme. Potentially, each enzyme level can be weighted by different enzyme-specific costs. Are such cost weights biologically justified? We now discuss the relevance of these costs and show how the linearity assumption, combined with separable rate laws, yields simple factorized enzyme cost functions.

2.1 What factors determine the cost per enzyme molecule?

In ECM, we assume that cells realize their metabolic fluxes at a minimal enzyme cost and that this cost is a direct function of the enzyme levels. We further assume that the cost function is linear, i.e. $h(E_1, E_2, ..) = \sum_l h_{E_l} E_l$. The values of the enzyme-specific costs h_{E_l} depend on the biological context. For instance, cast can be defined by a growth deficit caused by enzyme over-expression. In microbes, such cost values can be measured using standard lab techniques for measuring growth rate. A disadvantage of this approach is the difficulty to disentangle the cost of the specific over-expressed enzyme from other effects that the enzyme could have on the metabolic network at large, most importantly the potential benefit of increasing the flux in the reaction it catalyzes. Theoretical approaches, on the other hand, can be used to isolate enzyme cost from global effects, but may not capture the many possible ways in which growth deficits are caused in reality. Aside from the resources required for production and maintenance, enzymes need to compete with other proteins and macromolecules for the limited space in the

cytoplasm or on membranes [6, 7]. The restriction can be related to the volume of the protein, the occupied membrane surface area, and the effect it has on the osmotic pressure (which depends on electro-static interactions with the surrounding water). Therefore, protein cost is a complex function of the copy-number of the enzyme, its physico-chemical parameters (such as molecular weight, 3D structure, hydrophobicity, charge, etc.), and its production or degradation rate. Furthermore, enzymes can have adverse side effects, e.g., by promiscuous activity [8], which are virtually impossible to predict without extensive knowledge about an organism's full metabolic network and physiology. Since many of these features are unknown for most enzymes, and some of these effects require elaborate 3D models, which are beyond the scope of this work, we try to define a cost function that is simple to calculate, but captures many of these biological aspects. To determine relative cost weights, we may simply assume that enzyme cost is proportional to enzyme mass. Since we only use total cost as an optimization goal, the problem is scale-free and therefore the relative cost weights are enough for ECM. We obtain a linear cost function with specific costs $h_{E_l} \sim L_l$, where L_l is a measure of protein size (e.g., length in amino acid units or mass in Daltons). This may be relevant, in particular, for protein complexes or for lumped reactions representing entire pathways. This formula for protein cost weights can be extended by other factors:

- **Degradation rate and protein lifetime** To account for differences in protein degradation, we can assume that enzyme cost is proportional to the enzyme production rate (in units of amino acids or Daltons per second). We define the lifetime of the enzyme as $\tau_l = (\kappa_l + \lambda)^{-1}$ (where λ is the growth rate κ_l is the degradation rate[3]), and therefore the cost would be $h_{E_l} \sim L_l/\tau_l$. When the cell growth rate is much faster than the degradation rate, $\kappa_l \ll \lambda$, all enzymes have approximately the same lifetime and therefore the effect of protein degradation would be negligible.

- **Individual amino acid costs** Enzymes show different amino acid compositions, and different amino acids require different amounts of energy for their production. If enzyme cost is mainly due to investments in amino acid production, we can quantify the energetic and material costs of individual amino acids [9] and account for them in our choice of enzyme cost weights. We did this in our calculations, but other cost functions, in which amino acid composition is neglected, lead to similar predictions of enzyme levels.

- **Enzyme complexes with multiple subunits and catalytic sites** An enzyme may consist of several protein subunits and may contain several catalytic sites. Therefore, we adopt the convention that $k_{\rm cat}$ values refer to catalytic sites, while protein levels refer to protein subunits. The number $N_{\rm sub}$ of complex subunits and the number $N_{\rm cat}$ of catalytic sites per complex must appear in the formulae for reaction rates, enzyme demand, and enzyme cost: we replace in all these formulae the $k^+_{\rm cat}$ value (referring to a single catalytic site) by an effective value ${k^+_{\rm cat}}' = \frac{N_{\rm cat}}{N_{\rm sub}} k^+_{\rm cat}$ (referring to one subunit).

- **Covalent modification** Enzyme activity can be changed by phosphorylation or other posttranslational modifications. So far, we assumed that enzymes exist in one form and that the enzyme level E_l represents their concentration. For modifiable enzymes, our variable E_l describes the concentration of enzyme molecules *in the right modification state*, which is only a fraction $\rho_l < 1$ of the total concentration. Since, the total enzyme concentration is $1/\rho_l$ times as large as the concentration E_l appearing in the rate law, the enzyme cost weight h_{E_l} must be increased by this factor $1/\rho_l$.

- **Constrained enzyme levels** The enzyme amounts in cells are restricted by physical constraints (e.g. space restrictions on mitochondrial membranes, which limit the number of respiration complexes). In ECM, this could be described by imposing upper limits on sums of enzyme levels in the cell, in cell compartments, or within membranes. As a heuristics, such constraints can also be replaced by cost terms that penalize high levels of these enzymes[4].

[3]Protein lifetimes may vary systematically between types of reactions catalyzed. Enzymes catalyzing oxidation reactions are likely to accumulate damages faster and can be expected to have shorter lifetimes.

[4]To justify such cost terms mathematically, one could first consider a model with constraints on some enzyme fractions. These

Function	Denominator	Rate law $r(\mathbf{c})$
EMC0	1	const.
EMC1	1	$k_{\rm cat}^{+}$
EMC2s	$D^{\rm S} = S$	$k_{\rm cat}^{+} \eta^{\rm rev}$
EMC2sp	$D^{\rm SP} = S + P$	$k_{\rm cat}^{+} \frac{\eta^{\rm rev}}{1+K_{\rm eq}\,{\rm e}^{-\Theta}}$
EMC3s	$D^{\rm 1S} = 1 + S$	$k_{\rm cat}^{+} \frac{\eta^{\rm rev}\,S}{1+S}$
EMC3sp	$D^{\rm 1SP} = 1 + S + P$	$k_{\rm cat}^{+} \frac{S\,\eta^{\rm rev}}{1+S+P}$
EMC4cm	$D^{\rm CM} = S^{\rm CM} + P^{\rm CM} - 1$	$k_{\rm cat}^{+} \frac{S\,\eta^{\rm rev}}{S^{+}+P^{+}-1}$
EMC4geom	$D^{\rm geom}$	$k_{\rm cat}^{+} \frac{S\,\eta^{\rm rev}}{\sqrt{D^{\rm CM}\,D^{\rm 1SP}}}$
EMC4arith	$D^{\rm arith}$	$k_{\rm cat}^{+} \frac{S\,\eta^{\rm rev}}{\frac{1}{2}(D^{\rm CM}+D^{\rm 1SP})}$
EMC4	$D(\mathbf{c})$	$k_{\rm cat}^{+} \frac{S\,\eta^{\rm rev}}{D(\mathbf{c})}$

Quantity	Formula
Reaction rate	$v = E\,r$
Enzyme demand	$E = v/r$
Enzyme cost	$q = h\,E = \frac{h\,v}{r}$
Flux-specific cost	$a_{\rm v} = \frac{q}{v} = \frac{h}{r}$

Table A: Rate laws and enzyme-based metabolic cost (EMC) functions. First table: EMC functions derived from simplified rate laws. Abbreviations: Forward catalytic constant $k_{\rm cat}^{+}$. Energy efficiency $\eta^{\rm rev} = 1 - {\rm e}^{-\Theta(\mathbf{c})}$. Mass-action denominator terms $S = \prod_i (s_i/K_i)^{m_i^{\rm S}}$, $P = \prod_j (p_j/K_j)^{m_j^{\rm P}}$; convenience denominator terms $S^{\rm CM} = \prod_i (1 + s_i/K_i)^{m_i^{\rm S}}$; $P^{\rm CM} = \prod_j (1 + p_j/K_j)^{m_j^{\rm P}}$. The denominator $D(\mathbf{c})$ in the EMC4 function is a polynomial with non-negative coefficients as in Eq. (S1.3); it can also contain terms describing allosteric regulation. The molecularities $m^{\rm s}$ and $m^{\rm p}$ represent stoichiometric coefficients, but they can contain reaction-specific Hill coefficients as prefactors. The second table lists some quantities derived from the rate laws.

- **Lumped reactions** In a model, series of reactions can be represented by lumped reactions. Effective parameters (h and $k_{\rm cat}^{+}$ values) for lumped reactions can be obtained as described in 7.2.
- **Absolute scaling** Beyond ECM, some applications require an absolute scaling of the cost function, i.e. the cost must be in units that are comparable to other factors that affect fitness, such as the biomass flux or the growth rate. This absolute scaling can be determined based on experimental data, for instance, by matching measured growth deficits for GFP [10]. Alternatively, we can convert the other fitness terms to units of enzyme mass, e.g. by quantifying how the biomass flux generates the amino acids that are eventually used to synthesize the enzymes.
- **Convex non-linear cost functions** Finally, if a nonlinear cost function $h(\boldsymbol{E})$ is used, the total cost of a pathway is not simply a sum over the reactions' enzyme costs. Instead, a high cost in one enzyme could increase the cost pressure on other enzymes. Nevertheless, if the cost functions is convex, the total cost remains a convex function on the metabolite polytope, so numerical optimization stays feasible.

2.2 Enzyme cost as a function of metabolite levels

The enzyme cost of a given metabolic flux profile can be cast as a function $q(\ln \mathbf{c})$ on the metabolite polytope. To obtain simple cost functions, we consider the factorized enzyme cost Eq. (6) and approximate some of the terms by constant numbers. Constant values of 1 arise from limiting cases: an infinite driving force leads to an energy factor of 1, and if enzymes are fully substrate-saturated and product concentrations are small ($a \gg k_a^{\rm M}$, $b \ll K_{{\rm M}b}$), the saturation factor can be set to 1. To approximate the true cost function, we can start from the most simple estimate (EMC1) and subsequently reintroduce the different efficiency factors. The enzyme cost functions can be grouped, according to the data required, into five levels (see Tables 1 and A):

- **EMC0 ("sum of fluxes")** If no enzyme parameters are known at all, we can assume the same flux-specific cost $a_{\rm v}$ for all enzymes. Enzyme levels and enzyme costs are proportional to fluxes across the network:

constraints could be treated by Lagrange multipliers, which lead to effective cost terms in the objective function. In these terms, the Lagrange multipliers appear as if they were enzyme cost weights. Replacing these Lagrange multipliers by constant numbers, we obtain effective linear cost terms which we can add to our cost function.

$E_l \sim q_l \sim v_l$, and enzyme cost is proportional to the sum of fluxes (where fluxes are positive due to our convention about reaction orientations).

- **EMC1 ("capacity-based")** In the capacity-based (EMC1) functions, enzymes have individual specific flux costs $a_{v_l} = h_{E_l}/k^{+}_{\mathrm{cat},l}$ (based on known k^{+}_{cat} and h values) and are independent of metabolite levels. This is equivalent to replacing reaction rates v by $v_{\max}$ values, or dropping the efficiency factors in Eq. (6). Alternatively, we can set each factor to a constant, enzyme-specific value.

- **EMC2 ("reversibility-based")** The reversibility-based (EMC2) functions capture the fact that cost increases close to equilibrium. They depend on metabolite levels, but only via the driving forces only, and equilibrium constants need to be known for the calculation. In the EMC2s function, we assume that enzymes are strongly substrate-saturated while product saturation is negligible: the denominator D^{S} (see Eq. (S1.4)) cancels the numerator term, resulting in a constant saturation factor $\eta^{\mathrm{sat}} = 1$ (i.e., full substrate saturation). The EMC2sp function, another reversibility-based function, describes enzymes with strong substrate and product saturation (denominator D^{SP}). Here the saturation factor

$$\eta^{\mathrm{sat}} = \frac{\prod_i s_i/K_i}{\prod_i s_i/K_i + \prod_j p_j/K_j} = \frac{1}{1 + \frac{\prod_j p_j/K_j}{\prod_i s_i/K_i}} = \frac{1}{1 + K_{\mathrm{eq}}\,\mathrm{e}^{-\Theta}} \tag{S1.12}$$

  is not a constant, but it depends on the driving force. Since the rate can be computed from driving forces alone, the EMC2sp function is classified as "reversibility-based".

- **EMC3 ("saturation-based")** The saturation-based (EMC3) functions represent rate laws with the denominators $D^{(1S)}$ and $D^{(1SP)}$. These are rate laws that do not depend on metabolite levels, but on their mathematical products, the mass-action terms. To compute them, the K_{M} values (more precisely, K_{M} values multiplied over all substrates or products) must be known. The EMC3sp function follows from the direct-binding rate law or, for unimolecular reactions, from reversible Michaelis-Mention kinetics. The EMC3s function has a similar form, but contains no product term. It describes enzymes with incomplete substrate saturation, but far from equilibrium ($\Theta \rightarrow \infty$), so the product term can be neglected. The energy factor η^{rev} can be set to 1, but the factor

$$\eta^{\mathrm{sat}} = \frac{\prod_i (s_i/K_i)^{m_i^{\mathrm{S}}}}{\underbrace{1 + \prod_i (s_i/K_i)^{m_i^{\mathrm{S}}}}_{D^{1\mathrm{S}}}} \tag{S1.13}$$

  remains an explicit term in the rate law.

- **EMC4 ("Kinetics-based")** The kinetics-based cost functions (EMC4) capture all thermodynamically feasible rate laws, including rate laws with allosteric activation or inhibition terms. Their denominators have the form Eq. (S1.3) and contain the terms from the $D^{1\mathrm{SP}}$ denominator, plus others. Examples are rate laws with D^{CM}, D^{geom}, and D^{arith} denominators.

Here are some additional remarks.

- To obtain simplified EMC functions, we can apply the following simplifications: (i) neglect individual enzyme cost weights h; (ii) neglect individual catalytic constants k^{+}_{cat}; (ii) set η^{rev} to a constant value; (iii) set η^{sat} to a constant value. (iv) If η^{sat} is not set constant, (iv.a) use/do not use the term 1 in denominator; (iv.b) use/do not use highest-order product term in denominator; (iv.c) with more reactants: use/do not use additional terms; (v) if the enzyme is allosterically regulated: possibly, set regulation term constant. These simplifications can be freely combined. The classification to EMC0, EMC1, EMC2, EMC3, or EMC4 would then be done according to the resulting formula.

- Different EMC functions require different types of input data: $k_{\rm cat}^{+}$ values for EMC1; additionally equilibrium constants (or standard reaction Gibbs energies) for EMC2; additionally, $K_{\rm M}$ values for EMC3; and possibly, more parameters for EMC4.
- If efficiency factors are set to 1 (and not to smaller constant values), each EMC function is a lower estimate of the following (less simplified) ones. This includes EMC0 function if we use the *largest* $a_{v_l}^{\rm cat}$ value from the other functions as a prefactor in the EMC0 function.
- If a rate law contains Hill coefficients, they can be treated as part of the molecularities. The reactants of a reaction must have the same Hill coefficient.
- Given two possible rate laws for the same reaction, we may define new rate laws by taking their geometric or harmonic mean (this is, for instance, how the rate law denominators $D^{(\rm geom)}$ or $D^{\rm arith}$ were defined). In this case, the enzyme demands and costs (for the new rate law) are given by the geometric or arithmetic means from the original rate laws. In particular, the enzyme cost functions related to the denominators $D^{\rm geom}$ or $D^{\rm arith}$ (called EMC4geom and EMC4arith) represent the geometric (or arithmetic) mean of the original EMC3sp and EMC4cm functions.

2.3 Flux-specific enzyme cost and pathway-specific activity

The flux-specific cost a_{v_l} of an enzyme, in the context of a certain metabolic state, is defined as the enzyme cost per unit flux. At given metabolite levels, and assuming a linear cost function, the flux-specific cost is a constant. At constant metabolite levels, a doubling of the flux will require a doubling of the enzyme level, and thus a doubling of the enzyme cost. The ratio of cost and flux remains constant and is given by Eq. (6). A flux-specific cost can also be defined for pathways or any sets of reactions. Since different reactions may carry different fluxes a pathway (due to non-stationarity, side branches, or splitting of molecules as between upper and lower glycolysis), we choose one flux or production rate as the representative *pathway flux* $v_{\rm pw}$ and define the the *pathway specific cost* by $a_{\rm v}^{\rm pw} = \frac{q}{v_{\rm pw}}$, i.e., the pathway enzyme cost divided by the pathway flux. In practice, the pathway flux should represent a flux that matters for the cell's benefit (e.g., ATP production in a glycolysis model). To compare different pathway models at identical benefits, we could scale their fluxes to the same benefit value. Given fixed metablite levels at the pathway boundaries[5] the flux-specific cost of a pathway will be constant. If all reactions in a pathway carry identical fluxes, it is given by the sum of the reactions' flux-specific costs. Otherwise, the pathway specific cost will be a weighted sum $\sum_l a'_{v_l}\, v'_l$ of the reaction flux-specific costs, with unitless relative fluxes $v'_l = \frac{v_l}{v_{\mathcal{L}}}$ as weights (in a simple linear chain, $v'_l = 1$).

An enzyme's specific activity is given by the catalyzed flux divided by the enzyme mass (in μmol/min/mg enzyme). Specific activities can also be defined for entire pathways [11]. If we treat enzyme mass (in grams/cell volume) as the cost function $h(\boldsymbol{E})$, the resulting flux-specific cost $a_{\rm v}$ (enzyme cost per flux) is exactly the inverse of the specific activity (flux per enzyme mass)[6] . This holds both for single reactions and entire pathways. With enzyme mass used as a cost function, a pathway's specific cost $a_{\rm pw} = q/v_{\rm pw}$ yields the amount of enzyme (in grams/cell volume) divided by the pathway flux (in mM/s) or, in other words, the amount of enzyme (in grams) divided by the pathway flux (in mol/s). Accordingly, the pathway specific activity (in (mol/s)/grams enzyme) is given by

$$A_{\rm pw} = \frac{v_{\rm pw}}{q} = a_{\rm v}^{-1}. \qquad \text{(S1.14)}$$

[5] In a kinetic model with constant external metabolite levels, an overall scaling of enzyme levels will lead to a proportional scaling of fluxes. With a linear enzyme cost function $h(E_1, E_2, ..)$, this scaling will leave all flux-specific costs unchanged. In reality, a change in enzyme levels is likely to affect metabolite levels outside the pathway, so the theoretical result does not exactly apply.

[6] In this case, an enzyme's cost weight h_{E_l} will be given by the enzyme's total mass (in grams/cell volume), divided by the concentration (number of enzyme molecules divided by Avogadro constant and cell volume), so h_{E_l} is just the enzyme's molecular mass in Daltons.

To express this in units of μmol/min/mg enzyme, we multiply by 60000. Since the pathway specific cost is a weighted sum of enzyme specific costs

$$a_{\rm pw} = \sum_l v'_l \, a_{v_l}, \quad \text{(S1.15)}$$

the pathway specific activity $A_{\rm pw}$ (referring to the pathway flux $v_{\rm pw}$) is the weighted harmonic sum of the enzyme specific activities A_l

$$A_{\rm pw} = \left[\sum_l v'_l \, A_l^{-1}\right]^{-1} \quad \text{(S1.16)}$$

where the $v'_l = v_l/v_{\rm pw}$ are scaled (unit-less) fluxes. This formula agrees with the formula given in [11] and allows for non-uniform fluxes along the pathway.

3 Enzyme cost minimization

3.1 Parameterizing the metabolic states of a kinetic model

The standard practice in kinetic modeling is to set up an ODE system where enzyme levels $\boldsymbol{E}$ are given (typically, due to separation of time scales they are assumed to be fixed) and metabolite levels $\mathbf{c}(t)$ are the free variables which evolve over time. The kinetic model describes the relationship between enzymes and metabolites (via kinetic rate laws), which in turn affect the metabolites.

$$\begin{aligned} \dot{\mathbf{c}} &= \mathbf{N}\mathbf{v} \\ \mathbf{v} &= \mathbf{v}(\boldsymbol{E}, \mathbf{c}). \end{aligned} \quad \text{(S1.17)}$$

In order to find a steady state, the ODE is integrated over time until $||\dot{\mathbf{c}}||$ is small enough to be labeled as stationary. Then we can say the system is in steady state and determine the flux and metabolic state $(\mathbf{c}(\infty), \mathbf{v}(\infty))$. In many cases, the steady state will depend on the choice of initial conditions $\mathbf{c}(0)$. We thus define the set of all steady states as $\mathcal{S} = \{(\mathbf{c}(\infty), \mathbf{v}(\infty), \boldsymbol{E})\}_{\boldsymbol{E},\mathbf{c}(0)}$. Using this representation, determining $\mathcal{S}$ requires an exhaustive scan of all parameters $\boldsymbol{E}, \mathbf{c}(0)$, which can be time-consuming, and virtually impossible for large kinetic networks. Here, we suggest an alternative representation of steady states which is computationally simple, and is especially useful for certain types of optimization problems. Instead of enzyme levels as parameters, we use the steady-state fluxes. Then, for each given steady state, metabolite levels $\mathbf{c}$ we can derive the enzyme levels, using the inverted kinetic rate laws discussed in the previous sections (essentially, the value of E in the EMC function). Therefore, we can redefine the set of steady states as $\mathcal{S} = \{(\mathbf{c}, \mathbf{v}, \boldsymbol{E}(\mathbf{v}, \mathbf{c}))\}_{\mathbf{v},\mathbf{c}}$ – where $\mathbf{c}$ and $\mathbf{v}$ correspond to the steady state values, like $\mathbf{c}(\infty)$ and $\mathbf{v}(\infty)$ in the previous definition. This representation of steady states has a number of practical advantages.

Proposition 1 Set of metabolic states *Consider a kinetic model with rate laws $v_l = E_l \, r_l(\mathbf{c})$, thermodynamically consistent rate constants (see SI 1.1), a feasible flux profile $\mathbf{v}$, and bounds on metabolite levels. For any feasible metabolite profile $\ln \mathbf{c} \in \mathcal{P}$ there is a unique set of enzyme levels E_l which realizes $\mathbf{c}$. The function $E_l(\ln \mathbf{c}) = v_l/r_l(\mathbf{c})$ is differentiable on the metabolite polytope.*

Proof: If a metabolite profile $\mathbf{c}$ is feasible for our flux profile $\mathbf{v}$, the catalytic rates $r_l(\mathbf{c})$ obtained from the rate laws Eq. (S1.1) must have the same signs as v_l, so $E_l = v_l/r_l(\mathbf{c})$ is positive on the entire metabolite polytope. In particular, we know that $\ln \mathbf{c} \in \mathcal{P} \rightarrow r_l(\mathbf{c}) \neq 0$. Since $r_l(\mathbf{c})$ is differentiable and has a constant sign on the

metabolite polytope, $E_l(\ln \mathbf{c})$ is differentiable on the metabolite polytope.

Here are some additional remarks.

- **Metabolite profiles parameterize the possible states** Proposition 1 guarantees that all thermodynamically feasible metabolite profiles can be realized by steady states of the kinetic model. In other words, the set $\mathcal{S}$ of metabolic states for a given flux profile $\mathbf{v}$ can be parameterized by the points of the metabolite polytope. This means that the set of kinetically realizable metabolite profiles in a kinetic model depends on the equilibrium constants, but not on other enzyme-specific parameters.

- **An enzyme profile need not uniquely determine the metabolite profile** In ECM, the same enzyme profile may be realizable by different metabolite profiles; this happens, in particular, if simplified rate laws are used. (i) If a metabolite appears in a model but has no impact on any reaction, its concentration can be freely varied, independently of fluxes or enzyme levels. (ii) With the EMC0 and EMC1 functions, enzyme levels do not depend on metabolite levels. (iii) With EMC2 functions, $\mathbf{s} = \ln \mathbf{c}$ can be varied along directions in the nullspace of $\mathbf{N}_{\mathrm{tot}}{}^{\top}$ without affecting the driving forces or enzyme cost. These EMC2 functions, on the metabolite polytope, have an invariant subspace (namely the nullspace of $\mathbf{N}_{\mathrm{tot}}{}^{\top}$). Under what conditions EMC3 and EMC4 functions (without regularization terms) have unique optima remains an open question.

3.2 Enzyme-based metabolic cost functions are convex on the metabolite polytope

The enzyme cost functions Eq. (6) are convex on the metabolite polytope: the cost for a metabolite log-concentration vector, interpolated between two vectors $\mathbf{s}_{\mathrm{a}}$ and $\mathbf{s}_{\mathrm{b}}$, cannot be higher than the interpolated cost:

$$\forall \lambda \in [0,1] : q(\lambda\, \mathbf{s}_{\mathrm{a}} + (1-\lambda)\, \mathbf{s}_{\mathrm{b}}) \leq \lambda\, q(\mathbf{s}_{\mathrm{a}}) + (1-\lambda)\, q(\mathbf{s}_{\mathrm{b}}). \tag{S1.18}$$

To show that all enzyme-based metabolic cost functions are convex, we consider the most general rate law with denominator (S1.3), written in factorized form

$$v \quad = \quad E \cdot k_{\mathrm{cat}}^{+} \cdot \eta^{\mathrm{rev}} \cdot \eta^{\mathrm{sat}}, \tag{S1.19}$$

where

$$\begin{aligned} \eta^{\mathrm{rev}} \quad &= \quad 1 - \mathrm{e}^{-\Theta} = 1 - \exp\left(\frac{1}{RT}\,\Delta_{\mathrm{r}} G^{\circ\prime} + \sum_i n_i \ln c_i\right) \\ \eta^{\mathrm{sat}} \quad &= \quad \prod_i \left(\frac{s_i}{K_i}\right)^{-m_i^{\mathrm{S}}} \left(\sum_k M_k \prod_j c_i^{m_{ik}}\right)^{-1} = \left(\sum_k \alpha_k \prod_j c_i^{a_{ik}}\right)^{-1} \end{aligned} \tag{S1.20}$$

with coefficients $\alpha_k \in \mathbb{R}_+$ and $a_{ik} \in \mathbb{R}$. The regulation factor η^{reg} need not be explicitly considered because it can be included in the term η^{sat}. With this rate law, the enzyme cost for a pathway reads

$$q_{\mathrm{pw}} = \sum_l q_l = \sum_l \frac{h_{E_l}\, v_l}{k_{\mathrm{cat},l}^{+}} \cdot \frac{1}{\eta_l^{\mathrm{rev}}} \cdot \frac{1}{\eta_l^{\mathrm{sat}}}. \tag{S1.21}$$

This function is convex on the metabolite polytope. For the proof, the cost function $h(\boldsymbol{E})$ need not be linear; if it is nonlinear, it must be convex. For the proof, we start with some general lemmas.

Lemma 1 *The function $f(y) = -\ln(1 - \mathrm{e}^{y})$ is convex in the range $y < 0$.*

Proof 3.1 *The second derivative*

$$\frac{d}{dy^2} f(y) = \frac{e^y}{(1-e^y)^2}$$

is positive for $y < 0$.

Lemma 2 *The function $f(\mathbf{s}) = \ln \sum_{k=1}^{n} e^{s_k}$ is convex.*

Proof 3.2

$$\nabla^2 f(\mathbf{s}) = \frac{\mathrm{Dg}(\mathbf{c})(\mathbf{1}^\top \mathbf{c}) - \mathbf{c}\,\mathbf{c}^\top}{(\mathbf{1}^\top \mathbf{c})^2} \qquad (\textit{where } c_i = e^{s_i})$$

$$\forall \mathbf{u}: \quad \mathbf{u}^\top \nabla^2 f(\mathbf{s}) \mathbf{u} = \frac{(\sum_i c_i u_i^2)(\sum_i c_i) - (\sum_i u_i c_i)^2}{(\sum_i c_i)^2} \geq 0$$

since $(\sum_i u_i\, c_i)^2 \leq (\sum_i c_i\, u_i^2)(\sum_i c_i)$ from the Cauchy-Schwarz inequality. Therefore, the Hessian $\nabla^2 f(\mathbf{s})$ is positive semi-definite, which proves that $f(\mathbf{s})$ is convex.

Lemma 3 *For any number $\nu \in \mathbb{R}_+$ and vector $\mathbf{n} \in \mathbb{R}^m$, the function $-\ln(1 - \nu\, e^{\mathbf{n} \cdot \mathbf{s}})$ is convex over $\{\mathbf{s} \in \mathbb{R}^m \mid \nu\, e^{\mathbf{n} \cdot \mathbf{s}} < 1\}$.*

Proof 3.3 *This function is a composition of $f = -\ln(1 - e^y)$ from Lemma 1 with the affine transformation $y = \mathbf{n} \cdot \mathbf{s} + \ln \nu$, an operation which preserves convexity.*

Lemma 4 *For any matrix $\mathbf{A} \in \mathbb{R}^{n \times m}$ and vectors $\mathbf{b} \in \mathbb{R}_+^n$, the following function is convex over $\mathbf{x} \in \mathbb{R}^m$:*

$$\ln \left(\sum_{k=1}^{n} e^{\mathbf{a}_k \cdot \mathbf{s} + b_k} \right) \tag{S1.22}$$

where $\mathbf{a}_i$ is the ith row of $\mathbf{A}$.

Proof 3.4 *This function is a composition of $f = \ln \sum_{i=1}^{n} e^{s_i}$ from Lemma 2 with the affine transformation $s_i = \mathbf{a}_i \cdot \mathbf{s} + b_i$, an operation which preserves convexity.*

Based on these lemmas, we can now prove the convexity of enzyme cost functions.

Lemma 5 *Assume that all enzyme-catalysed reactions in a model behave according to rate laws of the type*

$$v \;=\; E \cdot k_{\mathrm{cat}}^{+} \cdot \eta^{\mathrm{rev}} \cdot \eta^{\mathrm{sat}}, \tag{S1.23}$$

with η^{rev} and η^{sat} given by Eq. (S1.20), with coefficients $\alpha_k \in \mathbb{R}_+$ and $a_{ik} \in \mathbb{R}$. Assume that the enzyme cost function for enzymatic reaction l reads

$$q_l = \frac{h_{E_l}\, v_l}{E_l} = \frac{h_{E_l}\, v_l}{k_{\mathrm{cat},l}^{+}} \cdot \frac{1}{\eta_l^{\mathrm{rev}}} \cdot \frac{1}{\eta_l^{\mathrm{sat}}}\,. \tag{S1.24}$$

Then the total enzyme cost $q = \sum_l q_l$, as a function of logarithmic metabolite concentrations ($\mathbf{s} = \ln \mathbf{c}$), is convex.

Proof 3.5 *To simplify the efficiency factors, we can use the abbreviations $s_i \equiv \ln c_i$, $\nu \equiv \exp(\Delta_{\mathrm{r}} G^{\circ\prime}/RT)$, and*

$b_k = \ln \alpha_k$:

$$\begin{aligned} \eta^{\mathrm{rev}} &= 1 - \nu\, \mathrm{e}^{-\mathbf{n} \cdot \mathbf{s}} \\ \eta^{\mathrm{sat}} &= \left(\sum_{k=1}^{n} \mathrm{e}^{\mathbf{a}_k \cdot \mathbf{s} + b_k} \right)^{-1}. \end{aligned} \tag{S1.25}$$

If we look at the natural logarithm of q_l,

$$\ln q_l = \ln \left(\frac{h_{E_l}\, v_l}{k^{+}_{\mathrm{cat},l}} \right) - \ln \eta_l^{\mathrm{rev}} - \ln \eta_l^{\mathrm{sat}}, \tag{S1.26}$$

we see that each of the three terms in the sum is convex in $\mathbf{s}$. The first term is constant with respect to the metabolite concentrations and therefore trivially convex. The energetic term, $-\ln \eta^{\mathrm{th}} = -\ln(1 - \nu\, \mathrm{e}^{-\mathbf{n}\cdot\mathbf{s}})$, is convex according to Lemma 3. The saturation factor, $-\ln \eta^{\mathrm{sat}} = \ln \left(\sum_{k=1}^{n} \mathrm{e}^{\mathbf{a}_k \cdot \mathbf{s} + b_k} \right)$, is convex according to Lemma 4. We conclude that q_l is convex too, since it is a composition of a convex function (e^x) with another convex function ($\ln q_l$). Finally, the total enzyme cost (q) is convex since it is a sum of convex functions:

$$q = \sum_l q_l(\mathbf{s}). \tag{S1.27}$$

3.3 The ECM problem becomes strictly convex by adding a small metabolite cost as a regularization term

Along with the enzyme profiles, enzyme cost minimization also predicts metabolite profiles within predefined bounds. Since enzymes become more efficient (i.e., tend to show a higher flux per enzyme invested) at larger thermodynamic driving forces, the cell has an incentive to increase the substrate concentrations and to decrease the product concentrations in all reactions. At the same time, the optimization benefits from forcing enzymes into saturation, which leads to large metabolite levels in general. If we strictly minimize enzyme cost, we may obtain some extremely large (or small) metabolite levels, which hit the predefined concentration bounds, and the corresponding concentration/K_{M} ratios will become very high. To improve the metabolite predictions, we introduce a small penalty term that steers metabolite levels towards a typical concentration. As a typical concentration for metabolite i, we consider the center between the lower and upper concentration bound on logarithmic scale. To justify such a term, one may argue that large metabolite levels can be a burden for the cells, thus creating a tradeoff between enzyme efficiency (which can be improved by high metabolite levels) and metabolic burden (e.g., due to osmolarity, toxicity, loss by diffusion) [12, 13]. Thus, instead of the enzymatic metabolite cost $q(\mathbf{x}, \mathbf{v})$, we minimize the more general (enzymatic and metabolic) metabolite cost $q(\mathbf{x}, \mathbf{v}) + \lambda ||\mathbf{x} - \hat{\mathbf{x}}||$, where λ is a small, heuristically chosen prefactor (in our case, given by the median enzyme cost weight multiplied by 0.01). Since the enzyme term ist convex and the metabolite term is strictly convex, the resulting cost function is strictly convex. Therefore, even with arbitrarily small, positive values of λ, the ECM problem will have a unique solution.

3.4 The ECM problem remains convex under metabolite and enzyme constraints

In ECM, we may introduce an upper bound on the sum of all (non-logarithmic) metabolite levels as an extra constraint. Unlike our original metabolite polytope, the resulting admissible region will have a curved surface. However, since the sum of metabolite levels is convex on the metabolite polytope, the new constraint leads to a convex region, and the ECM problem remains convex. Another possible constraint comes from predefined concentrations of conserved moieties. A fixed concentration [ATP]+[ADP], for example, would define a nonlinear constraint on the metabolite polytope. However, the inequality constraint [ATP]+[ADP] $\leq \mathrm{const.}$ would lead to

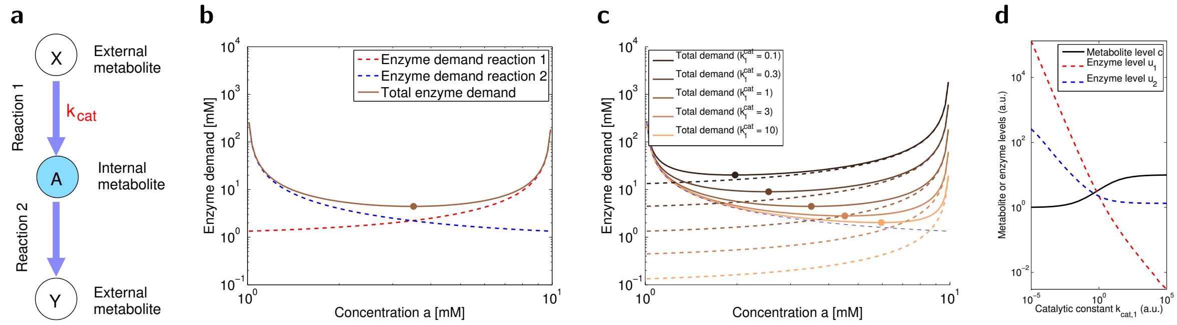

Figure B: **The optimal metabolic state depends on the catalytic constants.** (a) Two-reaction pathway with external concentrations $x = 1$ and $y = 0.1$. The enzyme demand depends on the concentration a of intermediate metabolite A. (b) Enzyme demand as a function of a, for a desired flux $v = 1$ (a.u.) and assuming reversible Michaelis-Menten rate laws (all parameters set to 1). Close to chemical equilibrium in reaction 1 (right border) or reaction 2 (left border), enzyme demand diverges. The optimum metabolite concentration is marked by a dot. (c) Enzyme demand depends on kinetic constants k^{+}_{cat}. Results with varying k^{+}_{cat} values in reaction 1 (reference value $k^{+}_{\mathrm{cat}} = 1$ s^{-1}) are shown. Higher k^{+}_{cat} values decrease the enzyme demand in reaction 1, shifting the optimum point towards higher values of a. (d) Optimal metabolite and enzyme levels shown as functions of k^{+}_{cat}.

a convex feasible region. Similarly, we may postulate that the sum of enzyme levels, or some weighted sums of enzyme levels (e.g., for enzymes occupying a certain membrane) are bounded from above. Since these sums are convex functions on the metabolite polytope, a bound on these sums will define a convex set, and again, the optimality problem remains convex.

3.5 Optimal metabolic states depend on model parameters.

How do optimal metabolite and enzyme profiles depend on kinetic parameters? The metabolite profile reflects a compromise between requirements in different reactions and depends on many model details. Changes in external concentrations or equilibrium constants will shift the boundaries of the metabolite polytope, and changes in k_{cat} values, enzyme cost weights h_{E_l}, or desired fluxes v_l will scale the cost of individual enzymes and shift the optimum point (see Figure 2 (f)). Figure B shows this for a varying k_{cat} value. We consider a two-reaction pathway with parameters set to 1 (arbitrary units). We note that a higher intermediate level a decreases the force in reaction 1 (i.e., increases its enzyme demand) and increases the force in reaction 2 (i.e., decreases its enzyme demand). With the parameters chosen, the total enzyme demand becomes minimal when both reactions show the same driving force: this is the state that would also be predicted by the MDF method (which focuses on driving forces instead of enzyme costs [14]). If we increase the k_{cat} value in reaction 1, the cost of enzyme 1 will have a smaller impact on the overall cost, and the optimal concentration a is shifted to higher values. Since the cost of enzyme 2 becomes more dominant, energy efficiency in this reaction is increased on the expense of reaction 1. A variation of enzyme cost weights h_{E_l} or fluxes v_l will have similar effects as variations of $1/k^{+}_{\mathrm{cat},l}$.

3.6 Enzyme uncertainties due to uncertain model parameters

Uncertainty ranges of enzyme levels, arising from uncertainties in model paramaters, are shown in Figure 5 in the main text. To quantify the uncertainties of all model parameters, we considered a multivariate Gaussian distribution for the vector of kinetic constants, on logarithmic scale. This distribution is directly obtained from parameter balancing as a posterior distribution. In a Monte Carlo sampling approach, parameter vectors were sampled and enzyme levels were numerically computed by running ECM for each sampled parameter set. However, for narrow parameter distributions, these multiple ECM runs can be avoided, and variances and covariances of

enzyme and metabolite levels can be computed directly based on a linear approximation of the kinetic rate laws. Except for the one ECM run with standard parameters, no further numerical optimization is required. This analytical approach is based on two assumptions. (i) Starting from the standard solution, any small change in an enzyme parameter will be compensated by adjusting the level of the same enzyme. This is an approximation: in reality, rerunning the numerical optimization would change all enzyme levels, but to very different extents. Since the changes in other enzymes are second-order effects, they can be ignored as long as parameter variations are small. (ii) If multiple parameters are changed at the same time, the effects of these changes are additive. Given these assumptions, a covariance matrix for the predicted enzyme profile can be computed as follows. We consider a joint variation of all kinetic constants in the model (vector $\Delta\mathbf{k}$). At constant enzyme and metabolite levels, this variation would lead to small relative flux changes $\Delta v_l/v_l$ in all reactions (with index l). In a linear approximation, the flux changes can be expressed as $\Delta v_l \approx \sum_p E_{lp}\,\Delta k_p$ with the parameter changes k_p and the parameter elasticity matrix $\mathbf{E}$, computed in the metabolic state with standard parameters. We now adjust all enzyme levels u_l to exactly compensate for the parameter changes, by setting $\Delta u_l = -u_l \frac{\Delta v_l}{v_l}$. Consequently, applying the parameter changes $\Delta\mathbf{k}$ at the same time as the enzyme level changes $\Delta\mathbf{u}$, would have not effect on the fluxes at all. This yields a linear relationship between a parameter variation $\Delta\mathbf{k}$ and the resulting variation of the optimal enzyme level (in matrix notation) $\Delta\mathbf{u} \approx -\mathrm{Dg}(\mathbf{u})\,\mathrm{Dg}(\mathbf{v})^{-1}\;\mathbf{E}\,\Delta\mathbf{k}$. From this relationship, and given a covariance matrix $\mathrm{cov}(\mathbf{k})$ of parameter changes $\Delta\mathbf{k}$, we obtain a formula for the covariance matrix of enzyme profiles

$$\mathrm{cov}(\mathbf{u}) = \mathrm{Dg}(\mathbf{u})\,\mathrm{Dg}(\mathbf{v})^{-1}\,\mathbf{E}\,\mathrm{cov}(\mathbf{k})\,\mathbf{E}^{\top}\,\mathrm{Dg}(\mathbf{v})^{-1}\,\mathrm{Dg}(\mathbf{u})\,. \tag{S1.28}$$

In this approximation, the average enzyme profile is still given by the enzyme profile for the standard parameter set. Note that we assumed, again, that local parameter variations are compensated by *local* enzyme adjustments. Local enzyme adjustments are directly and solely determined by the required flux, the given metabolite levels, and the parameter change, that is, by quantities within a single reaction. A *global* enzyme adjustment, in contrast, assumes that the metabolite levels are adjusted, too. By re-optimizing the metabolite levels, we also obtain different enzyme levels in the entire system. Our approximation formula (S1.28) relies on the fact that the adjustment of metabolite levels is a second-order effect. For small parameter variations, this adjustment is neglible, and local and global enzyme adjustments are approximately identical.

3.7 Preemptive enzyme expression as a convex optimality problem

Cells have to deal with varying environments which require different fluxes and enzyme levels. Since switching takes time and the resulting maladaptation can be costly, a possible strategy is to anticipate all possible (or likely) situations and to express enzymes preemptively. In a simple strategy, the cell could express all enzymes at a constant level and inhibit some of them in each situation to realise a favourable state. The choice of optimal preemptive enzyme levels can be formulated as an optimality problem, which turns out to be convex. We assume a set of possible situations σ, each characterised by different conditions (external metabolite concentration vector $\mathbf{c}^{\mathrm{ext},\sigma}$ and other kinetics-relevant parameters $\mathbf{p}^{\sigma}$) and a necessary flow $\mathbf{v}^{\sigma}$. For simplicity, we assume that each reaction has a fixed flux direction across all conditions. Each condition leads to a different metabolite polytope $\mathcal{P}^{\sigma}_{\mathbf{s}}$ and to a different specific rate function $r^{\sigma}(\mathbf{v},\mathbf{s})$. A preemptive adaptation strategy is a tuple $\{\mathbf{s}^{\sigma}\}$ of metabolite profiles for the different situations, each located in its metabolite polytope $\mathbf{s}^{\sigma} \in \mathcal{P}^{\sigma}_{\mathbf{s}}$. The corresponding *required enzyme activities* comprise the enzyme profiles required in the different situations:

$$E^{\sigma}_{l}(\mathbf{s}^{\sigma}) = \frac{v^{\sigma}_{l}}{r^{\sigma}_{l}(\mathbf{s}^{\sigma})} \tag{S1.29}$$

We note that each of the $E_l^\sigma(\mathbf{s}^\sigma)$ is a convex function on the corresponding metabolite polytope $\mathcal{P}_{\mathbf{s}}^\sigma$. To define the overall cost of the strategy, we determine, for each enzyme, the maximal level that it needs to show across situations (for all other situations, we assume that the enzyme activity will be reduced allosterically, without reducing the actual enzyme cost). Thus,

$$q^{\text{strategy}}(\{\mathbf{s}^\sigma\}) = \sum_l \max_\sigma E_l^\sigma(\mathbf{s}^\sigma). \tag{S1.30}$$

We now show that this cost is a convex function on the product polytope $\prod_\sigma \mathcal{P}_{\mathbf{s}}^\sigma$. First of all, the cost is convex if for each reaction l, the cost

$$q_l^{\text{strategy}}(\{\mathbf{s}^\sigma\}) = \max_\sigma E_l^\sigma(\mathbf{s}^\sigma) \tag{S1.31}$$

related to this reaction is convex. This is what we show now. From ECM, we know that $E_l^\sigma(\mathbf{s}^\sigma)$ is convex on $\mathcal{P}_{\mathbf{s}}^\sigma$, so

$$E_l^\sigma([1-\lambda]\,\mathbf{s}_A^\sigma + \lambda\,\mathbf{s}_B^\sigma) \leq [1-\lambda]\,E_l^\sigma(\mathbf{s}_A^\sigma) + \lambda\,E_l^\sigma(\mathbf{s}_B^\sigma). \tag{S1.32}$$

Thus,

$$\begin{aligned} q_l^{\text{strategy}}([1-\lambda]\,\{\mathbf{s}_A^\sigma\} + \lambda\,\{\mathbf{s}_B^\sigma\}) &= \max_\sigma E_l^\sigma([1-\lambda]\,\mathbf{s}_A^\sigma + \lambda\,\mathbf{s}_B^\sigma) \\ &\leq \max_\sigma\ ([1-\lambda]\,E_l^\sigma(\mathbf{s}_A^\sigma) + \lambda\,E_l^\sigma(\mathbf{s}_B^\sigma)) \\ &\leq [1-\lambda]\max_\sigma\ (E_l^\sigma(\mathbf{s}_A^\sigma)) + \lambda\max_\sigma\ (E_l^\sigma(\mathbf{s}_B^\sigma)) \\ &= [1-\lambda]\,q_l^{\text{strategy}}(\{\mathbf{s}_A^\sigma\}) + \lambda\,q_l^{\text{strategy}}(\{\mathbf{s}_B^\sigma\}) \end{aligned} \tag{S1.33}$$

This shows that $q_l^{\text{strategy}}(\{\mathbf{s}^\sigma\})$ is a convex function. The first inequality holds because $E_l^\sigma(\mathbf{s}^\sigma)$ is convex in $\mathbf{s}^\sigma$. the second inequality holds because the maximum function $\max(a_1, a_2, , a_3, ...)$ is convex in its arguments.

3.8 Non-enzymatic reactions

So far, we generally assumed that all reactions in a model are enzyme-catalysed. In reality, some chemical reactions are fast enough even without a catalyst, as is also the case for membrane diffusion (e.g., for small molecules like O_2 and CO_2). These processes are often counter-productive, such as spontaneous degradation of complex compounds or leakage efflux of useful metabolites. Furthermore, since many models of growing cells use metabolite concentrations (not absolute amount) as variables, the increase of cell volume dilutes these concentrations and is thus equivalent to a global degradation rate, which might be significant (e.g. in fast growing bacteria). These non-enzymatic processes could have a large impact on the metabolic flows or, in the perspective taken here, on how costly certain flows will be. Thus in general, our flows contain, aside from enzymatic reactions, a number of non-enzymatic reactions degrading or converting metabolites, most probably with mass-action rate laws. This radically changes things: in ECM, effectively, non-enzymatic reactions put additional constraints on metabolite concentrations, which confine the metabolite polytope to a subspace, and may make the polytope become empty.

In ECM, non-enzymatic reactions lead to constraints on the metabolite polytope, but leave the optimality problem convex Consider an ECM problem with non-enzymatic reactions. The rate laws $v_j^{\text{non}} = r_j(\mathbf{c})$ have the general form of reversible reactions (with thermodynamic numerator, and some concentration-dependent denominator), such that the functions $1/r_j(\mathbf{s})$ will be convex on the metabolite polytope. In ECM, with given fluxes, the metabolite levels must be such that the flux is realised. The non-enzymatic reactions are not scored by enzyme costs, but they create (potentially nonlinear) equality constraints on the metabolite polytope. In the

simple case of *irreversible mass action laws*, we obtain a linear equality constraint on the metabolite polytope, that is, the polytope is cut by a plane, and all solutions must lie in the resulting subspace. Obviously, this leaves the optimization problem convex.

3.9 The enzyme cost profile obtained by ECM is a linear combination of metabolic control profiles

In kinetic models of metabolic pathways, a flux maximization at a fixed total enzyme level will lead to a state in which enzyme levels and flux control coefficients are proportional [15]. Treating metabolic pathways by ECM, we obtain a similar, yet more general relationship between enzyme costs and metabolic control coefficients. The cost of an enzyme is proportional to a linear combination of metabolic control coefficients, and the control coefficients appearing in this linear combination refer to (i) possible stationary flux modes in the network and (ii) concentrations of internal metabolites that are either kept fixed or that hit a bound in ECM.

Proposition 2 (Enzyme cost and control coefficients) *In kinetic models with metabolic states obtained by enzyme cost minimization, the profile of enzyme cost $h_{E_l}\, E_l$ is a linear combination (proof see section 7.4)*

$$h_{E_l}\, E_l = \sum_{a \in \mathrm{stat}} \alpha_a \mathcal{C}_l^{j_a} + \sum_{b \in \mathrm{bnd}} \beta_b\, \mathcal{C}_l^{s_b}$$

of flux control coefficients (for the independent stationary fluxes j_a^{stat}) and of metabolite contral coefficients (for internal metabolites s_b^{bnd} that hit upper or lower bounds). The coefficients β_i assume values $\beta_i = 0$ when a metabolite hits none of the bounds, $\beta_i > 0$ when it hits the lower bound, and $\beta_i < 0$ when it hits the upper bound. In particular, if a network allows for a single stationary flux mode only, the enzyme costs show a proportionality

$$h_{E_l}\, E_l \propto \mathcal{C}_l^{\mathrm{J}} + \sum_{b \in \mathrm{bnd}} \beta'_b\, \mathcal{C}_l^{\mathrm{s_b}}. \tag{S1.34}$$

In the kinetic model, enzymes have no control over external metabolites. Thus, if all fixed metabolites (in ECM) are considered external (in the kinetic model), and if none of the internal metabolites (in the kinetic model) hits a bound (in ECM), the concentration control coefficients do not appear in the sum, then enzyme costs are directly proportional to flux control coefficients

$$h_{E_l}\, E_l \propto \mathcal{C}_l^{\mathrm{J}}. \tag{S1.35}$$

Assuming equal cost weights for all enzymes, this yields a proportionality between enzyme levels and flux control coefficients as previously shown in [15].

4 Reversibility-based cost functions and limits on driving forces

By studying the infeasible zones along polytope faces, we can derive tighter constraints on driving forces. The enzyme cost minimum can be inside the metabolite polytope or on a P-face (see Figure 2 (f)). Since enzyme costs rise fast near E-faces of the polytope, the optimum point will not be located in those regions, and it can be practical to exclude these regions from the metabolite polytope. This simply means that we introduce positive lower bounds on all driving forces. However, when should a driving force count as small? To define a threshold, we limit the enzyme cost in each reaction by an upper bound $q^{\max}$, e.g. five percent of the total cost of the proteome. We use the the general EMC equation (Eq. 6) to obtain a lower bound for driving forces (Θ_l), by

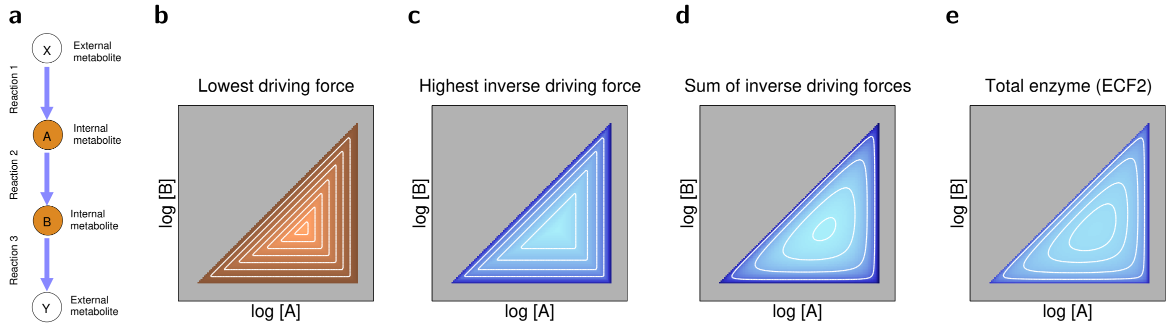

Figure C: **Estimates of enzyme cost, computed from driving forces.** The schemes show different possible cost functions for a three-reaction pathway – (a) is the same as in Figure 2. (b) Lowest driving force $\Theta^{\min} = \min_l \Theta_l$ along the pathway (colors from small (dark) to large (bright)). The contour lines can be used to define stricter constraints on metabolite profiles (see text). In the maximum point, all reactions have equal driving forces; this is the state that would follow from MDF optimization. (c) The highest inverse driving force in the pathway, ($\max_l \Theta_l^{-1}$), shows the same type of contour lines and the same optimum point (logarithmic color scale, from small (blue) to large (red)). (d) The sum $\sum_l \Theta_l^{-1}$ of inverse driving forces along the pathway. (e) The sum $\sum_l [1 - \mathrm{e}^{-\Theta_l}]^{-1}$ is an EMC2s function with all parameters set to 1. The function (c) is always lower than (d), (d) is lower than (e), and (e) is lower than an EMC3 function with parameters set to 1 (the one shown in Figure 2). All these functions have different optimum points.

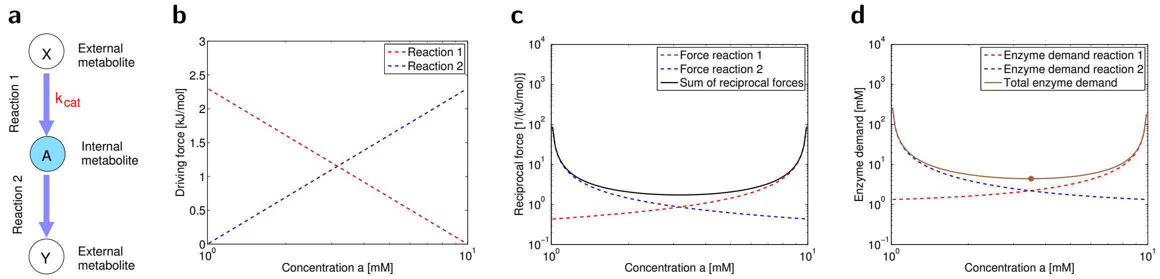

Figure D: **Two-reaction pathway with external concentrations** $x = 1$ **and** $y = 0.1$. The driving forces depend on the concentration a of intermediate metabolite A. (a) Driving forces as functions of a (note the logarithmic scale). (b) The inverse driving forces $1/\Theta_l$ and their sum are convex functions of $\log a$. As shown in Figure C, scaled inverse driving forces can be used as lower estimates of enzyme cost. (c) Enzyme demands for desired fluxe $v = 1$, assuming reversible Michaelis-Menten rate laws (all parameters set to 1). Close to chemical equilibrium in reaction 1 (right border) or reaction 2 (left border), the enzyme investment diverges. The optimum concentration is marked by a dot.

remembering that $\eta^{\mathrm{rev}} < 1$ and $\eta^{\mathrm{reg}} < 1$:

$$\frac{h_{E_l}\, v_l}{k^{+}_{\mathrm{cat},l}\, q^{\max}} < \frac{h_{E_l}\, v_l}{k^{+}_{\mathrm{cat},l}\, q_l} = \eta^{\mathrm{rev}} \eta^{\mathrm{sat}} \eta^{\mathrm{reg}} < \eta^{\mathrm{rev}} = 1 - e^{\Theta_l} < \Theta_l, \tag{S1.36}$$

where the last step uses the fact that $1 - \mathrm{e}^{x} < x$ for $x > 0$. The value varies between reactions (derivation in SI 7.1) and depends on fluxes, k_{cat} values, and enzyme cost weights h_{E} (where protein mass can be used as a proxy). With the constraint (S1.36), we obtain a smaller metabolite polytope in which the costly regions close to the previous E-faces are excluded. The same constraints can also be used in thermodynamics-based FBA. Usually, thermodynamics-based FBA requires that fluxes and driving forces have the same sign (e.g. [16, 17, 13]), but fluxes are allowed to be driven by infinitesimal forces. With our stricter (and more realistic) constraint, an FBA model would require, instead, that forces must be large enough to realize fluxes at plausible enzyme costs.

Other metabolite constraints, which ensure sufficient substrate levels, can be derived similarly. By putting an upper bound on the enzyme cost, we obtain a lower bound on the saturation efficiency η^{sat}, and thus on the

substrate levels. Consider, for instance, a reversible MM rate law for a reaction S $\rightleftharpoons$ P in the factorized form ($v = E k^{+}_{\rm cat} \eta^{\rm rev} \eta^{\rm sat} \eta^{\rm reg}$). Noting that $\eta^{\rm rev} < 1$ and $\eta^{\rm reg} < 1$, we obtain a lower bound on enzyme cost:

$$\frac{h_{E_l}\, v_l}{k^{+}_{{\rm cat},l}\, q^{\rm max}} < \eta^{\rm rev} \eta^{\rm sat} \eta^{\rm reg} < \eta^{\rm sat} = \frac{s/K_{\rm S}}{1 + s/K_{\rm S} + p/K_{\rm P}} < \frac{s}{K_{\rm S}}. \tag{S1.37}$$

For multi-substrate reactions, we obtain linear inequality constraints in log-concentration space. Using the EMC3s function, we obtain the cost estimate

$$q > q^{\rm min}_l = \frac{h\, v}{k^{+}_{\rm cat} \eta^{\rm sat}} > \frac{h\, v}{k^{+}_{\rm cat}} \frac{1 + \prod_i (s_i/K_i)^{m_i}}{\prod_i (s_i/K_i)^{m_i}} > \frac{h\, v}{k^{+}_{\rm cat}} \prod_i (K_i/s_i)^{m_i}, \tag{S1.38}$$

where s_i denotes substrate concentrations and m_i denotes substrate molecularities. With the upper bound $q < q^{\rm max}$, we obtain the constraint

$$\begin{aligned} \prod_i s_i^{m_i} &> \frac{h\, v \prod_i (K_i)^{m_i}}{k^{+}_{\rm cat}\, q^{\rm max}} \\ \Rightarrow \quad \sum_i m_i \ln s_i &> \ln \frac{h\, v \prod_i (K_i)^{m_i}}{k^{+}_{\rm cat}\, q^{\rm max}}. \end{aligned} \tag{S1.39}$$

Bounds for allosteric regulators (lower bounds for activators, upper bounds for inhibitors) are derived in a similar way. It is insightful to compare these formulae to the thermodynamics formula used in the mTOW approach for modelling of large metabolic systems [13]. Just like ECM, mTOW optimizes the metabolite levels while putting positive lower bounds on the thermodynamic forces: in the case of mTOW, all thermodynamic forces must be larger than a predefined positive value β (chosen to be 0.02, corresponding to a reaction Gibbs free energy of -0.05 kJ/mol). This is a heuristic assumption, which puts a limit on the relative backward flux in each reaction. Our Eq. (S1.36), which is enzyme-specific, can replace this uniform threshold and provides a direct link to enzyme cost and enzyme parameters. Another interesting point of comparison between mTOW and ECM is the choice of the cost function: while mTOW approximates enzyme costs by a fixed quadratic function (close to the thermodynamic threshold) or by zero values (far from the thermodynamic threshold), the EMC scores are enzyme-specific cost functions derived from rate laws and depending on kinetic parameters. In this sense, the heuristic formulae in mTOW can be seen as approximations of the kinetics-based ECM formulae.

Lower estimates of flux costs; an extension of the MDF strategy The total enzyme cost of a pathway, $q(\mathbf{s})$, can be a complicated function of the log-metabolite levels. However, simple functions can be used as lower bounds (see Figures C and D). First, in a pathway with N reactions, the total cost is always bounded by $N \min_l q_l(\mathbf{s})$ and $N \max_l q_l(\mathbf{s})$, i.e., N times the lowest or the highest enzyme cost in the pathway. Second, the simplified EMC functions yield lower estimates of the cost. By combining these arguments, we can justify the Max-min Driving Force strategy [14]. The MDF strategy is a heuristics for predicting the concentrations and driving forces in a pathway. It postulates that the smallest driving force in a pathway should be as large as possible. The MDF criterion is equivalent to minimizing $\max_l [1 - {\rm e}^{-\Theta_l}]^{-1}$, which is a lower bound on the EMC2s function $\sum_l [1 - {\rm e}^{-\Theta_l}]^{-1}$ with all constants set to 1. As shown in Fig. C (a) and (b), the MDF optimum is distant from the polytope E-faces and close to the minimum point of the EMC2 function. Thus, the MDF strategy avoids excessive enzyme costs that would occur at the polytope surface. As in Eq. (S1.36), one could devise a variant of MDF in which driving forces are weighted by the prefactors $\frac{k^{+}_{{\rm cat},l}}{h_{E_l}\, v_l}$.

5 Workflow for model building and metabolic optimization

5.1 Workflow description

Our algorithm for enzyme cost minimization has two main phases. In the kinetics phase, we collect and adjust the model parameters and construct a model with energetically consistent fluxes (exclusion of infeasible cycles) and rate constants (satisfying Haldane relationships and Wegscheider conditions). To determine consistent model parameters, the collected rate constants and equilibrium constants are adjusted and completed by parameter balancing.

1. Collect thermodynamic and kinetic data: standard chemical potentials μ°, equilibrium constants K_{eq}, Michaelis-Menten constants K_{M}, forward and reverse catalytic constants k^{+}_{cat} and k^{-}_{cat}.
2. Set some of these quantities to fixed values (if desired).
3. Run parameter balancing (with priors, pseudo values, and upper and lower bounds) to obtain a complete, consistent set of rate constants.

In the optimization phase, the desired pathway flux is realized by optimal enzyme and metabolite profiles.

1. Set up the kinetic model (based on the given network, flux profile $\mathbf{v}$, and model parameters). Redefine the reaction directions such that fluxes are positive, and update all parameters.
2. Choose the bounds for metabolite concentrations (tight bounds or fixed values for metabolites with fixed concentrations, lower and upper bounds for the others).
3. Determine a feasible metabolite profile $\mathbf{s} = \ln \mathbf{c}$ (a profile within the metabolite polytope) as a starting point for numerical optimization. We consider three alternatives: (i) Use linear programming to construct a set of extreme points in the polytope (with maximal and minimal metabolite levels s_i); the center of mass of these points is then taken as the starting point. (ii) Use the point in the polytope that is closest to the center of the predefined metabolite bounds (solution of a quadratic programming problem) as the starting point. (iii) Use the solution of the MDF problem (linear programming problem) as the starting point.
4. Choose an EMC function and minimize it numerically with respect to $\mathbf{s}$ under the constraints defining the metabolite polytope.
5. Compute the corresponding enzyme levels and cost.
6. Based on the optimal enzyme cost, define a maximal tolerable cost (e.g., one percent higher than the optimal total cost) and compute individual tolerances for metabolite and enzyme levels as described in Methods.
7. Validate the predicted enzyme and metabolite levels with experimental data.

In theory, a convex optimization should converge without problems. As a check, we can repeat the calculation with different starting points.

5.2 Parameter balancing yields consistent rate constants

For our kinetic models, we need consistent sets of rate constants (k^{+}_{cat}, K_{eq}, and K_{M} values) satisfying Wegscheider conditions and Haldane relationships (see SI 1.1). Measured parameter values may be incomplete and contradictory. Using parameter balancing [18], we can translate such values into complete, consistent, and plausible

parameters for a given model, and we can quantify the uncertainty of these values by computing a joint probability distribution for the entire set of kinetic model parameters. This distribution reflects, first, measured kinetic and thermodynamic constants as well as their measurement errors; second, prior distributions for parameter types (e.g., mean values and a standard deviation for logarithmic K_{M} values in general); and, third, known dependencies between different parameters, arising froim thermodynamic Wegscheider conditions and Haldane relationships.

Parameter balancing works as follows. We collect all quantities that appear in the data or in the model ($\ln k^{+}_{\mathrm{cat}}$, $\ln k^{-}_{\mathrm{cat}}$, $\ln K_{\mathrm{eq}}$, $\ln K_{\mathrm{M}}$, $\Delta_{\mathrm{r}}G^{\circ\prime}$, μ°) and merge them into a vector $\mathbf{y}$. These quantities must satisfy Wegscheider conditions and Haldane relationships, which defines linear equality constraints between them. Accordingly, to satisfy the constraints in a safe way, we write all these quantities as linear combinations of independent parameters ($\ln k^{\mathrm{V}}$, $\ln K_{\mathrm{M}}$, and μ° values), whith the definition $k^{\mathrm{V}} = \sqrt{k^{+}_{\mathrm{cat}}\, k^{-}_{\mathrm{cat}}}$. The independent parameters, which are collected in a vector $\mathbf{s}$, can be varied without violating any constraints. The linear dependence between the complete and the independent parameter sets can be written as $\mathbf{y} = \mathbf{R}\,\mathbf{s}$ with a matrix $\mathbf{R}$ derived from the model structure. Using this equation as a linear regression model, we can convert an experimentally known vector $\mathbf{y}^{\mathrm{data}}$ (which may be incomplete) into a best estimate of the underlying vector $\mathbf{s}$. Using the estimate $\mathbf{s}$, we again apply $\mathbf{R}$ to obtain a completed, consistent version of $\mathbf{y}$. Since this regression problem is usually underdetermined, we employ Bayesian estimation. Priors allow us to obtain plausible estimates even from sparse data. Accordingly, the result is not simply a point estimate of $\mathbf{y}$, but a multivariate Gaussian posterior distribution for possible parameter vectors $\mathbf{y}$. A best estimate is given by the center of the distribution; from the covariance matrix, we obtain uncertainties of individual model parameters as well as the correlations between them. Parameter balancing can handle data of different amounts or quality. If comprehensive data are available, they will just be adjusted to satisfy the constraints; missing or uncertain data values will be completed with plausible values. MATLAB code for parameter balancing and hyperparameters specifying the prior distributions are provided on github and www.metabolic-economics.de/enzyme-cost-minimization/.

5.3 Possible modifications of the workflow

The workflow can be extended in a number of ways:

- **External/internal and fixed/variable metabolites.** In kinetic models, we distinguish between *internal* metabolites, for which a mass balance must be satisfied within the model, and *external* metabolites, for which no mass balance is required (possibly assuming that other reactions, outside the model, will fix the mass balance). In ECM, we distinguish between fixed metabolites (whose concentration is predefined) and variable metabolites (whose concentration is determined during ECM). It is important to note that the two distinctions need not coincide. Nevertheless, metabolites at the pathway boundaries (such as initial substrates, final products, and cofactors, which also participate in other pathways) are usually the ones that will be both external (in kinetic models) and fixed (in ECM).

- **Fluxes need not be stationary.** The flux distribution used in ECM need not be stationary (in the sense that the variable metabolites satisfy mass balances). Remember that variable metabolites and internal metabolites are not the same! Of course, stationarity is a sensible assumption for whole-metabolism models on a certain timescale. However, fluxes that look stationary on the entire metabolic network may not look stationary on an individual pathway model (because there may be side reactions that fix the mass balances, but do not appear in the model).

- **Inactive reactions.** Inactive reactions (with a reaction flux $v_l = 0$) do not entail any energetic constraints or enzyme costs and can therefore by ignored. In constrast, if a driving force is known to vanish, this should be used as a constraint on the metabolite levels.

- **Non-enzymatic reactions.** If non-enzymatic reactions (typically with mass-action rate laws) are included in the optimality problem, they contribute to the energetic constraints, but not to the enzyme cost function.

- **Spatial structure.** ECM applies to compartment models, in which metabolites can have different concentrations in different compartments. Other spatial effects, such as substrate channeling, are ignored. To account for substrate channeling, the increased substrate concentration at enzymes' catalytic sites could be modelled, approximately, by using effective rate constants.

- **Constraints on the sum of metabolite levels or sums of enzyme levels** In addition to our bounds on individual metabolite levels, we can also set a bound on the total (non-logarithmic) metabolite concentration in the cell [13]. The resulting ECM problem remains convex (see section 3.4). Alternatively, one could penalize large total concentrations by subtracting a concave function $R(\sum_i c_i)$ from the enzyme cost; in log-concentration space, this would yield a convex cost term. The same holds for constraints on the sums of some enzyme levels.

- **Enzyme demand and cost per flux.** Under the assumptions made (linear enzyme cost function; fixed external metabolite levels in kinetic model), enzyme demand and cost scale proportionally with the pathway fluxes. This holds for all EMC functions, but not for the MDF score [14], which remains constant under a proportional scaling of pathway fluxes[7].

- **Constraints on concentrations** Constraints on metabolite levels can be justified as follows. Upper bounds may reflect the fact that space in cells is limited, and physiological concentration ranges for certain compounds may be known from experience. Some metabolites may have high or low levels for specific reasons: for instance, yeast cells (and also Dunaliella algae) use high glycerol concentrations to balance high external salinity; other metabolites may be toxic in higher concentrations. Lower bounds are important when using the EMC2 functions, because these functions favor low product levels while lacking the saturation factor, which prevents very low substrate levels.

- **Values and uncertainties of rate constants** Different EMC functions require different types of rate constants for their calculation. All functions require forward catalytic constants k^{c}_{+}; EMC2 and higher functions require equilibrium constants, EMC3 or higher functions require Michaelis-Menten constants. Many rate constants are unknown and need to be estimated. To determine the rate constants for our calculations, we collect known kinetic data and convert them into complete, consistent parameter sets by parameter balancing [18, 19]. Parameter balancing yields a joint distribution of all model parameters describing their individual uncertainties and correlations. A consistent, most likely set of parameters follows from the median values of the marginal distributions. By sampling parameters from their joint distribution, we can obtain an ensemble of model variants with different consistent parameter sets. By running ECM for many such model variants, we can study how uncertainties in the rate constants affect the end result.

- **Sampling of nearly optimal solutions** Deviations from the optimum metabolite profile lead to a fitness loss. For small Gaussian random deviations, the average loss by can be computed by $\mathrm{Tr}(\mathrm{cov}(\mathbf{s})^{-1})\,\mathbf{H}_q$, where $\mathbf{s} = \ln \mathbf{c}$, $\mathrm{cov}(\mathbf{s})$ is the covariance matrix of metabolite log-concentrations, and $\mathbf{H}_q$ is the Hessian matrix of the (non-logarithmic) cost function $q(\mathbf{s})$ in the optimum point. To estimate the metabolite covariance matrix, we make an assumption inspired by statistical thermodynamics: we postulate that the relative probabilities of two metabolite vectors is given by $\frac{\mathrm{prob}(\mathbf{s}_1)}{\mathrm{prob}(\mathbf{s}_2)} = e^{-(q(\mathbf{s}_1)-q(\mathbf{s}_2))/q_0}$, where q_0 defines a scale of tolerable fitness deviations. The metabolite covariance matrix follows directly as $\mathbf{C} = q_0\,\mathbf{H}_q^{-1}$. For the reversibility-based EMC2s function, the Hessian matrix in the optimum point can be computed analytically at least.

[7] The linear scaling of enzyme levels holds only if all fluxes are scaled proportionally. In branched pathways, a non-proportional scaling would change the flux branching ratios. The resulting changes in the optimal concentrations at the branch points would change the enzyme cost in complicated ways.

Data type	Unit	Provenance	Reference
Reaction Gibbs energies	kJ/mol	Component contribution	[20]
Catalytic constants (k_{cat})	1/s	BRENDA	[21]
Michaelis-Mention constants (K_{M})	mM	BRENDA	[21]
Fluxes	mM/s	van Rijsewijk *et al.* [a]	[22]
Metabolite levels[b]	mM	Gerosa *et al.* [a]	[23]
Enzyme levels[b]	mM	Schmidt *et al.* [a]	[24]
Protein lengths	AAs	`www.uniprot.org`	

Table B: Data used in construction of *E. coli* model by ECM. Processed data can be found at www.metabolic-economics.de/enzyme-cost-minimization/. Units refer to preprocessed data. [a] Specific data corresponding to wild-type *E. coli* BW25113, grown in batch culture on minimal media (M9) and glucose. [b] Data used for validation only.

- **Tolerable deviations of metabolite and enzyme levels** Tolerance ranges of metabolite or enzyme levels can be obtained by minimizing or maximizing these levels under the constraints used in ECM, plus the constraint that the cost must remain below some predefined upper bound. To speed up the calculation, an approximation based on the Hessian matrix of the logarithmic cost function can be used (see SI 7.3). Alternatively, we could sample metabolite profiles with enzyme costs close to the optimum. Using the Metropolis-Hastings algorithm, we could obtain an ensemble of metabolite and enzyme profiles, where less costly states appear with higher probabilities (see SI 5.1).
- **Lumped reactions** To simplify models, pathways can be lumped into single reactions (for parameter choices, see SI 7.2). The lumping of reactions resembles the way in which ECM, altogether, attributes enzyme costs or specific activities to entire pathways.

6 Model of central metabolism in E. coli

Our *E. coli* model was built from a list of chemical reactions in central metabolism as given by KEGG. KEGG reaction identifiers [25] were automatically translated into a kinetic model; compounds and reactions are denoted by KEGG identifiers, and genes are denoted mostly by their common names in *E. coli*. The cofactors ATP, ADP, phosphate, NADH, NAD^+, NADPH, and $NADP^+$ are included in the model. During ECM, all metabolite levels were limited to predefined ranges, and the levels of cofactors and some other metabolites were fixed at experimentally known values. Kinetic and thermodynamic constants, as well as state-dependent data, were determined based on published measurement values. Equilibrium constants were estimated using the component contribution method [20], kinetic constants (k^+_{cat} and K_{M} values) were obtained from the BRENDA database (after which each value was curated manually), and a complete, globally consistent parameter set was determined by parameter balancing. State-dependent data were obtained from publications using batch fed *E. coli* BW25113 grown on minimal media (M9) with glucose as the carbon source. Our source for metabolic fluxes [22] used ^{13}C metabolic flux analysis, metabolite concentrations [23] were obtained using LC-MS/SM, and enzyme concentrations [24] using SWATH-MS. For a summary of data provenance, see Table B. All data sources are listed in Table B, and models and data are provided at `www.metabolic-economics.de/enzyme-cost-minimization/`. Figure E shows the correlations between predicted and measured metabolite levels, corresponding to the enzyme predictions in Figure 3. More details can be found on `www.metabolic-economics.de/enzyme-cost-minimization/`.

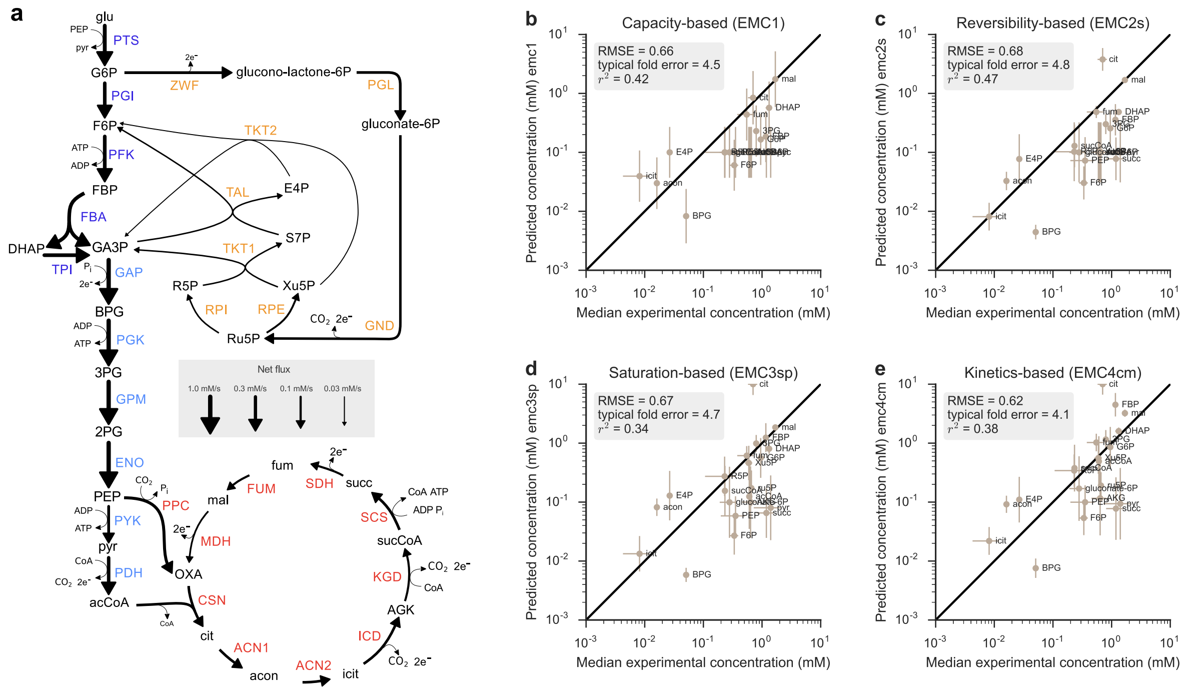

Figure E: **Metabolite levels predicted by enzymatic metabolic cost minimization.** As in Figure 3 for enzyme predictions, vertical bars denote tolerance ranges. Horizontal lines represent uncertainties in measured data. Predictions are based on fluxes from [22], k^{+}_{cat} and K_{M} values from BRENDA [21], and validated with metabolite concentrations from [23].

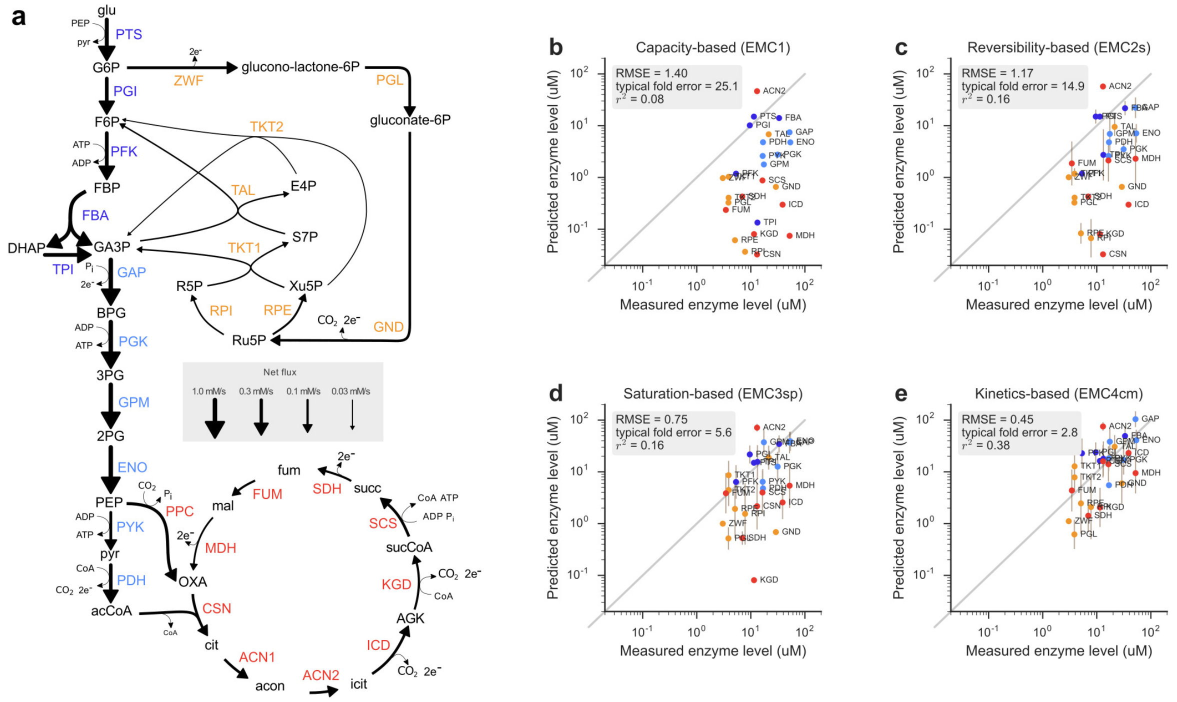

Figure F: **Enzyme levels predicted by enzymatic metabolic cost minimization.** Predictions are exactly the same as in Figure 3, but measured values (abscissa) are taken from [26] instead of [24].

7 Proofs and derivations

7.1 Lower bounds on driving forces, Eq. (S1.36)

Assuming that the cost for an individual enzyme cannot exceed a certain limit $q_l < q^{\max}$, we obtain Eq. (S1.36) in section 4 as a lower bound on the driving forces: $\Theta_l > \frac{h_{E_l}\, v_l}{k^+_{\mathrm{cat},l}\, q^{\max}}$. Noting that $\Theta = -\Delta_{\mathrm{r}} G'/RT$, we get that the reaction Gibbs energies are bounded by

$$\Delta_{\mathrm{r}} G'_l \quad < \quad -RT\, \frac{h_{E_l}\, v_l}{q^{\max}\, k^+_{\mathrm{cat},l}}. \tag{S1.40}$$

7.2 Parameters for lumped reactions

A lumped reaction describes a series of reactions as if they were catalyzed by a single enzyme. The kinetic parameters should agree with the original catalytic constants $k^+_{\mathrm{cat},l}$, enzyme levels E_l, and enzyme cost weights h_{E_l} of the individual reactions and yield the right pathway flux $v = E\, k^+_{\mathrm{cat}}$ and the right enzyme cost $q = h\, E$, but this still leaves some freedom of choice. On the one hand, we may assume that our hypothetical lumped enzyme resembles a normal enzyme in its kinetics and concentration. This can be realized in different ways:

- Set $k_{\mathrm{cat}} = \langle k^+_{\mathrm{cat},l} \rangle_{\mathrm{geom}}$. To satisfy $v = v_l = k^+_{\mathrm{cat},l}\, E_l = \langle k^+_{\mathrm{cat},l}\, E_l \rangle_{\mathrm{geom}} = \langle k_{\mathrm{cat}} \rangle_{\mathrm{geom}} \langle E_l \rangle_{\mathrm{geom}}$, we must set $E = \langle E_l \rangle_{\mathrm{geom}}$.
- Set $k_{\mathrm{cat}} = \langle k^+_{\mathrm{cat},l} \rangle_{\mathrm{arith}}$. To satisfy $v = \langle k^+_{\mathrm{cat},l}\, E_l \rangle_{\mathrm{arith}} = \langle k_{\mathrm{cat}} \rangle_{\mathrm{arith}} \langle \frac{k^+_{\mathrm{cat},l}}{k_{\mathrm{cat}}} E_l \rangle_{\mathrm{arith}}$, we must set $E = \langle \frac{k^+_{\mathrm{cat},l}}{k_{\mathrm{cat}}} E_l \rangle_{\mathrm{arith}}$.
- Set $E = \langle E_l \rangle_{\mathrm{arith}}$. Again, we must set $k_{\mathrm{cat}} = \langle \frac{E_l}{E} k^+_{\mathrm{cat},l} \rangle_{\mathrm{arith}}$.

In all three cases, the identity $h\, E = \sum_l h_{E_l}\, E_l$ leads to the formula $h = \sum_l h_{E_l} \frac{E_l}{E}$ for specific cost. Since a lumped enzyme represents several real enzymes, it will appear more costly or "larger". On the other hand, we can assume that the concentration of the lumped enzyme is given by the sum of original enzyme concentrations; this implies smaller effective k_{cat} values. To obtain the parameters, we can use the previous formulae and replace $E \to n\, E$, $h \to h/n$, and $k_{\mathrm{cat}} \to k_{\mathrm{cat}}/n$.

7.3 Tolerance intervals around the minimum point of a strictly convex function

Consider a strictly convex function $f(\mathbf{s})$ with a global minimum $\mathbf{s}^*$. Due to strict convexity, the Hessian $\mathbf{H}(\mathbf{s})$ is a positive definite matrix. To calculate tolerance intervals around the minimum point, we choose the tolerance threshold τ (e.g. 1% of the minimum value) and define the tolerance subspace:

$$S_{\mathrm{tol}} \equiv \{\mathbf{s} \mid f(\mathbf{s}) < f(\mathbf{s}^*) + \tau\}. \tag{S1.41}$$

To get an explicit formula for S_{tol}, we first approximate f around its minimum point by a Taylor expansion:

$$f(\mathbf{s}^* + \boldsymbol{\xi}) = f(\mathbf{s}^*) \;+\; \nabla f(\mathbf{s}^*)^\top \boldsymbol{\xi} \;+\; \frac{1}{2} \cdot \boldsymbol{\xi}^\top \mathbf{H}(\mathbf{s}^*) \boldsymbol{\xi} \;+\; \ldots \tag{S1.42}$$

In the minimal point, $\nabla f(\mathbf{s}^*) = 0$ holds and we drop the extra terms in the Taylor expansion to get

$$f(\mathbf{s}^* + \boldsymbol{\xi}) = f(\mathbf{s}^*) \;+\; \frac{1}{2} \cdot \boldsymbol{\xi}^\top \mathbf{H} \boldsymbol{\xi} \tag{S1.43}$$

(for convenience, we use $\mathbf{H}$ to refer to the Hessian at the optimum). Therefore, the tolerance region can be approximated by:

$$S_{\text{tol}} \approx E_{\text{tol}} \equiv \{\mathbf{s}^* + \boldsymbol{\xi} \mid 2\tau > \boldsymbol{\xi}^\top \mathbf{H}\boldsymbol{\xi}\} \tag{S1.44}$$

Lemma 6 *If we define the ellipsoid* $E \equiv \{\mathbf{H}^{-\frac{1}{2}}\mathbf{y} \mid \mathbf{y}^\top\mathbf{y} < 1\}$*, then*

$$E_{\text{tol}} = \mathbf{s}^* + \sqrt{2\tau} \cdot E$$

Proof 7.1 *Since* $\mathbf{H}$ *is symmetric and positive definite, it is invertible and thus* $\mathbf{H}^{-\frac{1}{2}}$ *is a unique symmetric matrix. Any* $\mathbf{s} \in \mathbf{s}^* + \sqrt{2\tau} \cdot E$ *can be written as* $\mathbf{s} = \mathbf{s}^* + \boldsymbol{\xi}$*, where* $\boldsymbol{\xi} = \sqrt{2\tau} \cdot \mathbf{H}^{-\frac{1}{2}}\mathbf{y}$ *and* $\mathbf{y}^\top\mathbf{y} < 1$*, therefore*

$$\boldsymbol{\xi}^\top \mathbf{H}\boldsymbol{\xi} = \sqrt{2\tau} \cdot \mathbf{y}^\top \mathbf{H}^{-\frac{1}{2}}\mathbf{H}\mathbf{H}^{-\frac{1}{2}}\mathbf{y} \cdot \sqrt{2\tau} = 2\tau \cdot \mathbf{y}^\top\mathbf{y} < 2\tau. \tag{S1.45}$$

The reverse direction follows trivially. □

Corollary 7.2 *An ellipsoid is not always a convenient shape for describing the tolerance intervals because there is dependence between the different dimensions. For some application, it is sufficient to consider the bounding box of* E*, which is given by* $B \equiv \{\mathbf{D}\mathbf{y} \mid \mathbf{y} \in [-1, 1]^n\}$*, where* $\mathbf{D}$ *is a matrix containing only the diagonal values in* $\mathbf{H}^{-\frac{1}{2}}$ *(i.e.* $\mathbf{D}_{ii} = \sqrt{(\mathbf{H}^{-1})_{ii}}$*). Then we can approximate* S_{tol} *by*

$$S_{\text{tol}} \approx \mathbf{s}^* + \sqrt{2\tau} \cdot B \tag{S1.46}$$

Therefore, for a single dimension i *the tolerance interval will be described by*

$$x_i^* \pm \sqrt{2\tau(\mathbf{H}^{-1})_{ii}} \tag{S1.47}$$

7.4 Enzyme costs reflects metabolic control (proposition 2)

Consider the ECM problem

$$\text{Minimize} \quad h(\boldsymbol{E}) \qquad \text{subject to} \quad \mathbf{j}_{\text{stat}}(\boldsymbol{E}) = \mathbf{v}_{\text{stat}},\ \mathbf{s}_{\text{bound}}(\boldsymbol{E}) = \mathbf{c}_{\text{bound}},$$

where “stat” refers to independent stationary fluxes (with running index a) and “bound” refers to metabolites that hit a bound in the ECM solution considered (index b). With Lagrange multipliers λ_a and μ_b for the two sorts of constraints, the optimality condition reads

$$0 = \frac{\partial h}{\partial E_l} + \sum_{a \in \text{stat}} \lambda_a \frac{\partial j_a}{\partial E_l} + \sum_{b \in \text{bnd}} \mu_b \frac{\partial s_b}{\partial E_l}.$$

After defining the enzyme cost slopes $h'_{E_l} = \frac{\partial h}{\partial E_l}$ and multiplying the equation by E_l, we obtain

$$0 \;=\; h'_{E_l}\, E_l + \sum_{a \in \text{stat}} \lambda_a \frac{\partial j_a}{\partial E_l} E_l + \sum_{b \in \text{bnd}} \mu_b \frac{\partial s_b}{\partial E_l} E_l.$$

We can now rewrite this in terms of control coefficients. The control coefficients between enzymes and independent stationary fluxes are defined by $\mathcal{C}^{j_a}_l = \frac{E_l}{j_a}\frac{\partial j_a}{\partial E_l}$, and those between enzymes and constrained metabolites are defined by $\mathcal{C}^{s_b}_l = \frac{E_l}{s_b}\frac{\partial s_b}{\partial E_l}$. Inserting this, we obtain

$$0 \;=\; h'_{E_l}\, E_l + \sum_{a \in \text{stat}} -\alpha_a \mathcal{C}^{j_a}_l + \sum_{b \in \text{bnd}} -\beta_b\, \mathcal{C}^{s_b}_l,$$

where we have defined $\alpha_a = -\lambda_a\, j_a$ and $\beta_b = -\mu_b\, s_b$, and thus

$$h'_{E_l}\, E_l \quad = \quad \sum_{a \in \mathrm{stat}} \alpha_a \mathcal{C}^{j_a}_l + \sum_{b \in \mathrm{bnd}} \beta_b\, \mathcal{C}^{s_b}_l.$$

These relations hold for general non-linear cost function. In the case of linear cost functions $h(\boldsymbol{E}) = \sum_l h_{E_l}\, E_l$ (as usually assumed in ECM), the enzyme cost slopes h'_{E_l} are directly given by the cost weights h'_{E_l}.

8 Mathematical symbols

Rate laws	Symbol	Units
Flux	v_l	mM/s
Metabolite level	c_i	mM
Enzyme level	E_l	mM
Rate law	$v_l(E_l, \mathbf{c}) = E_l \cdot r_l(\mathbf{c})$	mM/s
Catalytic rate	$r_l = v_l/E_l$	1/s
Gibbs energy of formation (standard chem. pot.)	$G_i'^{\circ}$	kJ/mol
Reaction Gibbs energy	$\Delta_{\rm r}G'_l = \Delta_{\rm r}G_l'^{\circ} + RT\sum_i n_{il} \ln c_i$	kJ/mol
Thermodynamic driving force	$\Theta_l = -\Delta_{\rm r}G'_l/RT$	unitless
**Kinetic models**		
Forward/backward catalytic constant	$k^{+}_{\rm cat}, k^{-}_{\rm cat}$	1/s
Michaelis-Menten constant	K_{li}	mM
Hill-like coefficient	γ_l	unitless
Molecularity for substrate (S) or product (P)	$m^{\rm S}_{li}$, $m^{\rm P}_{li}$	unitless
Regulation coefficient (activator A or inhibitor I)	$m^{\rm A}_{li}, m^{\rm I}_{li}$	unitless
Scaled reactant elasticity	$\mathcal{E}_{li} = \frac{c_i}{v_l}\frac{\partial v_l}{\partial c_I}$	unitless
Scaled flux control coefficient	$\mathcal{C}^{j_a}_l$	unitless
Scaled concentration control coefficient	$\mathcal{C}^{s_b}_l$	unitless
**Enzyme costs**		
Enzyme cost	$h_l(E_l) = h_{E_l}\, E_l$	D
Enzyme cost weight	h_{E_l}	D/mM
Protein mass	m_l	Da
Enzyme-based metabolic cost	$q(\mathbf{s}) = \sum_l q_l(\mathbf{s}) = \sum_l h_{E_l}\, E_l(\mathbf{s}, \mathbf{v})$	D
Hessian matrix of enzyme-based metabolic cost	$\mathbf{H}_q$	D
Flux-specific cost	$a_{v_l} = q_l/v_l = h_{E_l}/r_l$	D/(mM/s)
Baseline flux cost	$a^{\rm cat}_{v_l}$	D/(mM/s)
**Metabolic pathways**		
Pathway flux (flux in representative reaction)	$v_{\rm pw}$	mM/s
Scaled flux	$v'_l = v_l/v_{\rm pw}$	unitless
Flux-specific cost	$a^{\rm pw}_{\rm v} = \sum_l h_{E_l}\, E_l/v_{\rm pw} = \sum_l v'_l\, a_{v_l}$	D s/mM

Table C: Mathematical symbols used in ECM. Darwin (D) is a hypothetical fitness unit replacing the possible fitness units in different models. Reaction orientations are defined in such a way that fluxes are positive. Fluxes are given in units of concentration per time, but could also be given as amounts per time (e.g., mol/s); the latter choice is more practical for models with transport reactions.

References

[1] W. Liebermeister, J. Uhlendorf, and E. Klipp. Modular rate laws for enzymatic reactions: thermodynamics, elasticities, and implementation. *Bioinformatics*, 26(12):1528–1534, 2010.

[2] J.B.S. Haldane. *Enzymes*. Longmans, Green and Co., London. (republished in 1965 by MIT Press, Cambridge, MA), 1930.

[3] R. Wegscheider. Über simultane Gleichgewichte und die Beziehungen zwischen Thermodynamik und Reactionskinetik homogener Systeme. *Z. Phys. Chem.*, 39:257–303, 1902.

[4] J.S. Hofmeyr, O.P.C. Gqwaka, and J.M. Rohwer. A generic rate equation for catalysed, template-directed polymerisation. *FEBS Letters*, 587:2868–2875, 2013.

[5] W. Liebermeister and E. Klipp. Bringing metabolic networks to life: convenience rate law and thermodynamic constraints. *Theor. Biol. Med. Mod.*, 3:41, 2006.

[6] K. Zhuang, G.N. Vemuri, and R. Mahadevan. Economics of membrane occupancy and respiro-fermentation. *MSB*, 7:500, 2011.

[7] K.A. Dill, K. Ghosh, and J.D. Schmit. Physical limits of cells and proteomes. *PNAS*, 108(44):17876–17882, 2011.

[8] M. Eames and T. Kortemme. Cost-benefit tradeoffs in engineered lac operons. *Science*, 336:911–915, 2012.

[9] H. Akashi and T. Gojobori. Metabolic efficiency and amino acid composition in the proteomes of Escherichia coli and Bacillus subtilis. *PNAS*, 99(6):3695–3700, 2002.

[10] I. Shachrai, A. Zaslaver, U. Alon, and E. Dekel. Cost of unneeded proteins in E. coli is reduced after several generations in exponential growth. *Molecular Cell*, 38:1–10, 2010.

[11] A. Bar-Even, E. Noor, N.E. Lewis, and R. Milo. Design and analysis of synthetic carbon fixation pathways. *PNAS*, 107(19):8889–8894, 2010.

[12] S. Schuster and R. Heinrich. Minimization of intermediate concentrations as a suggested optimality principle for biochemical networks. *Journal of Mathematical Biology*, 29(5):425–442, 1991.

[13] N. Tepper, E. Noor, D. Amador-Noguez, H.S. Haraldsdóttir, R. Milo, J. Rabinowitz, W. Liebermeister, and T. Shlomi. Steady-state metabolite concentrations reflect a balance between maximizing enzyme efficiency and minimizing total metabolite load. *PLoS ONE*, 8(9):e75370, 2013.

[14] E. Noor, A. Bar-Even, A. Flamholz, E. Reznik, W. Liebermeister, and R. Milo. Pathway thermodynamics uncovers kinetic obstacles in central metabolism. *PLoS Comp. Biol.*, 10:e100348, 2014.

[15] R. Heinrich and E. Klipp. Control analysis of unbranched enzymatic chains in states of maximal activity. *J. Theor. Biol.*, 182(3):243–252, 1996.

[16] D. A. Beard, S. Liang, and H. Qian. Energy balance for analysis of complex metabolic networks. *Biophysical Journal*, 83(1):79–86, 2002.

[17] A. Hoppe, S. Hoffmann, and H.-G. Holzhütter. Including metabolite concentrations into flux balance analysis: thermodynamic realizability as a constraint on flux distributions in metabolic networks. *BMC Systems Biology*, 1(1):1–12, 2007.

[18] T. Lubitz, M. Schulz, E. Klipp, and W. Liebermeister. Parameter balancing for kinetic models of cell metabolism. *J. Phys. Chem. B*, 114(49):16298–16303, 2010.

[19] N.J. Stanford, T. Lubitz, K. Smallbone, E. Klipp, P. Mendes, and W. Liebermeister. Systematic construction of kinetic models from genome-scale metabolic networks. *PLoS ONE*, 8(11):e79195, 2013.

[20] E. Noor, H.S. Haraldsdottir, R. Milo, and R.M.T. Fleming. Consistent estimation of Gibbs energy using component contributions. *PLOS Comp. Biol.*, 9:e1003098, 2013.

[21] I. Schomburg, A. Chang, C. Ebeling, M. Gremse, C. Heldt, G. Huhn, and D. Schomburg. BRENDA, the enzyme database: updates and major new developments. *Nucleic Acids Research*, 32:Database issue:D431–433, 2004.

[22] B.R.B.H. van Rijsewijk, A. Nanchen, S. Nallet, R.J. Kleijn, and U. Sauer. Large-scale 13c-flux analysis reveals distinct transcriptional control of respiratory and fermentative metabolism in escherichia coli. *Mol. Syst. Biol.*, 7(477):477, 2011.

[23] L. Gerosa, B.R.B.H. van Rijsewijk, D. Christodoulou, K. Kochanowski, T.S.B. Schmidt, E. Noor, and U. Sauer. Pseudo-transition analysis identifies the governing regulation of microbial nutrient adaptations from steady state data. *Cell Systems*, 1:270–282, 2015.

[24] A. Schmidt, K. Kochanowski, S. Vedelaar, E. Ahrné, B. Volkmer, L. Callipo, K. Knoops, M. Bauer, R. Aebersold, and M. Heinemann. The quantitative and condition-dependent escherichia coli proteome. *Nature Biotechnology*, page doi:10.1038/nbt.3418, 2015.

[25] M. Kanehisa, S. Goto, S. Kawashima S, and A. Nakaya. The KEGG databases at genomenet. *Nucleic Acids Research*, 30:42–46, 2002.

[26] L. Arike, K. Valgepea, L. Peil, R. Nahku, K. Adamberg, and R. Vilu. Comparison and applications of label-free absolute proteome quantification methods on Escherichia coli. *J Proteomics*, 75(17):5437–5448, 2012.
